# EGaIn‐Activated Bioinspired Silk Micro/Nanofibril Eutectogels Breaking the Strength–Conductivity Trade‐Off for High‐Performance Wearable Bioelectronics

**DOI:** 10.1002/advs.202520723

**Published:** 2026-01-04

**Authors:** Haiwei Yang, Dongdong Ye, Yezi You, Ming Fu, Zongqian Wang

**Affiliations:** ^1^ Department of Polymer Science and Engineering University of Science and Technology of China Hefei Anhui China; ^2^ School of Textile and Garment Innovation Center for Anhui Ecological Textile Printing and Dyeing Manufacturing Industry Anhui Polytechnic University Wuhu Anhui China; ^3^ Key Laboratory of Textile Fiber and Products (Ministry of Education) Wuhan Textile University Wuhan China; ^4^ School of Materials and Chemistry Anhui Agricultural University Hefei Anhui China; ^5^ Hefei Institute For Public Safety Research Tsinghua University Hefei Anhui China

**Keywords:** EGaIn, eutectogel, mechanical property, silk, wearable bioelectronic

## Abstract

Eutectogels combining high mechanical and electrical performance hold great promise for next‐generation wearable electronics. However, conventional polymerizable deep eutectic solvent (PDES)–based eutectogels suffer from an inherent strength–conductivity trade‐off. Here, inspired by the multiscale architecture of the extracellular matrix, a bioinspired strategy is developed by integrating silk micro/nanofibrils (SMNF) as a reinforcing scaffold within a choline chloride/acrylic acid PDES. SMNF are generated in situ via deconstruction of silk fibers, while eutectic gallium–indium (EGaIn) microdroplets initiate polymerization without toxic initiators or high‐energy UV irradiation, enabling one‐step fabrication of SMNF‐reinforced eutectogels (SMNF‐Egel). The resulting SMNF‐Egel combines dynamic hydrogen and coordination bonding with a robust micro/nanofibrous network, achieving a tensile strength of 1.25 MPa, toughness of 23.09 MJ m^−3^, fracture strain of 2289%, and conductivity of 1.51 S m^−1^, alongside skin‐like modulus, self‐healing, and environmental stability. These properties enable ultrasensitive strain sensing, Morse code communication, and stable bioelectrical signal monitoring. This work establishes a sustainable route to high‐performance silk‐based eutectogels and provides a versatile platform for advanced wearable sensors and bioelectronic interfaces.

## Graphical abstract

Inspired by the extracellular matrix, eutectogels are prepared by in situ deconstruction of silk fibroin into micro/nanofibrils and EGaIn‐induced polymerization. The multiscale fibril network with dynamic crosslinking achieves high strength (1.25 MPa), toughness (23.09 MJ m^−3^), and conductivity (1.51 S m^−1^), outperforming previous natural polymer–reinforced eutectogels and breaking the strength–toughness–conductivity trade‐off for wearable sensors and bioelectrodes.

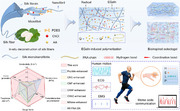

## 1 Introduction

Flexible wearable electronic devices have garnered significant attention in recent years due to their promising applications in physiological monitoring, intelligent healthcare, and human‐machine interfaces [1, 2]. As a key material, ionogels play an essential role in advancing high‑performance flexible sensors and bioelectronic interfaces, benefiting from their biomimetic 3D polymer frameworks, high ionic conductivity, and intrinsic mechanical compliance [3, 4]. Despite these advantages, conventional ion‑conductive gels—including ion‐conductive hydrogels and ionic liquid‐based gels—exhibit inherent performance limitations that restrict their practical implementation [5]. Hydrogels suffer from water freezing at low temperatures, leading to loss of flexibility and conductivity, while water evaporation under ambient conditions causes mechanical and electrical instability [6]. Ionic liquid‐based gels offer enhanced environmental stability; however, their complex synthesis, high cost, and potential toxicity hinder large‑scale application [7].

Eutectogels, constructed through the synergistic integration of cross‐linked polymer networks and deep eutectic solvents (DES), have emerged as a promising alternative [8]. They possess several unique advantages: (1) broad temperature tolerance resulting from the low melting points and high thermal stability of DES, enabling stable performance across sub‐zero to elevated temperatures; (2) non‐volatile nature, which ensures long‐term structural and conductive stability; (3) biocompatibility and sustainability, as many DES consist of natural, biodegradable, and low‐toxicity constituents; and (4) tunable ionic conductivity adjustable via DES composition and hydrogen‐bond interactions [9, 10, 11]. These distinct merits position eutectogels as the next‐generation flexible electronic material platform capable of surpassing traditional ion‑conductive gels. Among various fabrication routes, the in situ radical polymerization of polymerizable DES (PDES) has become a common strategy because of its controllability and operational simplicity [12, 13, 14]. Nevertheless, most PDES‐based eutectogels rely primarily on weak dynamic interactions—such as reversible hydrogen bonding—which lead to low cross‐linking density [5, 10, 15]. This limitation imposes a trade‐off between mechanical strength, toughness, and ionic conductivity, with reported strengths typically below 0.5 MPa, toughness under 15 MJ m^−3^, and conductivities rarely exceeding 1 S m^−1^, all insufficient for demanding flexible wearable applications [16, 17, 18].

To overcome these challenges, various strategies—including physical or chemical crosslinking [19, 20], double‑network construction [21, 22], and nanocomposite reinforcement using materials such as nanocellulose or MXene [23, 24]—have been explored. Although these methods improve strength and toughness, they often compromise ionic conductivity, making it difficult to achieve balanced performance. Recent efforts have also incorporated regenerated proteins or cellulose networks, but limited hierarchical integration hampers toughness improvement [25, 26]. In contrast, the extracellular matrix (ECM) of biological tissues provides an inspiring model, exhibiting a synergistic combination of high strength, toughness, and efficient ion transport arising from its sophisticated multiscale architecture [27]. The ECM features hierarchical networks of nanoscale to microscale fibrous proteins interconnected by dynamic crosslinks, enabling effective energy dissipation while maintaining structural integrity and supporting physiological communication [28, 29]. Inspired by these natural architectures, micro/nanofibrils such as cellulose microfibrils, cellulose nanofibrils, and chitin nanofibrils have been investigated to reinforce eutectogels [24, 30, 31]. However, their integration typically involves complex processes, toxic initiators, or high‑energy UV irradiation, presenting challenges for green, scalable manufacturing. Therefore, developing scalable, green, in situ strategies to construct multiscale fiber networks within eutectogels and overcome the incompatibility of strength, toughness, and conductivity remains a critical challenge in the field.

Here, we propose a biomimetic hierarchical eutectogel fabrication strategy fundamentally distinct from previous approaches that simply blend pre‑fabricated nanofibers into polymer matrices. The choline chloride/acrylic acid PDES is employed to thermally induce the in situ deconstruction of silk fibroin (SF) fibers into silk micro/nanofibrils (SMNF), mimicking the hierarchical fibrillogenesis observed in the native ECM. Subsequently, eutectic gallium–indium (EGaIn) microdroplets are utilized to catalyze the in situ polymerization of acrylic acid, eliminating the need for external initiators or UV irradiation and thereby simplifying the preparation process while ensuring green and efficient network formation. The resulting hierarchical eutectogel (SMNF‐Egel) integrates multiscale fibril networks and dynamic molecular crosslinking, demonstrating high mechanical strength (1.25 MPa), outstanding toughness (23.09 MJ m^−3^), ultra‐high elongation at break (2289%), and high ionic conductivity (1.51 S m^−1^), along with excellent environmental stability and self‐healing capability. This approach overcomes traditional trade‐offs and offers a high‐performance, environmentally friendly, and easily integrated material solution for flexible sensors and bioelectronics, significantly expanding the application prospects of silk fibroin‐based eutectogels in flexible electronics and smart healthcare.

## 2 Results and Discussion

### 2.1 Design of Biomimetic SMNF‐Egel

**FIGURE 1:**
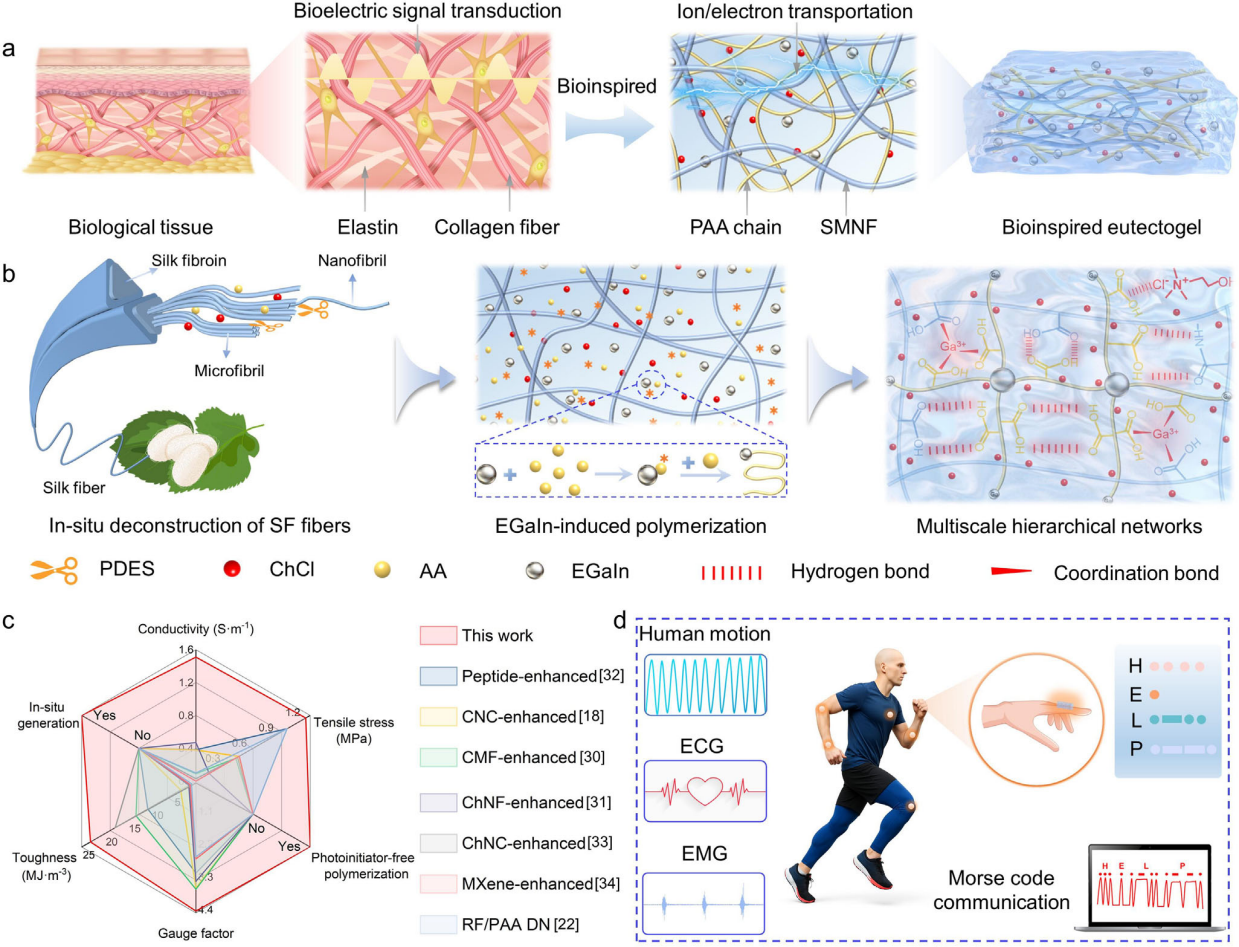
Design and application of biomimetic SMNF‐Egel. (a) Schematic illustration of the structure of biological tissue and SMNF‐Egel. (b) Schematic illustration of the synergistic strategy in which PDES induces the in situ deconstruction of SF fibers while EGaIn microdroplets catalyze polymerization, enabling the fabrication of biomimetic SMNF‑Egel. (c) Comparison of the SMNF‑Egel with recently reported eutectogels enhanced through other approaches (such as nanocomposite and dual‑network strategies) in terms of mechanical performance, ionic conductivity, sensing sensitivity, and processing advantages. (d) Applications of SMNF‐Egel in human motion monitoring, physiological electrical signal (including EMG and ECG) detection, and Morse code communication.

As illustrated in Figure 1a, the ECM features a characteristic multiscale fibrous architecture in which nanoscale collagen fibrils assemble into microscale fiber bundles interwoven with the elastin networks. This hierarchical organization, reinforced by dynamic interactions such as hydrogen bonding, ionic bonding, and coordination interactions, collectively imparts biological tissues with exceptional mechanical robustness and efficient bioelectric signal transduction [27, 28, 29]. Inspired by the hierarchical architecture of the ECM, we developed a novel strategy that integrates in situ deconstruction of SF fibers and EGaIn‐initiated polymerization within a PDES system composed of choline chloride (ChCl)/ acrylic acid (AA) (Figure 1b). Specifically, thermal treatment of PDES is used to induce in situ deconstruction of SF fibers (discussed in detail later), generating a highly dispersed SMNF network with strong interfacial bonding and an inherent hierarchical structure ranging from nanofibrils to microfibrils. These SMNFs serve as reinforcing phases analogous to the micro‑ and nanoscale collagen fibril networks in the ECM. Subsequently, EGaIn microdroplets catalyze the rapid polymerization of AA monomers within the SMNF‑PDES system to form the SMNF‑based eutectogel (SMNF‑Egel), in which the SMNF network is seamlessly embedded within the polymerized PDES matrix, similar to how proteoglycans integrate with fibrous networks in the ECM. Meanwhile, dynamic hydrogen bonds among polyacrylic acid (PAA), ChCl, and SMNF, together with Ga^3+^–carboxyl coordination interactions, emulate the reversible bonding motifs of the ECM, establishing efficient pathways for energy dissipation. As a result, the synergy between this ECM‑inspired micro/nanofibrillar network and dynamic molecular cross‐linking enables SMNF‑Egel to achieve outstanding combined properties, including high tensile strength (1.25 MPa), remarkable toughness (23.09 MJ m^−3^), large fracture elongation (2,289%), and excellent ionic conductivity (1.51 S m^−1^).

Compared with conventional approaches that construct micro/nanocomposite structures or dual networks (DN) through externally added reinforcing components [18, 22, 30, 31, 32, 33, 34] (Figure 1c; Table S1), our approach of generating SMNFs in situ not only addresses the long‑standing trade‑off between mechanical strength and ionic conductivity, but also ensures excellent interfacial compatibility and strong bonding between the fibrillar network and the gel matrix, thereby avoiding the agglomeration problems that frequently arise with externally introduced micro/nanofillers. This stands in clear contrast to high‐crystallinity PVA‐based eutectogels [35], which typically exhibit excellent mechanical properties (e.g., strength up to 20.2 MPa) but limited ionic conductivity (∼0.0624 S m^−1^); our SMNF‐Egel successfully breaks this classical trade‐off through its biomimetic hierarchical design, enabling concurrent high strength, toughness, and ionic conductivity. Importantly, the unique EGaIn‑induced polymerization mechanism eliminates the need for additional initiators, cross‐linkers, or UV irradiation, significantly simplifying the fabrication process while improving environmental sustainability. Owing to its multiscale hierarchical fibril network and outstanding mechanical–electrical performance, the SMNF‑Egel shows strong potential for practical applications in flexible sensing (Figure 1d). On the one hand, the dissipative structure formed by the cooperation of micro/nanofibril networks and dynamic bonding interactions enables stable and rapid electrical responses under deformation, making the material suitable for highly sensitive strain sensors capable of real‑time human motion monitoring and coded signal transmission. On the other hand, the excellent interfacial compatibility and low impedance of SMNF‑Egel support its use as bioelectrodes for high‑fidelity acquisition of physiological signals, such as electromyography (EMG) and electrocardiography (ECG).

### 2.2 Deconstruction of SF Fibers by PDES and Molecular Mechanisms

**FIGURE 2:**
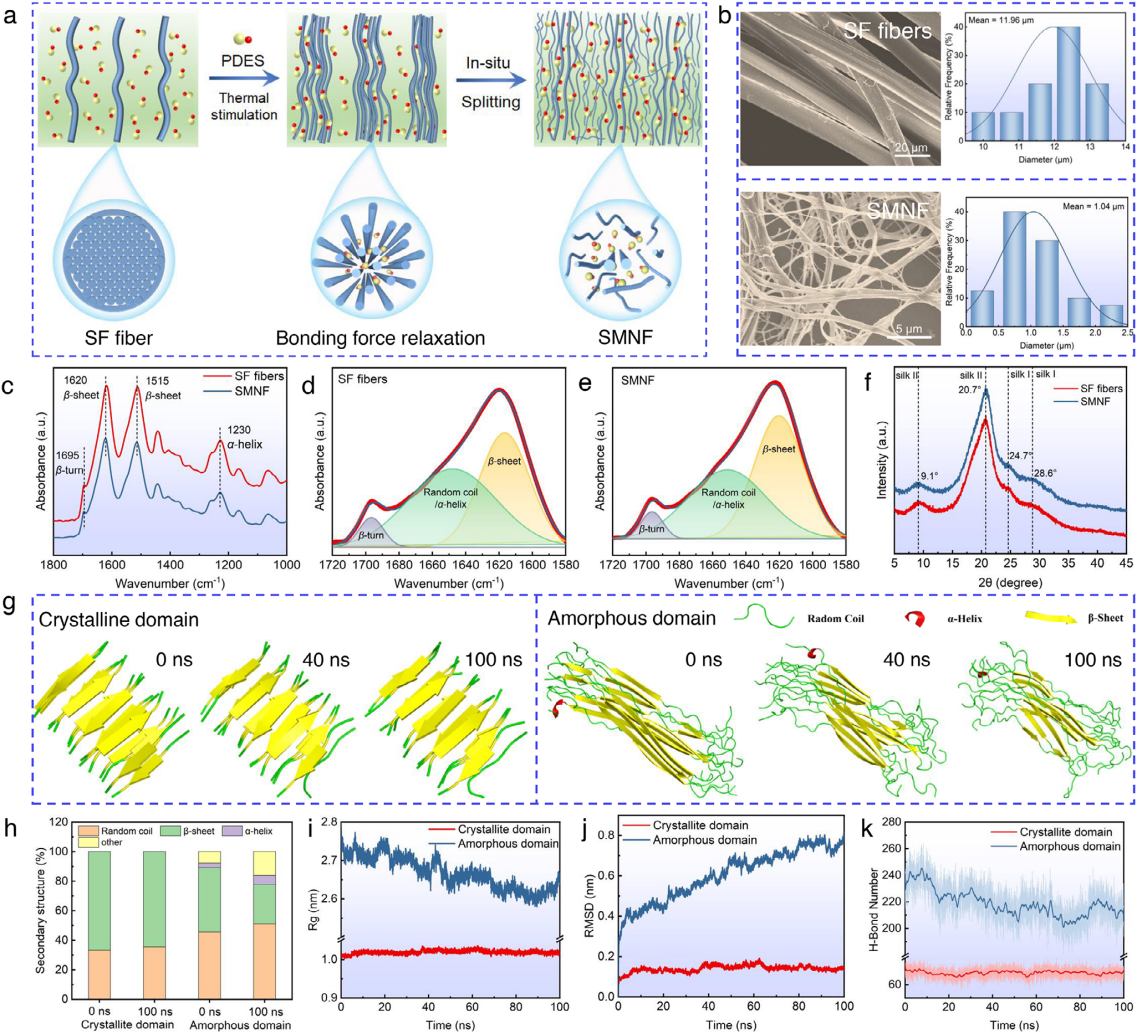
In situ deconstruction of SF fibers by PDES and the underlying mechanism. (a) Schematic illustration of the thermal‐stimulated in situ deconstruction of SF fibers by PDES to form SMNF. (b) SEM images and diameter distributions of SF fibers and SMNF. (c) FTIR spectra, (d–e) deconvolution results of the amide I band in the FTIR spectra, and f) XRD spectra of SF fibers and SMNF. (g) Representative snapshots of the crystallite and amorphous domains after molecular dynamics simulations at different time points. (h) Changes in the secondary structure of crystalline and amorphous domains before and after 100 ns of molecular dynamics simulation. (i) Radius of gyration (Rg), (j) root mean square deviation (RMSD), and k) H‐Bond number in crystalline and amorphous domains during 100 ns of molecular dynamics simulation.

To fabricate a eutectogel with bioinspired structure featuring multiscale fibril networks, a critical step is to select an appropriate natural fiber and transform it into micro‐ and nanofibrils, thereby constructing multiscale structures as reinforcing phases [36, 37]. SF fiber, a bioactive, renewable biopolymer, was selected to reconstruct the eutectogel network due to its sophisticated hierarchical architecture, which includes microfibrils, nanofibrils, and molecular‐scale polypeptide chains [38, 39]. This hierarchical structure endows SF fibers with outstanding mechanical properties, such as high tensile strength (0.3–1.3 GPa) and exceptional toughness (70–200 MJ m^−3^), surpassing those of cellulose and chitin fibers [40, 41, 42]. SMNF produced via appropriate deconstruction methods retains the original mesostructure of natural SF fibers, making them desirable building blocks for high‐performance biomimetic eutectogels. However, chemically harsh or energy‐intensive processes often hinder the micro‐ and nanofibrillation of SF fibers [43]. Moreover, the extracted silk micro/nanofibrils are typically challenging to redisperse uniformly in deep eutectic solvents [44]. In this study, we employed a PDES composed of ChCl and AA to thermally stimulate the in situ deconstruction of SF fibers, generating SMNF as the reinforced phase within the eutectogel (Figure 2a). Compared to eutectogels reinforced by externally added micro‐ and nanofibrils [24, 31, 45], our approach emphasizes the in situ formation of silk micro/nanofibrils within the PDES system, enabling seamless integration of the fibrils with the gel matrix during subsequent polymerization. This strategy imparts the material with excellent interfacial compatibility and enhanced mechanical properties.

First, PDES was synthesized by mixing ChCl and AA at a molar ratio of 1:2 at 90°C, yielding a transparent liquid after cooling to room temperature (Figure S1). Fourier transform infrared spectroscopy (FTIR) and proton nuclear magnetic resonance (^1^H NMR) analysis confirmed the formation of strong hydrogen bonds between ChCl and AA in the system (Figure S2a–c). These strong hydrogen bonds imparted the PEDS with excellent anti‐freezing performance, enabling it to remain in a liquid state even at −20°C (Figure S2d,e). After stirring for 5 h at 100°C, a completely dispersed SMNF‐PDES mixture was obtained (Figure S3). PDES effectively disrupted the hydrogen bonds and hydrophobic interactions between SF molecules [43, 44], deconstructing SF fibers (average diameter: 11.96 µm) into smaller SMNF (average diameter: 1.04 µm) (Figure 2b). This process prevented fiber agglomeration and promoted uniform dispersion in the PDES. Notably, although increasing the temperature and prolonging the reaction time can enhance the deconstruction efficiency of SF fibers by PEDS, excessive conditions may damage their multiscale hierarchical structure. Therefore, we systematically investigated the effects of heating temperature and duration on the deconstruction process of SF fibers by the PDES (Details in Figure S4).

To investigate the molecular mechanism underlying the deconstruction of SF fibers by PDES, FTIR spectroscopy was applied to analyze the molecular conformation of SMNF and SF fibers. The results revealed that SMNF exhibited characteristic peaks of *β*‐sheet and *β*‐turn structures at 1620 and 1695 cm^−1^ [43, 46], respectively, in the amide I band (Figure 2c). Additionally, an absorption peak at approximately 1515 cm^−1^ in the amide II band was attributed to the *β*‐sheet structure, while the peak at around 1230 cm^−1^ in the amide III band corresponded to the *α*‐helix structure [44, 47]. The major characteristic peaks of SMNF were largely consistent with those of SF fibers, indicating that both materials possess similar secondary structures. Peak deconvolution analysis of the amide I band demonstrated that the *β*‐sheet content in SMNF increased from 43.09% (in SF fibers) to 50.37%, whereas the combined content of random coil and *α*‐helix decreased from 51.46% to 44.57% (Figure 2d,e). These results suggest that PDES primarily disrupts the molecular network of the amorphous regions in SF fibers during the deconstruction process, while exerting minimal effect on the highly ordered *β*‐sheet crystalline regions. Further analysis using X‐ray diffraction (XRD) showed that both SMNF and SF fibers exhibited characteristic peaks of silk II at 2θ ≈ 9.1° and 20.7° [35], as well as peaks of silk I at 2θ ≈ 24.7° and 28.6° [48, 49], indicating that PDES effectively weakened the interfacial interactions between fibers without significantly altering their internal microstructure (Figure 2f). Additionally, XRD peak deconvolution revealed that the crystallinity of SMNF was 57.57%, slightly higher than SF fibers (52.65%), further confirming that PDES did not adversely affect the *β*‐sheet structure during the deconstruction process (Figure S5).

Molecular dynamics simulations were performed on representative models of both crystalline and amorphous SF domains to investigate the interaction mechanism of PDES (Figure 2g–k). Detailed methods for model construction and simulation are provided in the Supporting Information. The crystalline SF structure was obtained from the Protein Data Bank (PDB: 2SLK), and the validity of the constructed amorphous model is discussed in Figure S6. As shown in Figure 2g, the crystalline domain maintained high conformational stability throughout 100 ns, with *β*‐sheet structures remaining intact. In contrast, the amorphous domain underwent substantial conformational relaxation in PDES, characterized by reduced *β*‐sheet content, increased random coils, and progressive chain loosening, indicating significant disruption of its structural stability. These findings are supported by the time evolution of secondary structures (Figure S7) and quantified by the changes in secondary structure ratios (Figure 2h): the *β*‐sheet content in the crystalline domain remained above 64%, while decreasing from 43.6% to 26.7% in the amorphous domain; random coil and *α*‐helix content increased from 48.6% to 57.2%. Additionally, structural stability differences were confirmed by analyses of radius of gyration (*R*_g_), root mean square deviation (RMSD), and hydrogen bond numbers (Figure 2i–k) [50, 51]. Therefore, PDES preferentially disrupts the less ordered amorphous domain while leaving the crystalline *β*‐sheet‐rich regions largely unaffected, which is consistent with polymer physics principles. The amorphous domain's higher free volume enables PDES molecules to penetrate and disrupt SF networks, whereas the crystalline domain's dense, hydrophobic, and highly ordered *β*‐sheet structures resist such disruption.

Integrating experimental data with molecular dynamics simulations, we propose an evolution model illustrating the in situ formation of SMNF from PDES‐deconstructed SF fibers (Figure S8a). Surface defects on SF fibers generated during degumming (Figure S8b) allow PDES to penetrate the amorphous regions, disrupt intermolecular hydrogen bonds, and form solvation layers around amorphous SF molecules. This facilitates dissolution of SF networks and fiber swelling (Figure S8c). As a result, interfibrillar interactions weaken, leading to in situ splitting of SF fibers (Figure S8d), which are further deconstructed by PDES into micro‐nanofibrils while preserving their mesoscale structures (Figure S8e).

### 2.3 EGaIn‐Activated Polymerization of SMNF‐Egel

**FIGURE 3:**
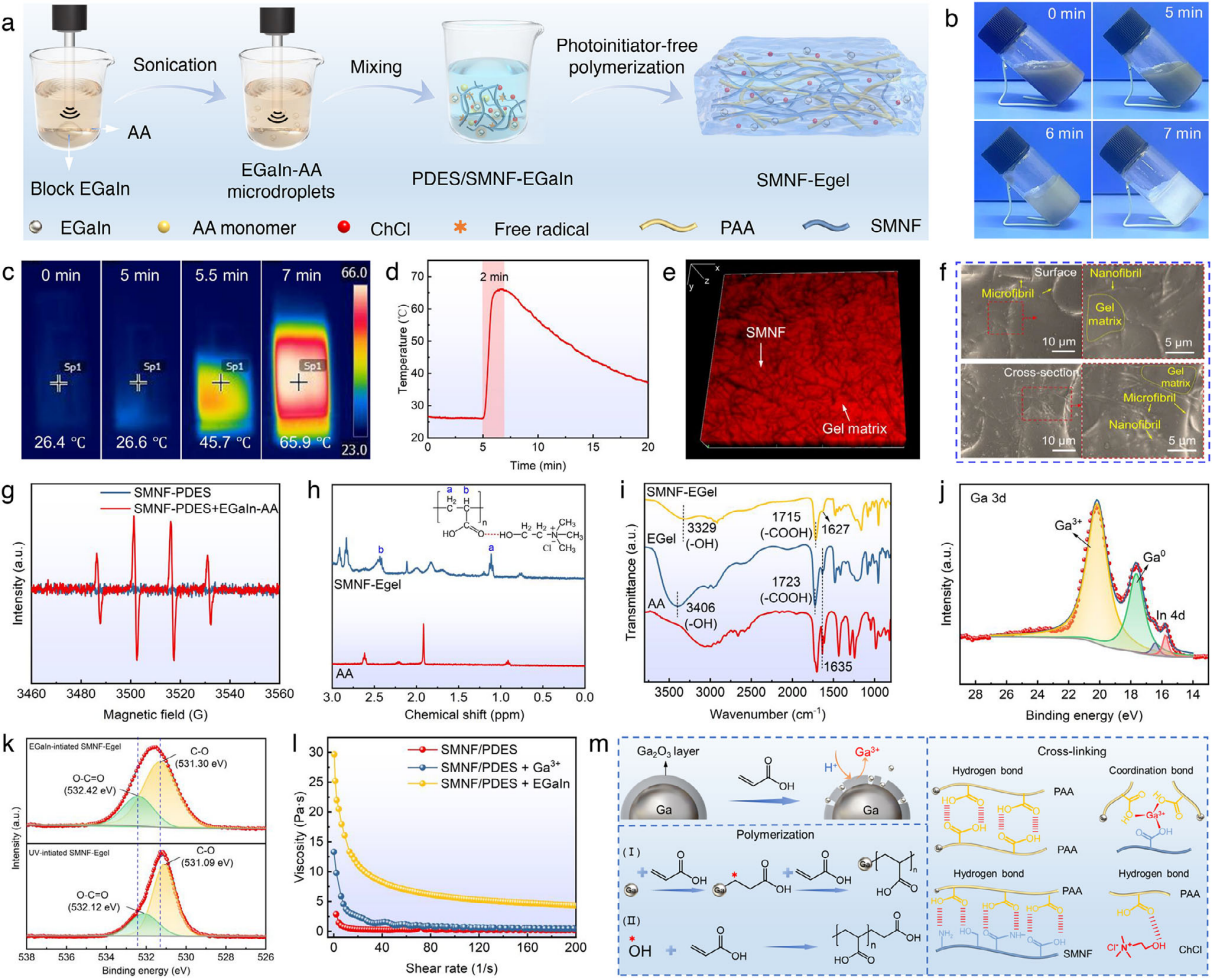
Fabrication and characterization of SMNF‐Egel. (a) Schematic illustration of the SMNF‐Egel fabrication process. (b) Optical images of the gelation process. (c) Infrared thermal images and (d) temperature‐time profile during gelation. (e) 3D laser scanning confocal and (f) SEM images of SMNF‐Egel. (g) EPR spectra of SMNF‐PDES before and after addition of EGaIn‐AA microdroplets. (h) ^1^H NMR spectra of SMNF‐Egel and AA. (i) FTIR spectra of SMNF‐Egel, Egel, and AA. High‐resolution XPS spectra of (j) Ga 3d and (k) O 1s. (l) Viscosity‐shear rate curves for SMNF‐PDES, SMNF‐PDES/Ga^3+^, and SMNF‐PDES/EGaIn‐AA. (m) Schematic illustration of EGaIn‐initiated polymerization and cross‐linking mechanism for gel network formation.

The unpaired electrons of Ga atoms in EGaIn exhibit high chemical reactivity, initiating the radical polymerization of vinyl monomers [52, 53]. In our biomimetic design, EGaIn–AA microdroplets served as initiators that directly triggered the radical polymerization of AA monomers within the SMNF‑PDES mixture to form SMNF‑Egel (Figure 3a). The selection of EGaIn amount depends on ensuring effective polymerization while avoiding excess that may lead to microdroplet aggregation. To achieve this, homogeneous EGaIn–AA microdroplet suspensions with an average size of 2.51 µm were prepared by sonicating 0.7 g of bulk EGaIn in 14 g of AA (Figure S9). Mixing 3.6 g of this suspension with 14 g of the SMNF‑PDES mixture produced SMNF‑Egel, a formulation optimized through preliminary experiments and supported by literature [54], confirming its ability to effectively initiate polymerization under mild and environmentally friendly conditions. Increasing the EGaIn content beyond this level tends to cause aggregation of microdroplets (Figure S10), which compromises gel homogeneity and introduces excessive Ga^3+^ coordination sites that may lead to over‑crosslinking, thereby reducing ductility and hindering ion migration [54]. It also unnecessarily raises material consumption and reduces sustainability, which runs counter to the environmentally conscious design principles of this study.

The polymerization initiated by EGaIn proceeds rapidly at room temperature. Once the EGaIn–AA microdroplets are mixed with the SMNF‑PDES mixture, radical generation begins immediately, as confirmed by dye‑degradation experiments using methyl orange (MO). As shown in Figure S11, Videos S1 and S2, the acidified SMNF‑PDES mixture maintained its red color upon MO addition, whereas the color in the SMNF‑PDES/EGaIn‑AA mixture gradually faded, indicating active radical formation [52, 55]. The polymerization enters the propagation stage at approximately 5 min, accompanied by a rapid temperature rise. Thermal imaging reveals that polymerization first occurs locally and then spreads as heat diffuses throughout the system (Figure 3c), a behavior characteristic of free‑radical chain initiation and propagation [52, 54]. The temperature increases from 26.6°C to 65.9°C within 2 min (Figure 3d). During this period, the viscosity of the system steadily increases and even shows a typical filament‑drawing behavior (Figure S12a,b), reflecting the progression of chain growth and network formation. Gelation begins at around 5 min and reaches completion by 7 min, resulting in a stable gel at room temperature. This transition is evidenced by the loss of fluidity and the formation of a solid eutectogel (Figure 3b; Figure S12c). Under these conditions, the total gelation time is approximately 7 min. Such a rapid, controllable, and mild polymerization underscores the scalability and reproducibility of our fabrication method.

The microstructure of SMNF‐Egel was analyzed by laser scanning confocal microscopy (LSCM) and scanning electron microscopy (SEM). LSCM revealed that SMNF‐Egel possessed a multiscale micro/nanofibril‐reinforced composite architecture, in contrast to the eutectogel without SMNF (Egel) (Figure S13). Thereof, the SMNF (black) was uniformly distributed within the gel matrix (red), forming a well‐dispersed, tightly interwoven 3D network (Figure 3e). SEM observations revealed that the silk micro‑ and nanofibrils (highlighted by yellow arrows) were uniformly embedded within the continuous gel matrix and intertwined to form a multiscale fibrillar network (Figure 3f). This hierarchical architecture provides an effective reinforcing scaffold, which is expected to synergistically enhance both the mechanical performance and ionic conductivity of the SMNF‑Egel. Moreover, elemental mapping confirmed uniform distribution of Ga, In, C, O, N, and Cl within the matrix (Figure S14), suggesting no significant EGaIn aggregation.

To further elucidate the EGaIn‑induced polymerization mechanism, electron paramagnetic resonance (EPR) spectroscopy was employed to detect free radicals formed in the SMNF‑PDES/EGaIn‑AA system. As shown in Figure 3g, the spectrum displays a characteristic 1:2:2:1 quartet, confirming the presence of ·OH radicals. These radicals originate from carbon‑centered species generated through the interaction between unpaired electrons on Ga and the π bonds of AA, which subsequently react with trace amounts of water and dissolved oxygen [56, 57]. The chemical structure of SMNF‐Egel, characterized by ^1^H NMR and FTIR, showed peaks at 1.11 and 2.42 ppm in Figure 3h corresponding to methylene and methine protons of the PAA backbone [54], and peaks at 3.21, 3.54, and 4.07 ppm (Figure S15) assigned to ChCl hydrogen‐bonded to PAA [23, 54]. FTIR spectra showed the disappearance of the C═C peak at 1635 cm^−1^ (Figure 3i), indicating complete AA polymerization [58]; shifts of ─OH and ─COOH peaks to lower wavenumbers and a new peak at 1627 cm^−1^ reflected strong hydrogen bonding between SMNF and the PDES matrix and Ga^3+^–carboxyl coordination [55], respectively. Wide‑range X‑ray photoelectron spectroscopy (XPS) of the EGaIn‑initiated SMNF‑Egel revealed new peaks corresponding to Ga 3d, Ga 2p, and In 3d (Figure S16). In addition, the high‑resolution Ga 3d spectrum exhibited distinct signals for both Ga^3+^ and Ga^0^ (Figure 3j), confirming the coexistence of oxidized and metallic gallium species and demonstrating the formation of Ga^3^⁺‑mediated coordination bonds [52, 55]. The high‑resolution O1s spectrum further supports this conclusion: the O─C═O and C─O peaks in the EGaIn‑initiated SMNF‑Egel shifted to higher binding energies compared with those in the UV‑initiated SMNF‑Egel (Figure 3k). Such shifts indicate strengthened interactions between Ga^3+^ and carboxyl groups, providing additional evidence for coordination‑bond formation within the gel network [59]. The addition of Ga^3+^ standard solution to SMNF‐PDES increased viscosity (Figure 3l) while maintaining fluidity (Figure S17), suggesting that Ga^3+^ plays only a cross‐linking role, whereas metallic Ga initiates polymerization. As summarized in Figure 3m, under acidic conditions, H^+^ disrupts the Ga_2_O_3_ layer on EGaIn, generating Ga^3+^ ions and facilitating EGaIn dispersion [54]; AA monomers polymerize via carbon‐centered and ·OH radicals [52, 55], and the resulting PAA carboxyl groups form hydrogen bonds with SMNF, ChCl, and other PAA chains, while Ga^3+^ coordinates with carboxyl groups of SMNF and PAA to form coordination cross‐links. Consequently, SMNF‐Egel exhibits a dynamically cross‐linked network reinforced by hydrogen bonds and Ga^3+^ coordination, significantly enhancing its structural integrity and mechanical properties.

### 2.4 Enhanced Mechanical and Electrical Properties of SMNF‐Egel

**FIGURE 4:**
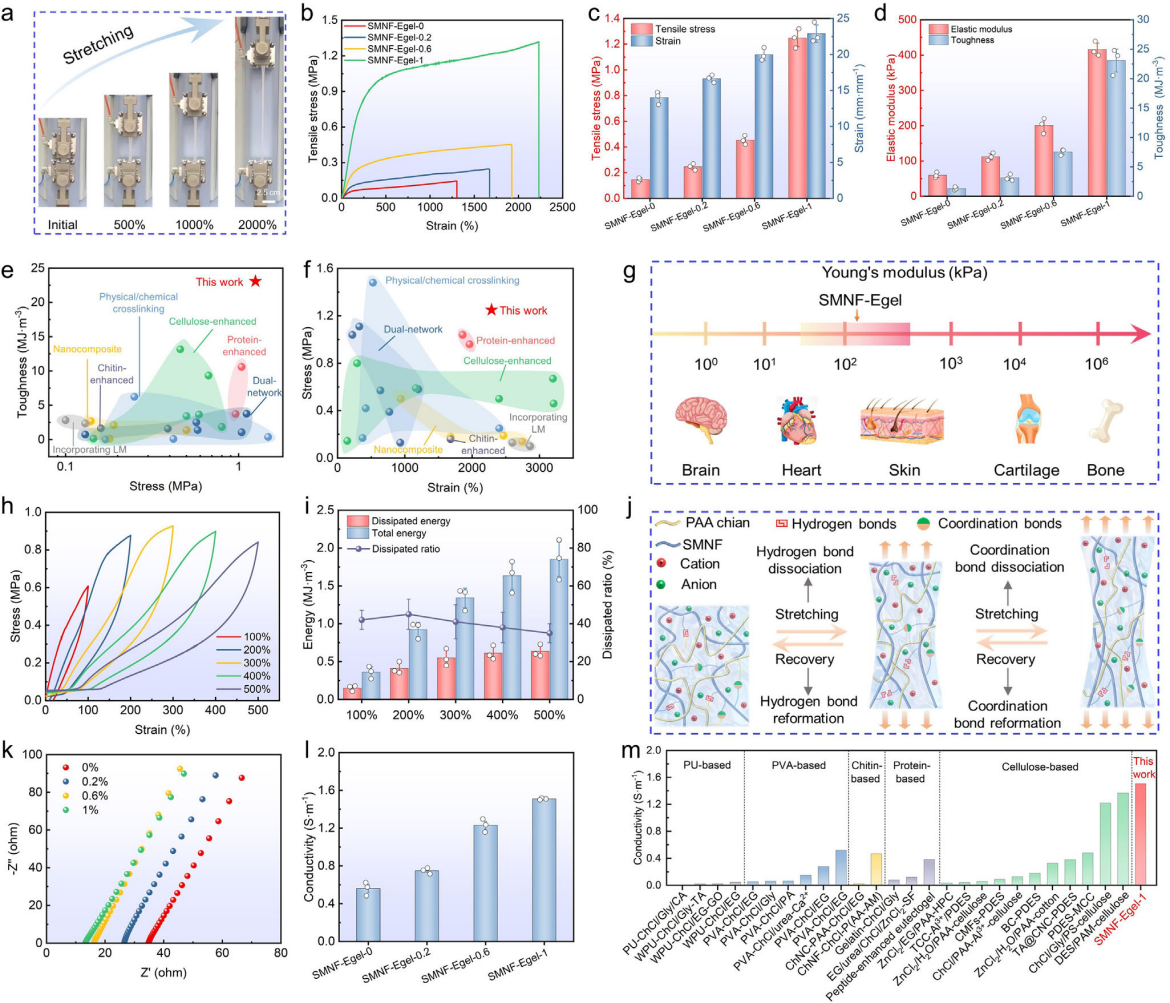
Mechanical and electrical properties of SMNF‐Egel. (a) Digital images of SMNF‐Egel‐1 under different tensile strains. (b) Stress–strain curves and (c,d) corresponding tensile stress, strain, modulus, and toughness demonstrating the impact of SMNF content on mechanical performance. (e,f) The Ashby plots comparing the stress, strain, and toughness of SMNF‐Egel‐1 to the reported PDES‐based eutectogels. (g) Young's modulus of human tissues, organs, and SMNF‐Egel. (h,i) Cyclic loading‐unloading tests at various strains, along with calculations of total and dissipated energy and dissipation ratio, reveal robust energy dissipation capability. (j) The schematic illustration of the underlying energy dissipation mechanism. (k,l) Nyquist plots and conductivity data showing the dependence of electrical properties on SMNF content. (m) Comparison of the conductivity between our work and other reported representative eutectogels. Data in (c,d,i,l) are presented as mean ± SD, n = 3.

To evaluate the effect of SMNF content on the mechanical and electrical properties of SMNF‐Egel, SF fibers at varying concentrations (0%, 0.2%, 0.6%, and 1% of PDES mass) were in situ deconstructed by PDES, as higher loadings (e.g., 1.4%) led to poor dispersion and processing issues (Figure S18). This limit is inherently governed by the maximum deconstruction capacity of SF fibers in the PDES system, beyond which fibril aggregation occurs and compromises the homogeneous multiscale network. Increasing SMNF content significantly enhanced the stretchability, tensile strength, and toughness of SMNF‐Egel. Specifically, SMNF‐Egel‐1 exhibited an elongation at break of up to 2000%, a tensile strength increasing from 0.15  to 1.25 MPa, and a toughness of 23.09 MJ m^−3^, which is 18 times that of the control (Figure 4a–d). These mechanical improvements are attributed to the multiscale reinforcement provided by micro/nanofibrils and the enhanced interfacial compatibility via supramolecular interactions among SF, PAA, and Ga^3+^ [36, 55]. Compared to other PDES‐based eutectogels, SMNF‐Egel‐1 outperformed in tensile strength, fracture strain, and toughness (Figure 4e,f; Figure S19, and Table S2). Furthermore, the Young's modulus (59.7–416.2 kPa) closely matched that of human skin (Figure 4g), ensuring both comfort and long‐term stability for SMNF‐Egel‐based wearable electronics [60, 61].

Continuous tensile loading‐unloading tests were conducted at strains ranging from 100% to 500% to investigate the energy dissipation behavior of SMNF‐Egel‐1. SMNF‐Egel‐1 exhibited pronounced hysteresis loops during each loading‐unloading cycle (Figure 4h), indicating efficient energy dissipation. As strain increased from 100% to 500%, the dissipated energy per unit volume significantly rose from 152.2 to 635.3 kJ m^−3^ (Figure 4i), thereby enhancing the toughness of SMNF‐Egel‐1. Notably, subsequent loading curves consistently surpassed the unloading curves of the previous cycle (Figure 4h), suggesting effective reassociation through abundant reversible interactions [30] and, consequently, superior self‐recovery capability. Cyclic tensile loading‐unloading tests at 100% strain with varying residence times were performed to further validate this self‐recovery. Figure S20a shows that the stress–strain curves gradually converged toward the original loading path as residence time increased from 0 to 5 min, demonstrating excellent elastic recovery. Remarkably, after 100 cycles of loading and unloading at 100% strain, SMNF‐Egel‐1 nearly recovered to its initial length following a 30 min rest (Figure S20b). Additionally, mechanical hysteresis was observed in cyclic loading‐unloading without resting periods at a fixed 100% tensile strain (Figure S21a). The dissipated energy in the first cycle (104.3 kJ m^−3^) was 2.3 and 4.8 times that of the second (45.4 kJ m^−3^) and twentieth (21.8 kJ m^−3^) cycles, respectively (Figure S21b), evidencing the rupture of sacrificial reversible hydrogen and coordination bonds during deformation. The maximum stress in the twentieth cycle (0.31 MPa) maintained approximately 72% of that in the first cycle (0.43 MPa), highlighting the structural integrity of the homogeneous network even with partial cross‐linking damage, thus effectively preventing material failure caused by stress concentration under repeated deformation. The exceptional mechanical performance of SMNF‑Egel arises from the synergistic interplay between its biomimetic multiscale architecture and dynamic crosslinking, as schematically illustrated in Figure 4j. During deformation, the SMNF networks act as robust load‑bearing frameworks that effectively transmit and distribute applied stress, thereby enhancing the overall strength of the material. The excellent interfacial compatibility and strong bonding between the in‐situ‑formed SMNFs and the surrounding gel matrix are critical for efficient stress transfer and for preventing interfacial debonding. Moreover, fibril slippage and reorientation, together with the sequential dissociation of abundant dynamic hydrogen and coordination bonds at the fibril–matrix interface and within the gel network, dissipate substantial energy, imparting high toughness and ultrahigh stretchability. Upon unloading, the reversible nature of these dynamic bonds enables their reformation, restoring the network structure. Consequently, SMNF‑Egel exhibits rapid elastic recovery and long‑term fatigue resistance even under large deformations.

The effect of SMNF content on the conductivity of SMNF‐Egel was investigated using electrochemical impedance spectroscopy (EIS). As shown in Figure 4k, increasing SMNF content from 0% to 1% resulted in a progressive decrease in impedance. Correspondingly, the conductivity of SMNF‐Egel‐1 reached 1.51 S m^−1^, over 2.5 times higher than that of SMNF‐Egel‐0 (0.56 S m^−1^) (Figure 4l). The conductivity enhancement can be attributed to the synergistic contribution of the hierarchical SMNF network in facilitating charge transport (Figure S22). The in‐situ‐formed SMNF network constructs a robust, interwoven 3D micro/nanofibrillar scaffold that not only reinforces the gel but also provides efficient ion‐migration pathways [37]. Abundant polar groups (─OH, ─NH_2_, ─COOH) on the fibril surfaces strongly interact with Ch⁺ and Cl^−^ ions in the PDES via hydrogen bonding and electrostatic attraction, confining and organizing mobile ions along the fibrils and forming low‐energy‐barrier ion channels with markedly enhanced mobility compared with the bulk Egel [36]. These functional groups also act as interfacial mediators, chemically coordinating with Ga^3+^ and physically adsorbing onto EGaIn microdroplet surfaces, as confirmed by XPS and FTIR, thereby significantly reducing interfacial resistance at the SMNF/PDES and SMNF/EGaIn interfaces and ensuring efficient charge transfer [55]. Meanwhile, the micro/nanofibrillar network restricts the mobility and aggregation of EGaIn microdroplets, promoting their uniform dispersion (Figure 3f; Figure S14), minimizing conductive discontinuities, and establishing synergistic ion–electron transport pathways in which ions migrate along SMNF‐guided channels while electrons percolate through the EGaIn network [62]. As a result, the SMNF network functions as a multifunctional conductive bridge that simultaneously enhances ionic and electronic transport, enabling the conductivity of SMNF‐Egel to surpass that of most reported eutectogels, including synthetic polymer‐based and natural polymer‐reinforced systems. (Figure 4m; Table S3).

The environmental stability and self‐healing ability of eutectogels are critical for practical applications, as they significantly improve material durability under extreme conditions (e.g., dryness, low temperature) and enable autonomous repair to maintain functional integrity, thus ensuring the long‐term reliability of flexible electronic devices [63]. Accordingly, we systematically evaluated the anti‐drying, anti‐freezing, and self‐healing properties of SMNF‐Egel through mechanical and electrical measurements. Additional results and discussion are provided in Figures S23–S25 and Video S3.

### 2.5 Strain Sensing and Application Demonstrations of SMNF‐Egel

**FIGURE 5:**
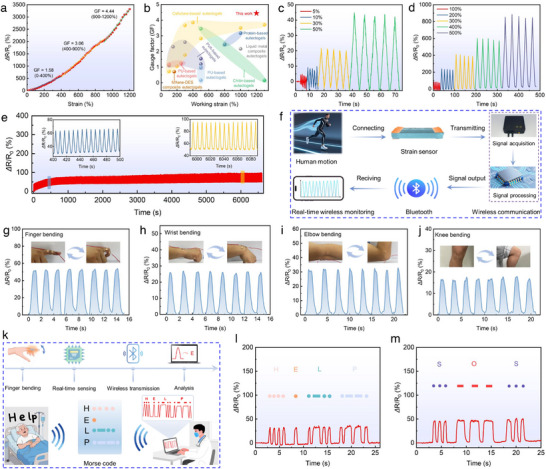
Strain sensing performance and application demonstration of SMNF‐Egel. (a) Relative resistance change (*ΔR/R_0_*) of SMNF‐Egel as a function of strain and the corresponding GF. (b) Comparison of the gauge factor and maximum working strain with previously reported eutectogels. The *ΔR/R_0_* of SMNF‐Egel at (c) small strain (5%–50%) and (d) large strain (100%–500%). (e) Durability test at 100% strain for 1000 cycles. (f) Schematic illustration of a wireless motion monitoring system utilizing the SMNF‐Egel sensor. Real‐time monitoring of (g) finger, (h) wrist, (i) elbow, and (j) knee joint movements using the wireless system. (k) Schematic illustration of potential Morse code translation applications based on the SMNF‐Egel sensor. Key messages such as (l) “HELP” and (m) “SOS” were transmitted via finger bending with coded rhythms.

Given its exceptional tensile strength, robustness, electrical conductivity, and skin‐compatible elastic modulus, SMNF‐Egel‐1 was chosen as the primary material for the development of sensors aimed at strain detection and physiological electrical signal monitoring, unless explicitly stated otherwise. As shown in Figure 5a, the relative resistance change (*ΔR/R_0_*) of SMNF‐Egel increased progressively with tensile strain, reaching 3316% at 1200% strain, indicating high sensitivity and a broad sensing range. Video S4 further demonstrates the electrical response of SMNF‐Egel during deformation; the brightness of an integrated LED decreased upon stretching and recovered after release. The gauge factor (GF), calculated from the linear fit of the *ΔR/R_0_*–strain curve, was 1.58 (0%–400%), 3.06 (400%–900%), and 4.44 (900%–1200%), confirming both high sensitivity and a wide dynamic range. It is worth noting that the SMNF‐Egel strain sensor outperforms most eutectogel‐based strain sensors in both working strain range and gauge factor (Figure 5b; Table S4). As presented in Figure 5c,d, SMNF‐Egel exhibited repeatable and stable responses under both small (5%–50%) and large (100%–500%) strains, with *ΔR/R_0_* increasing as the strain increased, verifying reliable sensing performance. Notably, *ΔR/R_0_* remained stable at different stretching rates (Figure S26), demonstrating rate‐independent strain sensing, which is advantageous for detecting diverse human motions [30]. The response and recovery times at 100% strain were 404 and 384 ms, respectively (Figure S27), ensuring accurate, real‐time detection. Additionally, during 1000 stretch–release cycles at 100% strain, SMNF‐Egel maintained stable *ΔR/R_0_* signals with only slight baseline drift due to mechanical hysteresis (Figure 5e), indicating excellent long‐term durability.

Owing to its superior mechanical compliance, high sensitivity, rapid response, and broad sensing range, the SMNF‐Egel strain sensor is highly promising for human motion monitoring. A wearable wireless motion monitoring system was developed using the SMNF‐Egel sensor to demonstrate its practical utility. As illustrated in Figure 5f, this system integrates real‐time signal acquisition, processing, encoding, Bluetooth transmission, and mobile app‐based monitoring. The system enabled real‐time detection and wireless transmission of strain signals, validating its feasibility in wireless motion sensing. As shown in Figure 5g–j and Video S5, diverse human motions, including finger, wrist, elbow, and knee bending, were dynamically tracked.

Beyond signal detection, efficient signal transmission is essential for real‐world applications [64]. As a proof of concept, a Morse code translation system based on SMNF‐Egel was developed for medical communication. As shown in Figure 5k, the sensor was placed near the finger joint to detect bending‐induced electrical signals, which were then processed and wirelessly transmitted via Bluetooth for analysis. Figure S28 presents the Morse code representations for the alphabet; a rapid 90° bend represents a “dot,” while a sustained 90° bend for 2 s indicates a “dash”. By combining these patterns, predefined waveforms corresponding to letters and phrases, such as “HELP” and “SOS,” can be generated through rhythmic finger gestures (Figure 5l,m; Figure S29), enabling silent remote communication for patients with limited mobility. The wireless design reduces caregiver dependence, healthcare costs, and workload, demonstrating significant potential for smart medical systems. These findings underscore the practical and versatile applications of SMNF‐Egel sensors in motion monitoring and data transmission.

### 2.6 Electrophysiological Signal Monitoring

Electrophysiological signals are fundamental for regulating human physiological functions and diagnosing tissue and organ health, playing a vital role in personal health management [65]. To explore this potential, we developed biomimetic SMNF‐Egel as a wearable bioelectrode for precisely monitoring electromyogram (EMG) and electrocardiogram (ECG) signals. Figure S30 shows that biological electrical signals originating from action potentials (AP) in excitable cells convert into extracellular potentials and local field potentials (*V*_AP_) [66]. These are transformed into gel potential (*V*_gel_) through faradaic or capacitive charge transfer, subsequently generating ionic currents within the gel, which are finally captured as voltage signals (*V*_record_) by electronic circuits [67]. Our results confirm that SMNF‐Egel exhibits skin‐matching softness, excellent stretchability, and high conductivity, ensuring stable adhesion and efficient electrical transmission. However, the signal transmission mechanism shown in Figure S30 indicates that high‐quality electrophysiological signal acquisition depends not only on mechanical matching and overall conductivity but also requires the gel to possess strong adhesion to ensure conformal contact and low interface impedance to enhance ion‐electron conversion efficiency [65, 68].

**FIGURE 6:**
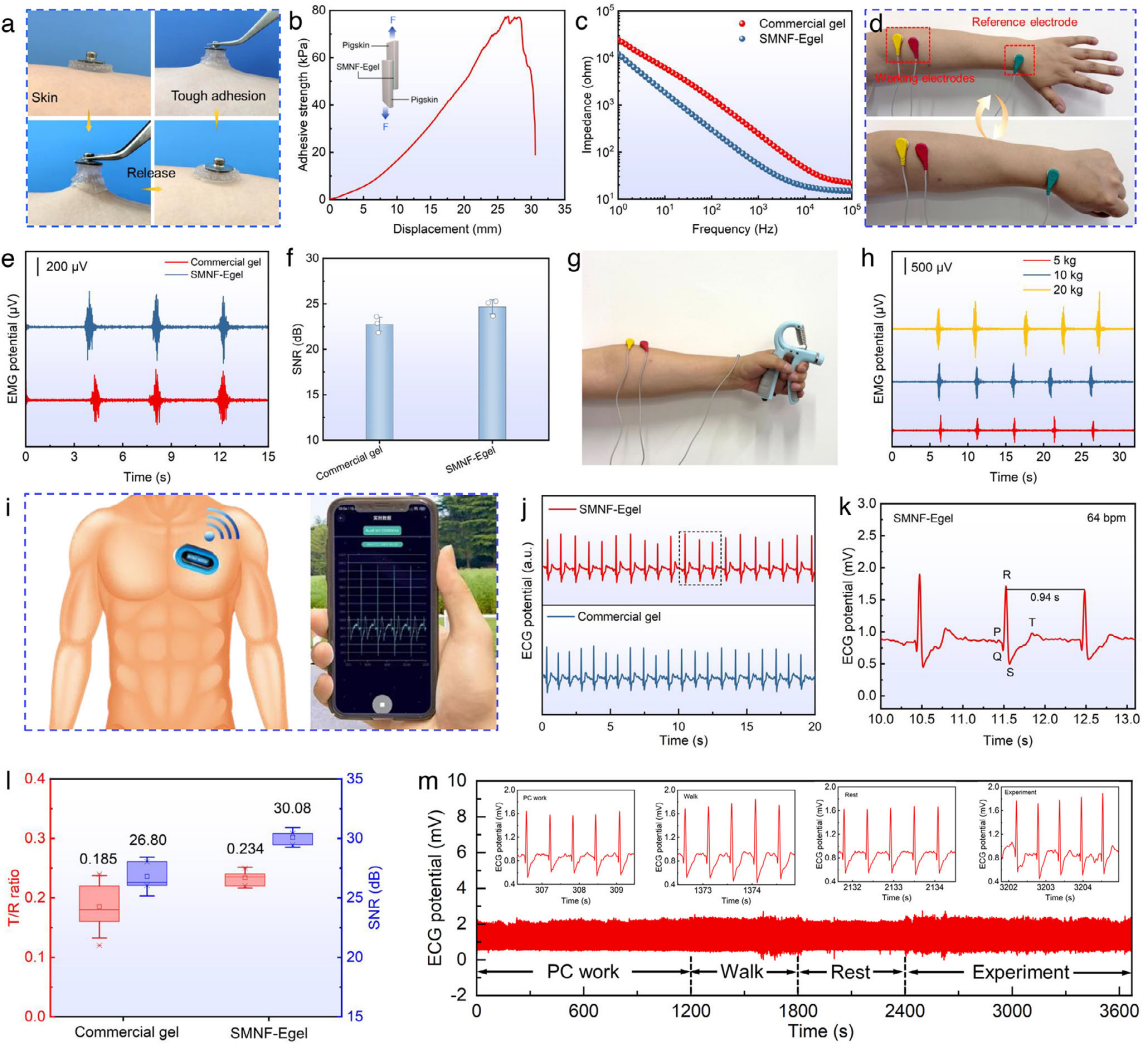
SMNF‐Egel as epidermal bioelectrodes for monitoring electrophysiological signals. (a) Photographs of SMNF‐Egel bioelectrodes attached to the skin before and after stretching. (b) Adhesive strength of SMNF‐Egel on pigskin. (c) Impedance comparison between SMNF‐Egel and commercial gel electrodes. (d) Photographs of EMG recording during fist clenching. (e) EMG signals and (f) signal‐to‐noise ratio (SNR) obtained by SMNF‐Egel and commercial gel during fist clenching. (g) Photograph of EMG monitoring during grip dynamometer testing. (h) EMG signals captured by SMNF‐Egel under grip forces of 5, 10, and 20 kg. (i) Schematic illustration of the wireless ECG measurement setup. (j) ECG signals recorded by SMNF‐Egel and commercial gel. (k) Enlarged ECG waveform highlighting PQRST complexes. (l) Comparison of T/R ratios and SNR values for ECG signals from SMNF‐Egel and commercial gel. (m) Continuous ECG monitoring using SMNF‐Egel during various activities, including computer work, walking, resting, and laboratory experiments. Data in (f) and (l) are presented as mean ± SD, n = 3.

To enhance performance, SMNF‐Egel was integrated with metal electrode buttons to produce epidermal bioelectrodes. As shown in Figure 6a, these electrodes adhere tightly under external force and recover quickly, preventing skin damage and detachment caused by device weight. Adhesive strength measured on pigskin via lap shear test (Figure 6b) was 77.5 kPa, indicating robust conformal contact. As evidenced in Figure 6c, SMNF‐Egel shows significantly lower interface resistance (299 Ω at 100 Hz) than commercial gels (1362 Ω), improving signal fidelity. For EMG monitoring, electrodes placed on forearm muscles and wrist successfully recorded muscle activity during repeated fist clenching (Figure 6d,e; Video S6), with a high SNR of 24.7 dB—superior to commercial gels (22.5 dB, Figure 6f). EMG amplitude increased proportionally with grip strength (5, 10, 20 kg), demonstrating quantitative assessment capability (Figure 6g,h; Figure S31).

Beyond EMG, ECG signals captured with SMNF‐Egel on a volunteer's chest displayed clear P, Q, R, S, and T waves (Figure 6i–k; Figure S32), enabling accurate cardiac diagnosis [69]. The ECG's SNR (30.08) was notably higher than commercial gels (26.80), and the T/R ratio (0.234) fell within clinical standards [70], indicating high fidelity (Figure 6l). The system facilitated stable, long‐term ECG monitoring during daily activities such as PC work, walking, and resting (Figure 6m; Video S7), with heart rates averaging within normal ranges (e.g., 94 bpm during PC work, 115 bpm during walking). These results demonstrate that SMNF‐Egel's exceptional toughness, adhesion, and conductivity support reliable, continuous monitoring in dynamic environments, highlighting its potential for long‐term, non‐invasive health assessment.

## 3 Conclusion

In summary, we have developed a hierarchical eutectogel (SMNF‐Egel) using a ChCl/AA PDES and EGaIn microdroplet‐catalyzed in situ polymerization, enabling the disassembly and reassembly of silk fibroin micro/nanofibers within the gel matrix. This strategy eliminates the need for external initiators or UV irradiation, greatly simplifying fabrication. The resulting SMNF‐Egel incorporated ECM‐inspired multiscale architectures, where molecular‐level dynamic bonds synergized with micro‐nanofibril reinforcement, exhibit outstanding mechanical strength (1.25 MPa), high toughness (23.09 MJ m^−3^), and ultra‐high elongation at break (2289%), while maintaining a skin‐matched modulus, superior conductivity (1.51 S m^−1^), and strong adhesion. These multifunctional properties enabled reliable wearable strain sensing, wireless Morse code communication, and high‐fidelity electrophysiological signal monitoring with low interfacial impedance. Although this work advances the structural and functional design of silk‑based eutectogels and establishes a versatile platform for next‑generation flexible and biointegrated electronic devices, several limitations warrant future attention. First, the long‑term in vivo biocompatibility has not been systematically assessed, and comprehensive studies on cytotoxicity, inflammatory responses, and in vivo degradation are required. Second, while the current fabrication process is well‐suited for laboratory‑scale preparation, improvements in material uniformity and cost efficiency are essential for large‑scale manufacturing. Addressing these challenges will further promote the practical translation of this technology.

## 4 Experimental Section

### 4.1 Materials

The raw silk fibers were provided by Qingyang Sanfang Silk Co., Ltd., China. Choline chloride (ChCl, ≥ 98%), acrylic acid (AA, ≥ 99%), eutectic gallium indium (EGaIn, Gallium 75.5 wt.% and Indium 24.5 wt.%), and anhydrous sodium carbonate (Na_2_CO_3_, ≥ 99.5%) were purchased from Aladdin Biochemical Technology Co., Ltd. (Shanghai, China). Methyl orange (MO), rhodamine B (AA, ≥ 99%), and Ga^3+^ standard solution were obtained from Macklin Biochemical Technology Co., Ltd. (Shanghai, China). All of the chemicals were used without further purification.

### 4.2 In Situ Deconstruction of SF Fibers by PDES

First, 10 g raw silk fibers were degummed in 500 mL Na_2_CO_3_ aqueous solution (0.5% w/w) at 100°C for 30 min. The degumming step was repeated once again to ensure the complete removal of sericin. The resulting SF fibers were thoroughly washed with deionized water and then dried overnight at 40°C. Then, 25 g ChCl and 25.7 g AA (mole ratio: 1:2) were heated in a sealed conical flask at 90°C for 30 min to prepare clear and transparent PDES. Finally, 0.5 g SF fibers were cut into pieces and added to PDES at 100°C with continuous magnetic stirring for 5 h, causing in situ deconstruction of the fibers to form a homogeneous SMNF‐PDES mixture.

### 4.3 Synthesis of SMNF‐Egel by EGaIn‐Induced Polymerization

A total of 0.7 g EGaIn was first added to 14 g AA, and then sonicated for 10 min (1 cycle = 3 s containing 1 pause) at 400 W with a probe ultrasonicator (JY98‐IIIDN, Scientz Co. Ltd.) under the ice‐water bath. Subsequently, 3.6 g EGaIn‐AA microdroplets were mixed with 14 g SMNF‐PDES. After stirring rapidly for 2 min, the homogeneous mixture was poured into a PTFE mold and incubated at room temperature to prepare SMNF‐reconstructed eutectogels (SMNF‐Egel). The obtained eutectogel was designated as SMNF‐PDES‐x, where x represented the mass fraction of SF fibers to PDES.

### 4.4 Characterization

The morphology was observed using SU8600 scanning electron microscopy (SEM, Hitachi, Japan) and Vhx‐970f ultra‐depth of field microscope (Keyence, Japan). The X‐ray diffraction (XRD) patterns of SF fibers and SMNF in the diffraction angle range from 5° to 45° were obtained on the SmartLab SE X‐ray diffractometer (Rigaku, Japan). The confocal images of SMNF‐Egel were obtained using an LSM900 laser scanning confocal microscope (LSCM, Zeiss, Germany) with an excitation wavelength of 561 nm. Rhodamine B was dissolved in PDES to enable it to show red fluorescence under laser irradiation, while the SMNF, without fluorescence, presented black. The temperature variation of the system during gelation was monitored with an A308 infrared thermal imager (FLIR, USA). The electron paramagnetic resonance (EPR) spectrum was applied to detect free radicals in the EGaIn‐SMNF‐PDES mixture for 5 min. 5, 5‐dimethyl‐1‐pyrroline‐N‐oxide (DMPO) was added to the above mixture as the free radical capture agent. The chemical structures of SF fiber and SMNF, and SMNF‐Egel were characterized with the Nicolet iS50 Fourier transform infrared spectrometer (FTIR, Thermo Fisher, USA) within the range of 500–4000 cm^−1^. ^1^H NMR spectra were recorded utilizing a Bruker 400 MHz nuclear magnetic resonance spectrometer. Deuteroxide (D_2_O) was used as an external reference. The X‐ray photoelectron spectra (XPS) were measured using the K‐Alpha photoelectron spectrometer (Thermo Scientific, USA). The viscosity of SMNF‐PDES, SMNF‐PDES‐Ga^3+^, and SMNF‐PDES‐EGaIn mixture was tested using an RST rheometer (Brookfield, USA).

### 4.5 Statistical Analysis

The dataset was preprocessed, including transformation and normalization. Data are shown as the mean ± standard deviation (SD). The error bars in the various data graphs represent SDs for three tests on each sample, which represent SDs for tests on three samples. All data were processed using Origin 2021.

### 4.6 Statement

The human subject experiments involved in this study (including human motion monitoring, EMG, and ECG signal acquisition) were approved by the Academic Committee of Anhui Polytechnic University, with the approval No. 20251116. All participants provided written informed consent prior to the experiments.

## Conflicts of Interest

The authors declare no conflicts of interest.

## Supporting information




**Supporting File 1**: advs73552‐sup‐0001‐SuppMat.docx.

**Supporting File 2**: advs73552‐sup‐0002‐VideoS1.mp4.

**Supporting File 3**: advs73552‐sup‐0003‐VideoS2.mp4.

**Supporting File 4**: advs73552‐sup‐0004‐VideoS3.mp4.

**Supporting File 5**: advs73552‐sup‐0005‐VideoS4.mp4.

**Supporting File 6**: advs73552‐sup‐0006‐VideoS5.mp4.

**Supporting File 7**: advs73552‐sup‐0007‐VideoS6.mp4.

**Supporting File 8**: advs73552‐sup‐0008‐VideoS7.mp4.

## Data Availability

The data that support the findings of this study are available from the corresponding author upon reasonable request.

## References

[1] H. J. Kim , J. H. Koo , S. Lee , T. Hyeon , and D. H. Kim , “Materials Design and Integration Strategies for Soft Bioelectronics in Digital Healthcare,” Nature Reviews Materials 10 (2025): 654, 10.1038/s41578-025-00819-w.

[2] Y. Li , N. N. Bai , Y. Chang , et al., “Flexible Iontronic Sensing,” Chemical Society Reviews 54 (2025): 4651. 40165624 10.1039/d4cs00870g

[3] Y. F. He , Y. Cheng , C. H. Yang , and C. F. Guo , “Creep‐Free Polyelectrolyte Elastomer for Drift‐Free Iontronic Sensing,” Nature Materials 23 (2024): 1107, 10.1038/s41563-024-01848-6. 38514845

[4] H. Zhang , W. Jia , M. Sun , et al., “High‐Strength and Fracture‐Resistant Ionogels via Solvent‐Tailored Interphase Cohesion in Nanofibrous Composite Networks,” Science Advances 11 (2025): aea6883, 10.1126/sciadv.aea6883. PMC12629177 41259524

[5] S. Ijaz , J. Wan , N. Ijaz , et al., “Advanced Conductive Eutectogel Material for Flexible Sensor Applications,” Advances in Colloid and Interface Science 344 (2025): 103610, 10.1016/j.cis.2025.103610. 40700849

[6] Y. Liu , R. Omar , G. Li , et al., “Adaptable Conductive Hydrogel‐Enabled Soft Electronics,” Progress in Materials Science 157 (2026): 101590.

[7] X. T. Fan , S. Q. Liu , Z. H. Jia , et al., “Ionogels: Recent Advances in Design, Material Properties and Emerging Biomedical Applications,” Chemical Society Reviews 52 (2023): 2497, 10.1039/D2CS00652A. 36928878

[8] T. Liu , Q. A. Wu , H. S. Liu , et al., “A Crosslinked Eutectogel for Ultrasensitive Pressure and Temperature Monitoring From Nostril Airflow,” Nature Communications 16 (2025): 3334, 10.1038/s41467-025-58631-7. PMC11978763 40199936

[9] Y. Hu , W. H. Yang , J. M. Zhan , C. Y. Pu , L. F. Zhong , and H. H. Hou , “Eutectogels: Recent Advances and Emerging Biological Applications,” Advanced Functional Materials 35 (2025): 2425778, 10.1002/adfm.202425778.

[10] S. Q. Sun , L. Yu , J. C. Teng , et al., “Eutectic Gels: Presentation and Prospect,” Applied Materials Today 39 (2024): 102342.

[11] N. Tang , Y. Jiang , H. Zhang , and J. Hu , “Innovative Eutectogels: Designs, Principles, and Applications,” Nano Research (2025), 10.26599/NR.2025.94907847.

[12] J. Wang , S. Zhang , Z. Ma , and L. Yan , “Deep Eutectic Solvents Eutectogels: Progress and Challenges,” Green Chemical Engineering 2 (2021): 359, 10.1016/j.gce.2021.06.001.

[13] R. Hu , D. W. Sun , and Y. Tian , “Emerging Eutectogel Materials: Development, Synthesis, Properties, And Applications In Food Science,” Trends Food Science Technology 159 (2025): 104962.

[14] G. Liu , D. Wang , H. Li , et al., “Energy Dissipation Mediated by Multiple Noncovalent Interactions in Hydrogen‐Bonded Organic Frameworks‐Based Hydrogels for Wearable Gesture‐to‐Recognition Translation,” Angewandte Chemie 137 (2025): 202514750, 10.1002/ange.202514750. 40904203

[15] R. Zhou , Y. Jin , W. H. Zeng , et al., “Versatile Quasi‐Solid Ionic Conductive Elastomer Inspired by Desertification Control Strategy for Soft Iontronics,” Advanced Functional Materials 33 (2023): 2301921, 10.1002/adfm.202301921.

[16] C. W. Lu , X. Y. Wang , Y. Shen , et al., “Skin‐Like Transparent, High Resilience, Low Hysteresis, Fatigue‐Resistant Cellulose‐Based Eutectogel for Self‐Powered E‐Skin and Human–Machine Interaction,” Advanced Functional Materials 34 (2024): 2311502, 10.1002/adfm.202311502.

[17] X. K. Li , J. Z. Liu , Q. Q. Guo , X. X. Zhang , and M. Tian , “Polymerizable Deep Eutectic Solvent‐Based Skin‐Like Elastomers With Dynamic Schemochrome and Self‐Healing Ability,” Small 18 (2022): 2201012, 10.1002/smll.202201012. 35403800

[18] X. R. Zhang , Q. J. Fu , Y. C. Wang , et al., “Tough Liquid‐Free Ionic Conductive Elastomers With Robust Adhesion and Self‐Healing Properties for Ionotronic Devices,” Advanced Functional Materials 34 (2024): 2307400, 10.1002/adfm.202307400.

[19] S. C. Sun , S. W. Hao , Y. Q. Liu , et al., “Mechanically Resilient, Self‐Healing, and Environmentally Adaptable Eutectogel‐Based Triboelectric Nanogenerators for All‐Weather Energy Harvesting and Human–Machine Interaction,” ACS Nano 19 (2024): 811, 10.1021/acsnano.4c12130. 39700480

[20] C. A. W. Lu , X. Y. Wang , Y. Shen , et al., “Liquid‐Free, Anti‐Freezing, Solvent‐Resistant, Cellulose‐Derived Ionic Conductive Elastomer for Stretchable Wearable Electronics and Triboelectric Nanogenerators,” Advanced Functional Materials 32 (2022): 2207714, 10.1002/adfm.202207714.

[21] J. L. Zhao , X. C. Wang , L. Lin , et al., “High‐Strength and High‐Stretchability All‐Solid‐State Double‐Network Ion‐conductive Elastomers Based on Supramolecular Deep Eutectic Polymer,” Advanced Functional Materials 35 (2025): 2500590, 10.1002/adfm.202500590.

[22] Q. Quan , C. L. Fan , N. A. Pan , et al., “Tough and Stretchable Phenolic‐Reinforced Double Network Deep Eutectic Solvent gels for Multifunctional Sensors With Environmental Adaptability,” Advanced Functional Materials 33 (2023): 2303381, 10.1002/adfm.202303381.

[23] X. M. Wang , L. Weng , X. R. Zhang , Z. J. Wu , L. Z. Guan , and X. Li , “A Self‐Healing Conductive Elastomer Based on a Polymerizable Deep Eutectic Solvent,” Small 20 (2024): 2304828, 10.1002/smll.202304828. 37939295

[24] Y. F. Lan , W. W. Liu , Z. M. Lv , et al., “Liquid‐Free, Tough and Transparent Ionic Conductive Elastomers Based on Nanocellulose for Multi‐Functional Sensors and Triboelectric Nanogenerators,” Nano Energy 129 (2024): 110047, 10.1016/j.nanoen.2024.110047.

[25] Y. M. Yan , W. J. Deng , D. Xie , and J. Hu , “Silk Fibroin Hydrogel for Pulse Waveform Precise and Continuous Perception,” Advanced Healthcare Materials 14 (2025): 2403637, 10.1002/adhm.202403637. 39707661

[26] L. Shu , X. F. Zhang , J. Y. Miu , and J. F. Yao , “Robust and Highly Conductive Cellulose‐Based Eutectogel for Flexible Electronics,” ACS Applied Polymer Materials 6 (2024): 13785, 10.1021/acsapm.4c02632.

[27] Q. Gao , F. Q. Sun , Y. Li , et al., “Biological Tissue‐Inspired Ultrasoft, Ultrathin, and Mechanically Enhanced Microfiber Composite Hydrogel for Flexible Bioelectronics,” Nano‐Micro Letters 15 (2023): 139. 37245163 10.1007/s40820-023-01096-4 PMC10225432

[28] L. Rijns , M. B. Baker , and P. Y. W. Dankers , “Using Chemistry To Recreate the Complexity of the Extracellular Matrix: Guidelines for Supramolecular Hydrogel–Cell Interactions,” Journal of the American Chemical Society 146 (2024): 17539, 10.1021/jacs.4c02980. 38888174 PMC11229007

[29] A. M. Rosales and K. S. Anseth , “The Design of Reversible Hydrogels to Capture Extracellular Matrix Dynamics,” Nature Reviews Materials 1 (2016): 15012, 10.1038/natrevmats.2015.12. PMC5714327 29214058

[30] X. Sun , Y. L. Zhu , J. Y. Zhu , K. Le , P. Servati , and F. Jiang , “Tough and Ultrastretchable Liquid‐Free Ion Conductor Strengthened by Deep Eutectic Solvent Hydrolyzed Cellulose Microfibers,” Advanced Functional Materials 32 (2022): 2202533, 10.1002/adfm.202202533.

[31] X. M. Li , L. N. Xu , J. L. Gao , M. Q. Yan , and Q. Y. Wang , “Highly Stretchable, Tough, and Transparent Chitin Nanofiber‐Reinforced Multifunctional Eutectogels for Self‐Powered Wearable Sensors,” ACS Sensors 10 (2025): 886, 10.1021/acssensors.4c02535. 39936831

[32] Y. Zhang , Y. F. Wang , Y. Guan , and Y. J. Zhang , “Peptide‐Enhanced Tough, Resilient and Adhesive Eutectogels for Highly Reliable Strain/Pressure Sensing under Extreme Conditions,” Nature Communications 13 (2022): 6671, 10.1038/s41467-022-34522-z. PMC9637226 36335147

[33] S. Wang , X. S. Du , X. Cheng , Z. L. Du , Z. Y. Zhang , and H. B. Wang , “Ultrahigh Stretchable, Highly Transparent, Self‐Adhesive, and Environment‐Tolerant Chitin Nanocrystals Engineered Eutectogels Toward Multisignal Sensors,” ACS Applied Materials & Interfaces 16 (2024): 45537, 10.1021/acsami.4c09589. 39138982

[34] B. Y. Guo , M. M. Yao , S. Chen , et al., “Environment‐Tolerant Conductive Eutectogels for Multifunctional Sensing,” Advanced Functional Materials 34 (2024): 2315656, 10.1002/adfm.202315656.

[35] H. Zhang , N. Tang , X. Yu , M. H. Li , and J. Hu , “Strong and Tough Physical Eutectogels Regulated by the Spatiotemporal Expression of Non‐Covalent Interactions,” Advanced Functional Materials 32 (2022): 2206305, 10.1002/adfm.202206305.

[36] K. Y. Cao , Y. Zhu , Z. H. Zheng , et al., “Bio‐Inspired Multiscale Design for Strong and Tough Biological Ionogels,” Advanced Science 10 (2023): 2207233. 36905237 10.1002/advs.202207233 PMC10161113

[37] G. Y. Jiang , G. Wang , Y. Zhu , et al., “A Scalable Bacterial Cellulose Ionogel for Multisensory Electronic Skin,” Research 2022 (2022): 9814767. 35711672 10.34133/2022/9814767 PMC9188022

[38] K. Yang , J. Zhang , C. Zhang , J. Guan , S. Ling , and Z. Shao , “Hierarchical Design of Silkworm Silk for Functional Composites,” Chemical Society Reviews 54 (2025): 4973, 10.1039/D4CS00776J. 40237181

[39] S. J. Ling , D. L. Kaplan , and M. J. Buehler , “Nanofibrils In Nature And Materials Engineering,” Nature Reviews Materials 3 (2018): 18016, 10.1038/natrevmats.2018.16. PMC8221570 34168896

[40] C. M. Li , J. Q. Wu , H. Y. Shi , et al., “Fiber‐Based Biopolymer Processing As s Route Toward Sustainability,” Advanced Materials 34 (2022): 2105196. 10.1002/adma.202105196 PMC8741650 34647374

[41] Q. Wang , S. J. Ling , Q. Z. Yao , et al., “Observations of 3 Nm Silk Nanofibrils Exfoliated From Natural Silkworm Silk Fibers,” ACS Materials Letters 2 (2020): 153, 10.1021/acsmaterialslett.9b00461.

[42] F. G. Omenetto and D. L. Kaplan , “New Opportunities for an Ancient Material,” Science 329 (2010): 528, 10.1126/science.1188936. 20671180 PMC3136811

[43] H. W. Yang , P. Wang , Q. L. Yang , et al., “Superelastic and Multifunctional Fibroin Aerogels From Multiscale Silk Micro‐Nanofibrils Exfoliated via Deep Eutectic Solvent,” International Journal of Biological Macromolecules 224 (2023): 1412, 10.1016/j.ijbiomac.2022.10.228. 36550790

[44] Y. Wang , Z. H. Yang , B. Z. Jia , et al., “Natural Deep Eutectic Solvent‐Assisted Construction of Silk Nanofibrils/Boron Nitride Nanosheets Membranes With Enhanced Heat‐Dissipating Efficiency,” Advanced Science 11 (2024): 2403724, 10.1002/advs.202403724. 39054638 PMC11529046

[45] Q. W. Lu , H. F. Li , and Z. J. Tan , “Natural Cellulose Reinforced Multifunctional Eutectogels for Wearable Sensors and Epidermal Electrodes,” Carbohydrate Polymers 348 (2025): 122939, 10.1016/j.carbpol.2024.122939. 39567155

[46] B. C. Cheng , Z. Y. Lei , and P. Y. Wu , “Bio‐Derived Crystalline Silk Nanosheets for Versatile Macroscopic Assemblies,” Nano Research 15 (2022): 5538, 10.1007/s12274-022-4124-x.

[47] Z. A. Hu , S. Q. Yan , X. F. Li , R. C. A. You , Q. Zhang , and D. L. Kaplan , “Natural Silk Nanofibril Aerogels With Distinctive Filtration Capacity and Heat‐Retention Performance,” ACS Nano 15 (2021): 8171, 10.1021/acsnano.1c00346. 33848124

[48] H. W. Yang , L. Cheng , Q. L. Yang , et al., “Multifunctional and Durable Thermal Management Coating From Sericin‐Mxene Biohybrid on Silk Fabric Micro‐Etched by Deep Eutectic Solvent,” Applied Surface Science 623 (2023): 156962, 10.1016/j.apsusc.2023.156962.

[49] S. Ghosh , F. Shajahan , J. Adhikari , A. K. Bera , A. Ghosh , and F. Pati , “Visible Light Cross‐Linked Methacrylated Silk Fibroin Enables Enhanced Osteogenic Response in Bioprinted Dual‐Layer Guided Bone Regeneration Membrane,” ACS Applied Materials & Interfaces 17 (2025): 23553, 10.1021/acsami.4c22349. 40222015

[50] G. Chen , N. Matsuhisa , Z. Y. Liu , et al., “Plasticizing Silk Protein for On‐Skin Stretchable Electrodes,” Advanced Materials 30 (2018): 1800129, 10.1002/adma.201800129. 29603437

[51] D. L. Barreiro , Z. Martín‐Moldes , A. B. Fernández , V. Fitzpatrick , D. L. Kaplan , and M. J. Buehler , “Molecular Simulations Of The Interfacial Properties In Silk–Hydroxyapatite Composites,” Nanoscale 14 (2022): 10929, 10.1039/D2NR01989B. 35852800 PMC9351605

[52] J. Y. Yang , Y. Yan , L. Z. Huang , et al., “Conductive Eutectogels Fabricated by Dialdehyde Xylan/Liquid Metal‐Initiated Rapid Polymerization for Multi‐Response Sensors and Self‐Powered Applications,” ACS Nano 19 (2025): 2171, 10.1021/acsnano.4c11127. 39791699

[53] G. Shi , X. W. Peng , J. M. Zeng , et al., “A Liquid Metal Microdroplets Initialized Hemicellulose Composite for 3D Printing Anode Host in Zn‐Ion Battery,” Advanced Materials 35 (2023): 2300109. 10.1002/adma.202300109 37009654

[54] M. Wang , Z. B. Lai , X. L. Jin , T. L. Sun , H. C. Liu , and H. S. Qi , “Multifunctional Liquid‐Free Ionic Conductive Elastomer Fabricated by Liquid Metal Induced Polymerization,” Advanced Functional Materials 31 (2021): 2101957, 10.1002/adfm.202101957.

[55] X. L. Yan , Z. L. Liu , Y. B. Fu , et al., “Liquid Metal@Silk Fibroin Peptide Particles Initiated Hydrogels With High Toughness, Adhesion, and Conductivity for Portable and Continuous Electrophysiological Monitoring,” Advanced Functional Materials 35 (2025): 2420240, 10.1002/adfm.202420240.

[56] J. Ma , Y. L. Lin , Y. W. Kim , et al., “Liquid Metal Nanoparticles as Initiators for Radical Polymerization of Vinyl Monomers,” ACS Macro Letters 8 (2019): 1522, 10.1021/acsmacrolett.9b00783. 35651195

[57] S. A. Jaseem , P. Rahmani , T. Sakorikar , et al., “Liquid Metals as Initiators of Free‐Radical Polymerization of Hydrogels: A Perspective,” Advanced Functional Materials (2025): 14024, 10.1002/adfm.202514024.

[58] M. Q. You , J. Zhou , Y. M. Zao , et al., “Green Synthesis of Multifunctional Wood‐Based Eutectogels via Initiator‐Free Solar Polymerization,” Chemical Engineering Journal 504 (2025): 158902, 10.1016/j.cej.2024.158902.

[59] J. J. Wei , H. Chen , F. Pan , et al., “Reusable Liquid Metal‐Based Hierarchical Hydrogels With Multifunctional Sensing Capability for Electrophysiology Electrode Substitution,” ACS Nano 19 (2025): 15554, 10.1021/acsnano.4c16933. 40254826

[60] Y. Z. Shao , C. Dang , H. B. Qi , et al., “Polyfunctional Eutectogels with Multiple Hydrogen‐Bond‐Shielded Amorphous Networks for Soft Ionotronics,” Matter 7 (2024): 4076.

[61] H. Zhou , M. Yang , W. He , et al., “A Thermoresponsive Bioadhesive Mxene Hydrogel for Intelligent Brain‐Machine Interaction Sensing,” Matter 8 (2025): 102150.

[62] Y. H. Ye and F. Jiang , “Highly Stretchable, Durable, and Transient Conductive Hydrogel for Multi‐Functional Sensor and Signal Transmission Applications,” Nano Energy 99 (2022): 107374, 10.1016/j.nanoen.2022.107374.

[63] J. L. Wang , K. Zhao , Y. B. Zhao , and C. Q. Ye , “Highly Conductive, Ultratough, and Adhesive Eutectogels with Environmental Tolerance Enabled By Liquid Metal Composites,” Small 21 (2025): 2410806. 10.1002/smll.202410806 39822060

[64] W. W. Li , Z. Z. Chen , C. Xu , et al., “A Seamlessly Integrated Sandwich‐Structured Hydrogel for Supercapacitors and Multimodal Wearable Sensors Enabling Information Transmission,” Advanced Functional Materials 35 (2025): 12653, 10.1002/adfm.202512653.

[65] T. Li , H. B. Qi , C. C. Zhao , et al., “Robust Skin‐Integrated Conductive Biogel for High‐Fidelity Detection Under Mechanical Stress,” Nature Communications 16 (2025): 88, 10.1038/s41467-024-55417-1. PMC11695986 39747025

[66] M. Gong , X. B. Wang , Y. Wu , et al., “Jellyfish‐Inspired Ultrastretchable, Adhesive, Self‐Healing, and Photoswitchable Fluorescent Ionic Skin Enabled by a Supramolecular Zwitterionic Network,” Nano Letters 25 (2025): 6957, 10.1021/acs.nanolett.5c00441. 40241348

[67] M. Gong , X. B. Wang , H. An , et al., “Supramolecular Zwitterionic Network Enabling Environment‐Tolerant, Transparent, Adhesive, and Biocompatible Organogel for Epidermal Electronics,” ACS Macro Letters 14 (2025): 448. 40114356 10.1021/acsmacrolett.5c00098

[68] Z. M. Zhang , J. W. Yang , H. Y. Wang , et al., “A 10‐Micrometer‐Thick Nanomesh‐Reinforced Gas‐Permeable Hydrogel Skin Sensor for Long‐Term Electrophysiological Monitoring,” Science Advances 10 (2024): eadj5389. 38198560 10.1126/sciadv.adj5389 PMC10781413

[69] X. R. Yan , R. R. Zhao , H. J. Lin , Z. D. Zhao , S. S. Song , and Y. F. Wang , “Nucleobase‐Driven Wearable Ionogel Electronics for Long‐Term Human Motion Detection and Electrophysiological Signal Monitoring,” Advanced Functional Materials 35 (2025): 2412244, 10.1002/adfm.202412244.

[70] C. B. He , J. Zhang , H. F. Wang , et al., “Accurate and Reliable Detection of Dynamic Electrophysiological Signals via in‐situ Formation of Epidermal Electrodes,” Advanced Functional Materials (2025), 10.1002/adfm.202509372.

