# Supplementary material for: EGaIn‐Activated Bioinspired Silk Micro/Nanofibril Eutectogels Breaking the Strength–Conductivity Trade‐Off for High‐Performance Wearable Bioelectronics
Source: Adv Sci (Weinh). 2026 Jan 4;13(14):e20723. doi: 10.1002/advs.202520723 (PMC12970237; doi:10.1002/advs.202520723)
Supplement: Supplementary file 1 — Supporting File 1: advs73552‐sup‐0001‐SuppMat.docx. [file ADVS-13-e20723-s001.docx]

**Supporting Information**

**EGaIn-Activated Bioinspired Silk Micro/Nanofibril Eutectogels Breaking the Strength–Conductivity Trade-off for High-Performance Wearable Bioelectronics**

*Haiwei Yang, Dongdong Ye*, Yezi You, Ming Fu,* *Zongqian Wang**

H. Yang, Prof. Y. You

Department of Polymer Science and Engineering, University of Science and Technology of China, Hefei, Anhui 230026, China;

H. Yang, Prof. Z. Wang

School of Textile and Garment, Innovation Center for Anhui Ecological Textile Printing and Dyeing Manufacturing Industry, Anhui Polytechnic University, Wuhu, Anhui 241000, China;

E-mail: [wzqian@ahpu.edu.cn](mailto:wzqian@ahpu.edu.cn) (Z. Wang)

Prof. Z. Wang

Key Laboratory of Textile Fiber and Products (Ministry of Education), Wuhan Textile University, Wuhan 430200, China;

Prof. D. Ye

School of Materials and Chemistry, Anhui Agricultural University, Hefei, Anhui 230036, China;

E-mail: ydd@whu.edu.cn (D. Ye)

Prof. M. Fu

Hefei Institute for Public Safety Research, Tsinghua University, Hefei, Anhui 230601, China;

**This file includes:**

Supplementary Methods

Supplementary Figure S1-S32

Supplementary Table S1-S4

Supplementary References

**Other supplementary materials for this manuscript include the following:**

Supplementary Video 1-7

**Supplementary Methods**

***Characterization of ChCl/AA PDES:*** The FTIR spectrum of PDES was obtained using a Nicolet iS50 Fourier transform infrared spectrometer (Thermo Fisher Scientific, USA). The ^1^H NMR spectrum of PDES was recorded using a Bruker AV 500M nuclear magnetic resonance spectrometer. Deuterated chloroform was used as an external reference. The melting point of PDES was measured using a NETZSCH DSC200F3 differential scanning calorimeter (Netzsch, Germany) from -100 to 100 °C at a heating rate of 10 °C/min under nitrogen protection.

***Mechanical properties tests:*** The tensile mechanical properties of SMNF-Egel were measured using an EZ-LX universal testing machine (Shimazu, Japan) equipped with a 500 N load cell. SMNF-Egel samples were cut into rectangular specimens (length: ~ 50 mm, width: ~ 10 mm, thickness: ~ 2 mm) for tensile testing. The gauge length and tensile rate were set to 10 mm and 50 mm·min^-1^, respectively. The elastic modulus of the SMNF-Egel was obtained by calculating the slope of the stress-strain curve in the strain range of 10-30%. The toughness (*T*) of the SMNF-Egel was evaluated by calculating the area under the tensile stress-strain curve to the fracture point through the following equation. At least three tensile tests were performed for each eutectogel sample to obtain the average value.

$$T=\int_{\varepsilon_{0}}^{\varepsilon_{f}} \sigma\left( \varepsilon\right)d\varepsilon$$

where *ε_0_* and *ε_f_* correspond to the initial tensile point and fracture points on the stress-strain curve, respectively. In the cyclic loading-unloading test, the samples were first stretched to a preset strain, followed by release at the same rate. The dissipated energy (*U_dissipated_*) for each cycle is defined as the area of the hysteresis loop enclosed by the loading-unloading curve, which can be calculated according to the following integral equation.

$$U_{dissipated}= \int_{\varepsilon_{0}}^{\varepsilon_{max}} \sigma_{loading}(\varepsilon)d\varepsilon-\int_{\varepsilon_{0}}^{\varepsilon_{max}} \sigma_{unloading}(\varepsilon)d\varepsilon$$

where *ε_0_* and *ε_max_* represent the initial tensile strain and maximum tensile strain for each loading-unloading cycle, respectively.

The energy dissipation ratio (*η*) is an important indicator for evaluating the energy dissipation efficiency of gel materials during dynamic deformation, which can be calculated by the following equation.

$$\eta=\frac{U_{dissipated}}{U_{total}}$$

$$U_{total}=\int_{\varepsilon_{0}}^{\varepsilon_{max}} \sigma\left( \varepsilon\right)d\varepsilon$$

where *U_total_* is the total energy, representing the elastic energy stored in the gel material when it is stretched to a predetermined maximum strain (*ε_max_*).

***Conductivity measurement of SMNF-Egel:*** For conductivity testing, a regular SMNF-Egel (length: ~10 mm, width: ~10 mm, thickness: 2 mm) was sandwiched between two stainless steel electrodes. The resistance of the SMNF-Egel was measured by the electrochemical impedance spectroscopy using the CHI660E electrochemical workstation (Chenhua, Shanghai) from 10^0^ to 10^5^ Hz. The conductivity (*ρ*, S·m^-1^) of SMNF-Egel was calculated according to the following equation.

$$\rho=\frac{L}{R\times S}$$

where *L* is the distance between the two stainless steel electrodes, *S* is the cross-sectional area of SMNF-Egel, and *R* is the resistance of SMNF-Egel, obtained from the intercept of the AC impedance spectrum on the X-axis.

***Environmental stability:*** For comparison with the environmental stability of SMNF-Egel, the hydrogel reconstructed by SMNF (SMNF-hydrogel) was prepared as a control sample through the synergistic strategy of PDES in situ deconstruction and EGaIn-induced polymerization. Firstly, the SF fibers were cut into small pieces and immersed in ChCl/AA PDES (mass ratio: 1:100) at 100 °C with constant magnetic stirring for 5 h to obtain the paste-like mixture. Following this, the resultant paste-like mixture was washed with deionized water through the assistance of vacuum filtration to remove residual PDES and obtain the deconstructed SMNF. Subsequently, 0.14 g lyophilized SMNF was added to 14 g AA aqueous solution (the molar ratio of AA to water: 2:1) and stirred continuously for 30 min at room temperature to obtain a homogeneous SMNF-AA mixture. Finally, 3.6 g EGaIn-AA microdroplets were introduced into the mixture to initiate AA monomer polymerization to prepare the SMNF hydrogel.

The SMNF-Egel and SMNF-hydrogel were stored in the ambient environment for 30 days, and their relative mass changes were recorded to evaluate their anti-drying properties. After 30 days of storage, the mechanical properties of SMNF-Egel were also tested. Furthermore, the thermal stability of SMNF-Egel and SMNF-hydrogel was determined by using a Q50TA thermogravimetric analyzer (TA Instruments, Inc., USA) in the temperature range of 30-600 °C at a heating rate of 10 °C/min under a nitrogen atmosphere. The anti-freezing properties of SMNF-Egel and SMNF-hydrogel were evaluated using a DSC 200F3 differential scanning calorimeter (Netzsch, Germany). An appropriate amount of gel sample was placed in an aluminum pan and cooled from 20 to -80 °C at a rate of 10 °C·min^-1^. After being held at -80 °C for 5 min, the sample was heated to 100 °C at a rate of 10 °C·min^-1^, and finally cooled to 20°C at the same rate. The tensile properties of SMNF-Egel before and after being placed at -50 °C for 24 h were further tested using a universal testing machine (Shimadzu, Japan).

***Self-healing ability test:*** The SMNF-Egel was cut into half using a sharp blade and re-attached immediately. After being stored under ambient conditions for different periods (12, 24, 48, and 72 h), the tensile properties were tested by the EZ-LX universal testing machine. For electrical self-healing performance, the 01RC-resistance testing system (LinkZill, Hangzhou) was employed to measure the resistance changes of SMNF-Egel during the cutting and healing. The microscopic morphology of SMNF-Egel at different healing times was observed using the Vhx-970f ultra depth of field microscope (Keyence, Japan).

***Testing for the adhesive strength of SMNF-Egel to porcine skin:*** The adhesive strength of SMNF-EGel to porcine skin was tested using a lap shear testing method on a universal testing machine. The SMNF-EGel sample with 20 × 20 × 2 mm was placed between two pieces of porcine skin and compressed with a 50 g weight for 1 min. Subsequently, the two porcine skin samples were stretched to separation at a rate of 50 mm·min^-1^ under ambient conditions. The adhesive strength was calculated from the maximum load divided by the adhesion area.

***Strain sensing performance and application demonstration:*** The strain sensing performance of SMNF-Egel was investigated using an EZ-LX universal testing machine (Shimadzu, Japan) combined with the 01RC resistance testing system (LinkZill company, Hangzhou). The SMNF-Egel was fixed to a universal testing machine. Both ends of the SMNF-Egel were connected to a dynamic resistance testing system via wires, and its resistance was recorded during the stretching and recovery. The relative resistance change (*ΔR/R_0_*) and gauge factor (*GF*) were calculated by the following equations.

$$\frac{\Delta R}{R_{0}}=\frac{R-R_{0}}{R_{0}}\times100\%$$

$$GF=\frac{\Delta R}{R_{0}\times\varepsilon}$$

where *R_0_* is the resistance of the SMNF-Egel at the initial state, *R* is the real-time resistance of the SMNF-Egel during deformation, and *ε* is the applied strain.

To demonstrate the application of SMNF-Egel strain sensors in wireless monitoring of human motion, the SMNF-Egel with dimensions of 30 × 10 × 2 mm was attached to the corresponding body parts of a volunteer (such as fingers, wrists, elbows, and knees) using copper conductive tape. The strain sensing signals generated from human motions were acquired by a commercially portable resistance testing system (TruEbox, 01RC, LinkZill company) with a sampling rate of 50 Hz. The raw data were transmitted in real-time via Bluetooth to the Android application (TruEbox-01RC) for visualization, recording, and initial analysis. All subsequent signal processing and statistical analyses were based on this recorded raw data.

For the application demonstration of Morse code information transmission, the SMNF-Egel sensor was attached to the volunteer's finger joint using copper conductive tape and connected to a dynamic resistance testing system. The spike signals and square models generated by a 90° bend with an immediate recovery and a 90° bend held for 2 seconds before recovery represent the “dots” and “dashes” in Morse code sequence, respectively, for information transmission.

***Application in epidermal bioelectrodes:*** The circular SMNF-Egel (diameter: 15 mm, thickness: 2 mm) was integrated with metal electrode buttons to manufacture epidermal bioelectrodes for monitoring electromyographic (EMG) and electrocardiographic (ECG) signals. For EMG signal detection, according to the three-point method, the detection electrodes were attached to the forearm muscle bundle, with the reference electrode attached to the wrist. The EMG signals generated from clenching the fist and hand grip exercises were recorded by the SMNF-Egel epidermal bioelectrode combined with the wireless EMG Pro electromyography sensor module (Runyitaiyi Technology Co., Ltd.) with a sampling rate of 500 Hz. The raw data were transmitted in real-time via Bluetooth to the companion PC software (RunE_Module) for visualization, recording, and initial analysis. The signal-to-noise ratio (SNR) of EMG signals was calculated by the following equation^[1]^.

$$SNR=20\times lg(\frac{{RMS}_{signal}}{{RMS}_{noise}})$$

where *RMS_signal_* is the root mean square of the signal intensity, and *RMS_noise_* is the root mean square of the signal noise. For wireless ECG monitoring, two circular SMNF-Egel bioelectrodes were connected to a wireless ECG sensor module (Runyitaiyi Technology Co., Ltd.). The assembled testing module was attached to the left chest of volunteers to capture the ECG signals with a sampling rate of 250 Hz under the resting state. The raw data were transmitted in real-time via Bluetooth to the Android application (W1-Module) for visualization, recording, and initial analysis. Additionally, the ECG signals were continuously monitored in real time during different scenarios, including PC work, walking, resting, and conducting experiments. The SNR of ECG signals was defined as follows^[2, 3]^:

$$SNR=20\times lg(\frac{V_{signal}}{V_{noise}})$$

where the *V_signal_* was calculated as the average amplitude of ten consecutive R-waves, while the *V_noise_* was quantified as the root mean square of the signal amplitude in the 0.2 s window preceding the P-wave. For the calculation of the T/R value, ten P-QRS-T waveforms were randomly selected from the ECG signal, and the ratio of T and R wave peak values was used to characterize the sensitivity of the ECG signal^[4]^.

**Molecular dynamics simulation**

***Model construction of crystalline and amorphous domains of silk fibroin:*** The crystal structure of silk protein was obtained from the Protein Data Bank (PDB) file with the code 2SLK. The initial amorphous domain of silk fibroin was constructed using Avogadro software^[5]^by arranging 12 fully-extended peptides (amino acid sequence: TGSSGFGPYVANGGYSGYEYAWSSESDFGT) in a 4×3 array^[6, 7]^. These amorphous chains were arranged in an antiparallel manner, with an initial distance of 5 Å between adjacent chains. Preprocessing simulations of the initial amorphous domain were conducted using the GROMACS 2021.5 software package^[8]^. The simulated system was set up in a closed environment with a temperature of 373K and a pressure of one atmosphere (1 bar). The periodic boundary of the simulated system was centered on the protein, with the minimum distance between the protein edge and the box edge being set to 1.0 nm. The protein structure topology file was converted into a file recognizable by GROMACS using the pdb2gmx tool. The force field parameter was AMBEff14SB^[9]^. A model of transferable intermolecular potential with 3 points (TIP3P) was adopted for the water^[10]^. After that, the steepest descent method was applied to minimize the system energy. In the case of protein position restriction, the following simulations were performed: a 1000 ps constant number of particles-volume-temperature (NVT) equilibrium simulation and a 1000 ps constant number of particles-pressure-temperature (NPT) equilibrium simulation^[11]^. After NVP and NPT equilibration, the system performed a 200 ns final simulation with a timestep of 2 fs. The covalent bond length was restricted using a linear constraint solving algorithm, and the long-range electrostatic interactions were calculated using the particle mesh Ewald (PME) method^[6]^. After the simulation was completed, the gmx module was employed to calculate the radius of gyration (Rg), root mean square deviation (RMSD), solvent accessible surface area (SASA), and H-bond number. The final structure was taken as representative of the amorphous domain of silk fibroin.

***Construction of the ChCl/AA PDES box:*** The molecular structures of ChCl (CID: 6209) and AA (CID: 6581) in PDES were obtained from the PubChem database (https://pubchem.ncbi.nlm.nih.gov/). The AmberTools tool^[12]^ was applied to convert the ChCl and AA molecules into topology files in the itp format recognized by GROMACS, which were then treated with the GAFF force field^[13]^. The molecular structures of ChCl and AA were optimized by Gaussian software^[14]^, and the corresponding RESP2 charges were generated using Multiwfn^[15, 16]^. Subsequently, molecular dynamics simulations were performed using the GROMACS 2021.5 software package to construct the ChCl/AA PDES box. For the simulation, 100 ChCl (with Cl^-^ in a free state) and 200 AA molecules were placed in a box with dimensions of 5 nm × 5 nm × 5 nm. After completing the initial system construction, the steepest descent method was adopted for all atoms to minimize the system energy. Finally, 1000 ps of NVT equilibrium simulations and 10000 ps of NPT equilibrium simulations were performed to obtain the final conformation as the PDES solvent box for subsequent simulations.

***Molecular dynamics simulation of crystalline and amorphous domains:*** All simulations for crystalline and amorphous domains were carried out by the GROMACS 2021.5 software package^[8]^. The simulations were conducted in a closed environment, with temperature controlled at 373 K by the V-rescale method and pressure controlled at 1 bar by the Parrinello-Rahman method. The periodic boundary conditions of the simulation system were centered on the protein, with the minimum distance between the protein edge and the box edge being set to 1.0 nm. First, the topological parameters of silk protein crystals and amorphous domains were created by implementing a built-in pdb2gmx module in the GROMACS program. The AMBER force field (AMBEff14SB) was used to describe the interactions between silk fibroin residues. For the simulation of the crystalline domain in ChCl/AA PDES, the crystalline structure model was placed in a simulation box with a dimension of 5.0 nm × 5.0 nm × 4.2 nm, which was filled with 209 ChCl molecules and 401 AA molecules. As for the simulation of the amorphous domain in ChCl/AA PDES, the amorphous structure model was placed in a simulation box with a dimension of 5.0 nm × 10.3 nm × 9.1 nm, filled with 1068 ChCl molecules and 2251 AA molecules. The simulation systems were further subjected to energy minimization by the steepest descent method. Subsequently, the simulated systems were conducted to the equilibration of 1000 ps of NVT followed by 1000 ps of NPT. After that, the equilibrated systems were performed for the final 100 ns of dynamics simulations with a time step of 2 fs. The covalent bond length was restricted by a linear constraint solving algorithm, and the long-range electrostatic interactions were calculated using the PME method. The cutoff distance for calculating Coulomb interactions, electrostatic interactions, and van der Waals interactions was set to 1.4 nm, and hydrogen bonds were constrained by using the LINCS algorithm^[17]^. Visual molecular dynamics (VMD) was employed to capture snapshots illustrating conformational changes. The gmx module in the GROMACS software package was utilized to calculate the radius of gyration (Rg), root mean square deviation (RMSD), secondary structure, and H-bond number.

**Supplementary Figures**


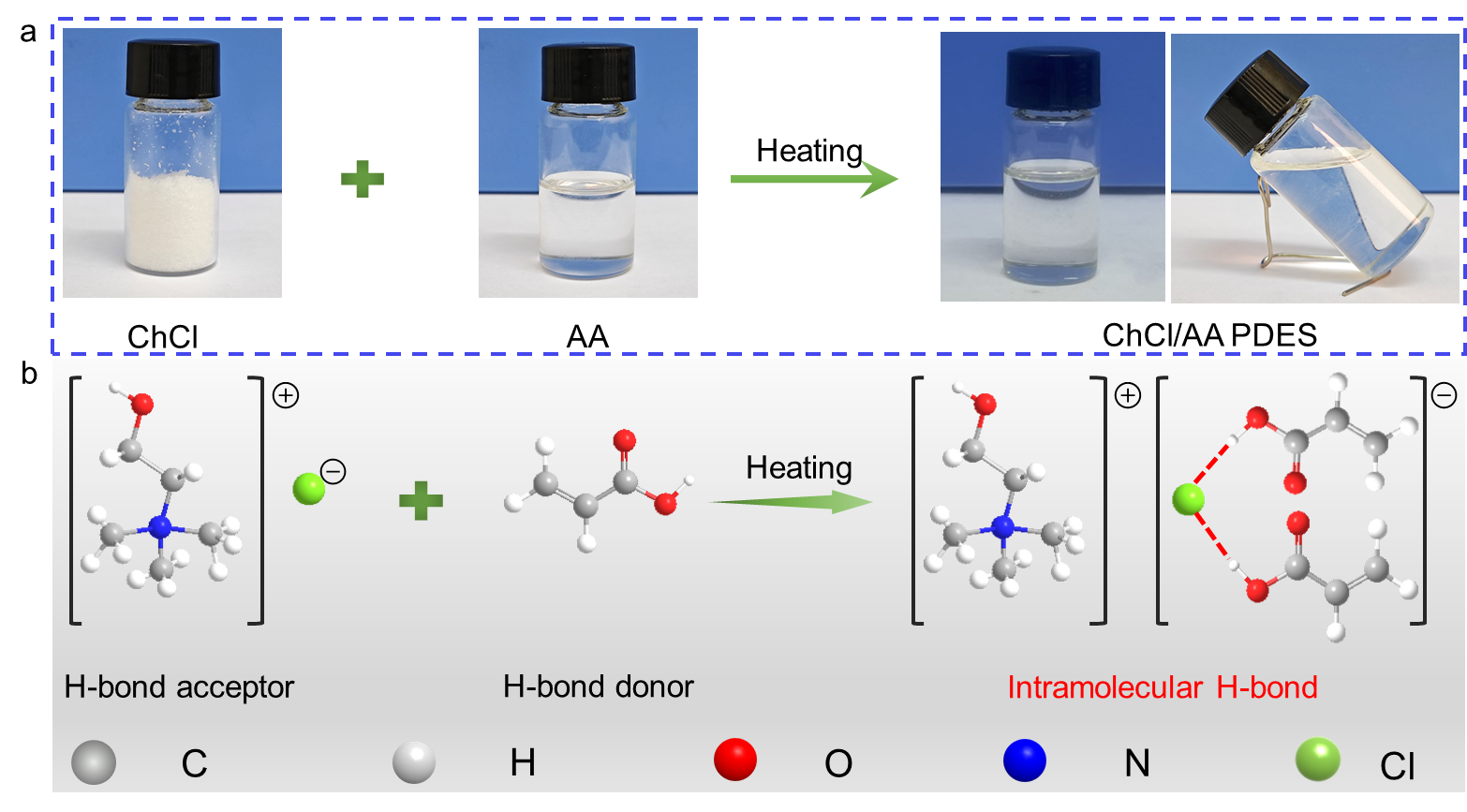


**Figure S1.** Preparation of PDES. (a) The formulation process of the PDES used in this work, consisting of ChCl and AA. (b) The formation of hydrogen bonding between ChCl and AA.


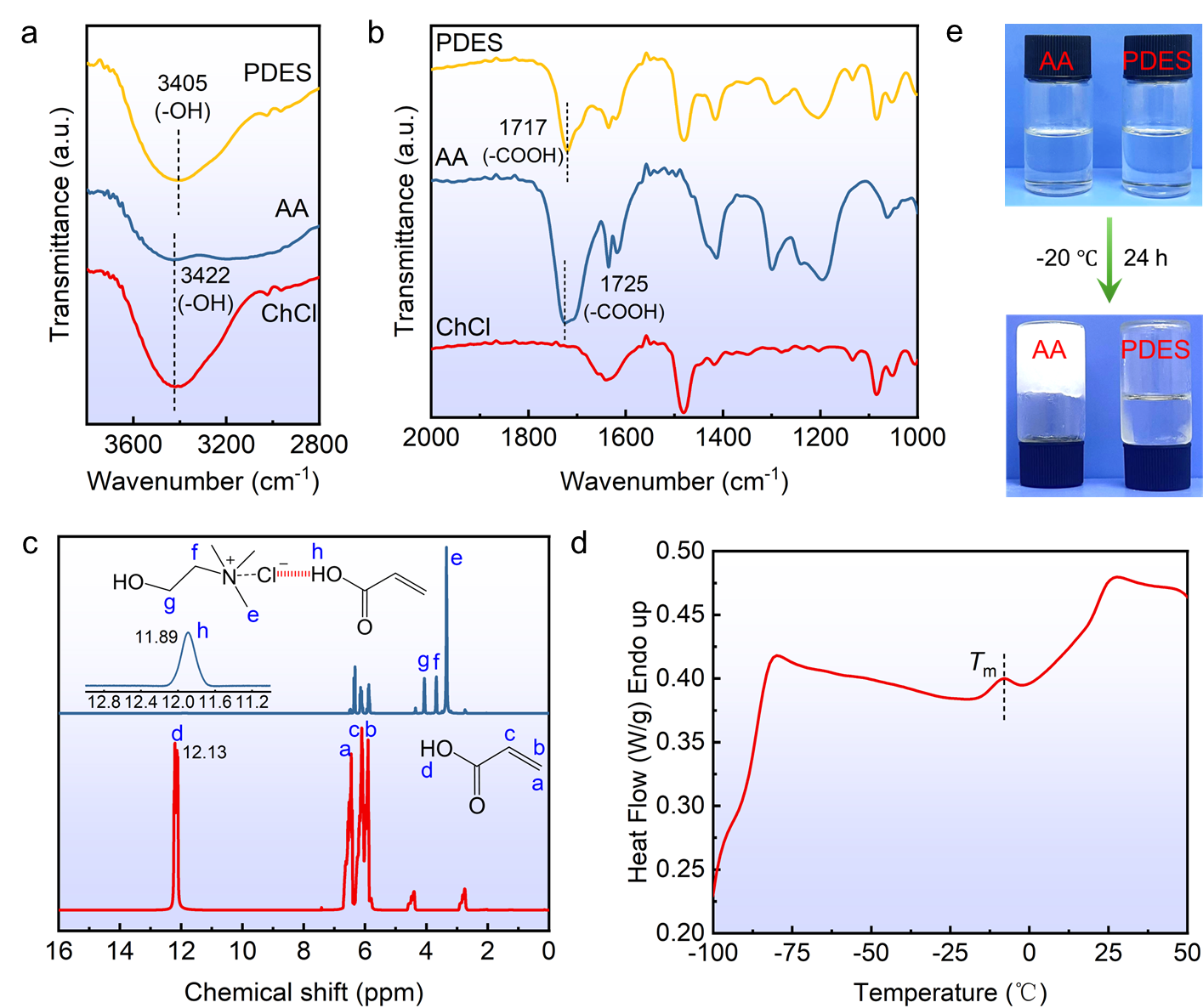


**Figure S2.** Chemical structure and property characterization of PDES. (a) FTIR spectra of PDES, ChCl, and AA. (b) ^1^H NMR spectra of PDES and AA. (c) DSC curve of PDES. (e) Optical photos of AA and PDES before and after being placed at -20 °C for 24 h.

The chemical structure of PDES was characterized by FTIR and 1H NMR. The FTIR spectrum of PDES showed a characteristic peak at 1717 cm^-1^ (Figure S2a), which was attributed to -COOH originated from AA, indicating that the carboxyl group of AA in the PDES system was in the form of acid rather than molten salt^[18, 19]^. Compared with ChCl and AA, the characteristic peaks of -OH and -COOH in PDES shifted to the lower wavenumber (Figure S2a, b), demonstrating that strong hydrogen bonds were formed between AA and ChCl. As shown in Figure S2c, no new proton peaks appeared in the ^1^H NMR spectrum of PDES, suggesting that ChCl and AA did not react during the heating process for preparing PDES. Meanwhile, the chemical shift of the carboxyl group proton peak in PDES shifted slightly to the lower field, further confirming the formation of strong hydrogen bonds between ChCl and the carboxyl group in AA^[20]^. In addition, the melting point (*T_m_*) of PDES was determined to be approximately -8 °C by DSC testing (Figure S2d), which is far lower than that of both ChCl (*T_m_* ≈ 302 °C) and AA (*T_m_* ≈ 14 °C) ^[18]^. This is attributed to the strong hydrogen bonds between ChCl and AA^[20]^. As evidenced in Figure S2e, PDES remained as a liquid even at temperatures as low as -20 °C, exhibiting typical characteristics of a deep eutectic solvent.


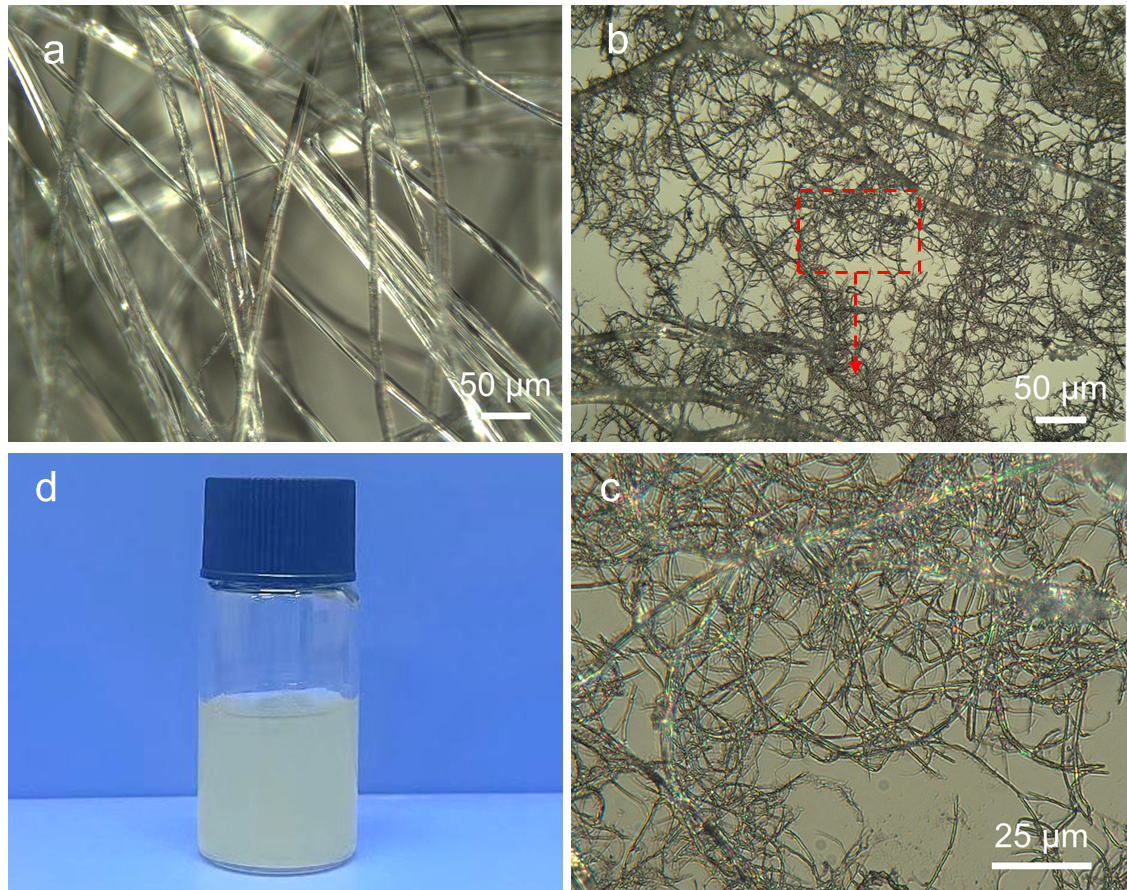


**Figure S3.** Optical microscopy images of (a) SF fibers and (b-c) SMNF. (d) Optical image of SMNF-PDES mixture.


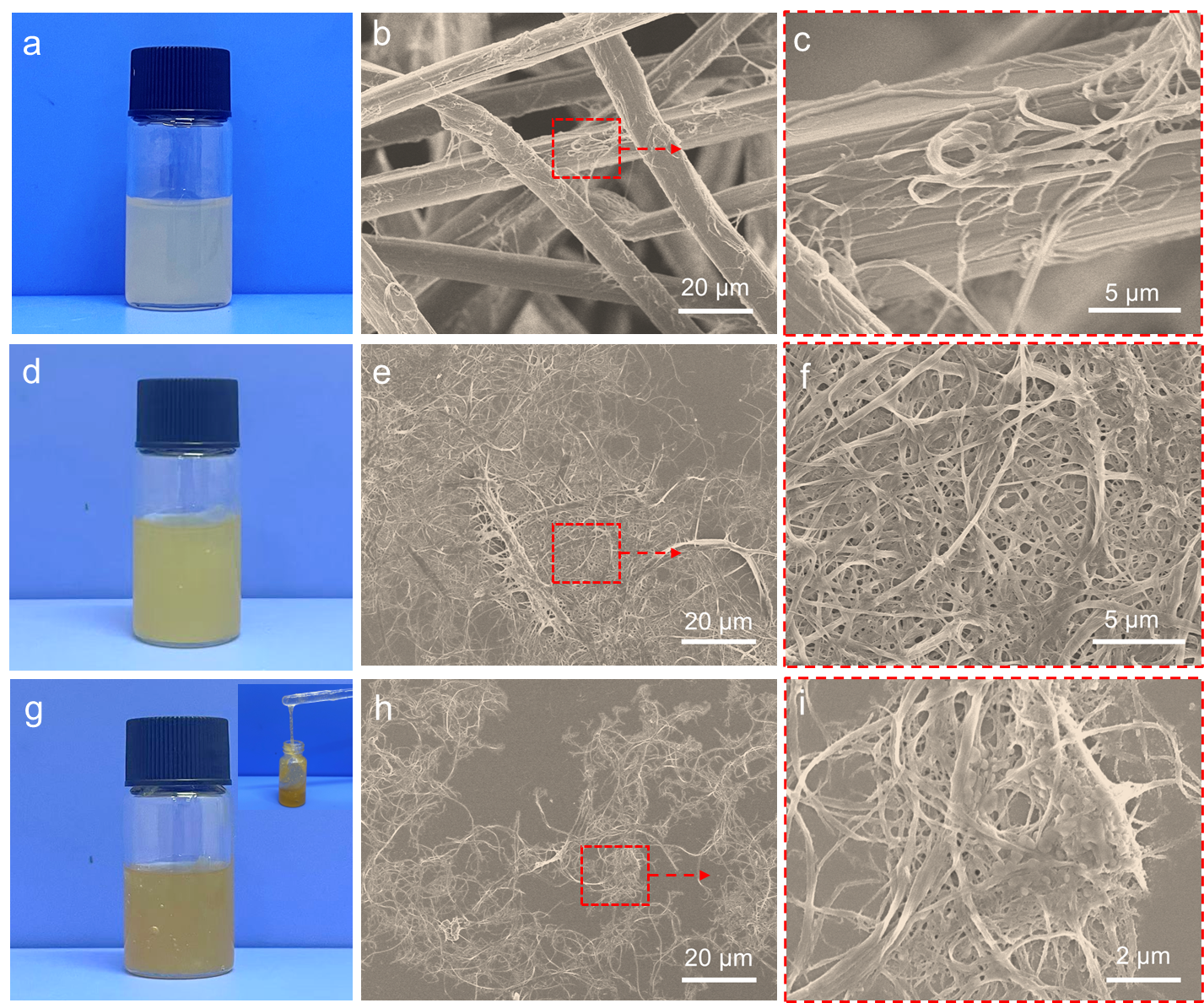


**Figure S4.** Deconstruction effect of PDES on SF fibers under different process conditions. (a-c) 90 ℃, 12 h; (d-e) 110 ℃, 4 h; (g-i) 130 ℃, 2 h.

The temperature and time of thermal stimulation are key factors affecting the in situ deconstruction of SF fibers by PDES to form SMNF. According to the formation time of the paste-like SMNF-PDES mixture and SEM morphology^[21]^, the effects of heating temperature and time on the deconstruction of SF fibers by PDES were analyzed. As shown in Figure S4a-c, after being treated with PDES at 90 °C for 12 h, there were only a few micro-nanofibrils on the surface of SF fibers, indicating that silk fibers were not effectively deconstructed by PDES under these process parameters. When the temperature was increased to 110 °C and 130 °C, the time required to form the paste-like SMNF-PDES mixture was 4 h and 2 h, respectively. Although the deconstruction efficiency of SF fibers by PDES improved at 110 °C and 130 °C, the SMNF-PDES mixture turned yellow due to the partial dissolution of SF fibers by PDES at high temperature (Figure S4d, g)^[22, 23]^. Especially, the SF dissolved in PDES at 130 °C significantly increased the viscosity of the system and even caused a wire-drawing phenomenon (the inset of Figure S4g), which was not favorable for the subsequent EGaIn droplet-induced polymerization reaction. SEM images revealed that the SMNF obtained from SF fibers treated by PDES at 110°C for 4 h mainly consisted of nanofibrils accompanied by some short rod-shaped microfibrils (Figure S4e, f). Additionally, some SF fibers were dissolved into nanoparticles by PDES after being treated at 130 °C for 2 h (Figure S4h, i). These results indicated that PDES destroyed the original hierarchical structure of SF fibers at 110 °C and 130 °C, which was not conducive to the subsequent construction of biomimetic eutectic gel multiscale structures. Therefore, considering energy consumption and deconstruction efficiency, and avoiding damage to the mesostructures of SF fibers by high temperatures during the deconstruction process, the process conditions to deconstruct SF fibers by PDES were set at 100 °C for 5 h.


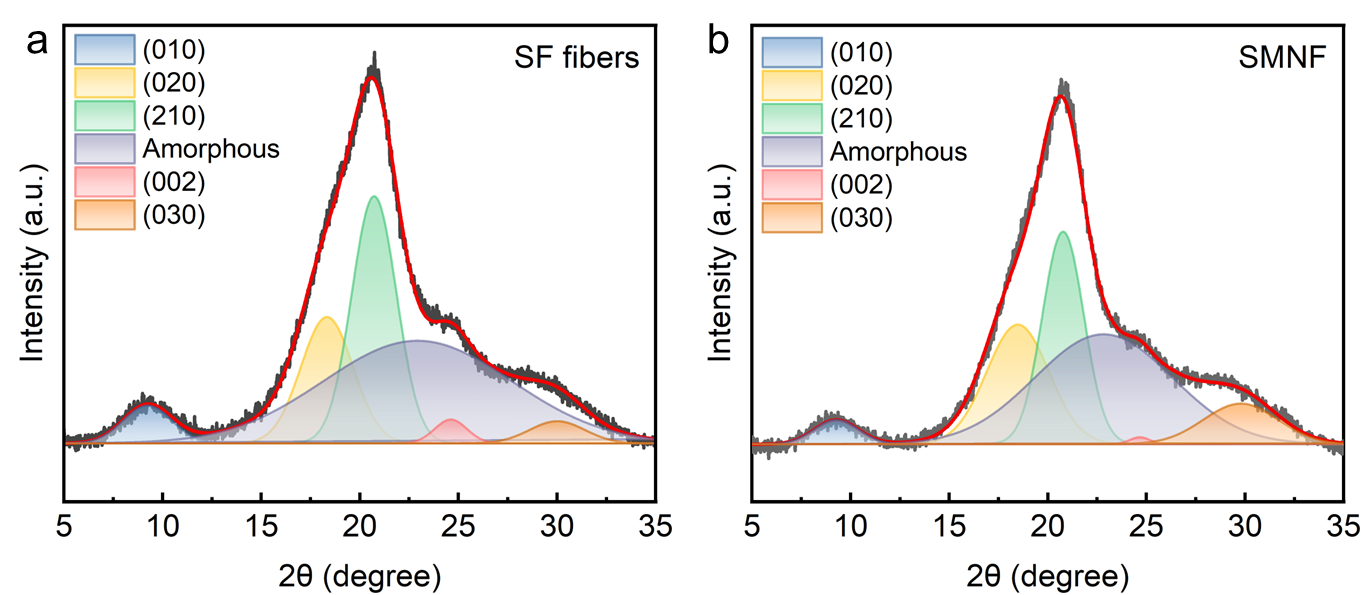


**Figure S5.** The deconvolution results of the XRD patterns of (a) SF fibers and (b) SMNF.


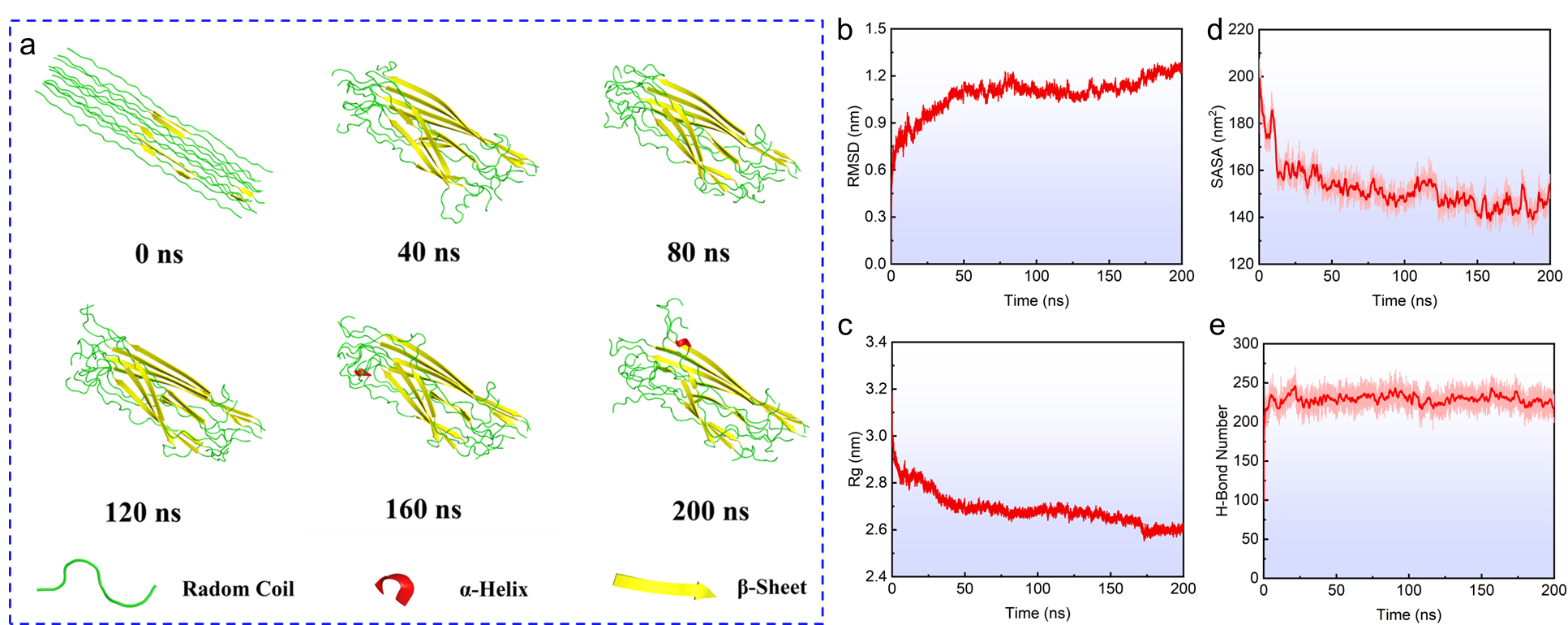


**Figure S6.** Construction and rationality analysis of the amorphous domain model. (a) Representative snapshots of the conformation evolution of the amorphous domain during the simulation. Changes in (b) root mean square deviation, (c) radius of gyration, (d) solvent accessible surface area, and (e) H-bond number of the amorphous domain during the simulation.

To construct the structure model of the amorphous region of silk fibroin, we used Avogadro software to assemble 12 fully-extended peptides (TGSSGFGPYVANGGYSGYEYAWSSESDFGT) into a 4×3 antiparallel array, with an initial chain distance of 5 Å, to simulate the initial state of random coil and partially ordered structures. Subsequently, 200 ns of dynamic simulation in an explicit solvent environment was carried out on the structure to obtain the pre-equilibrated amorphous domain. The final structure was taken as representative of the amorphous domain of silk fibroin.

Figure S6a presents representative snapshots of conformation evolution during the simulation process. In the simulation process, the polypeptide chains gradually evolved from a fully-extended state to a conformation with partial aggregation and folding, and formed partial β-sheet structures, highlighting the tendency of the simulation system to undergo structural rearrangement in a water environment. The formation of such partial secondary structures is consistent with the local order features of the amorphous domain of natural SF^[6, 7, 24]^. From the perspective of conformational stability, the root mean square deviation (RMSD) of the amorphous domain gradually increased over time and eventually stabilized at approximately 1.2 nm (Figure S6b), indicating that the system reached thermodynamic equilibrium after initial conformational relaxation. The overall stability of the model was satisfactory. As a corresponding result, the gyration radius (Rg) of the amorphous domain gradually decreased and stabilized at around 2.6 nm (Figure S6c), revealing that the polypeptide chains gradually transformed from an initial loose arrangement to a more compact conformation distribution. This feature corresponds to the loose but not fully unfolded structure characteristic of natural amorphous domains^[25]^. Figure S6d shows that the solvent accessible surface area (SASA) decreases rapidly in the initial stage and stabilizes after 150 ns. This result suggests that the aggregation nuclei are gradually formed among polypeptide chains, reducing the contact area with water molecules, thereby reflecting a convergence tendency from a loose to a compact conformation. Furthermore, the H-bond number in the system was relatively stable and sustained at a high level (200-250) with small fluctuations (Figure S6e), implying that the formation of the stable hydrogen bond networks during aggregation was beneficial for maintaining the overall stability of the conformation.

Based on the comprehensive results of conformation stability, compactness, solvent exposure changes, and hydrogen bond networks, the constructed amorphous domain model is relatively reasonable. It can effectively simulate the conformation characteristics of the amorphous domain of natural silk protein, which is suitable for subsequent simulation analysis in PDES.


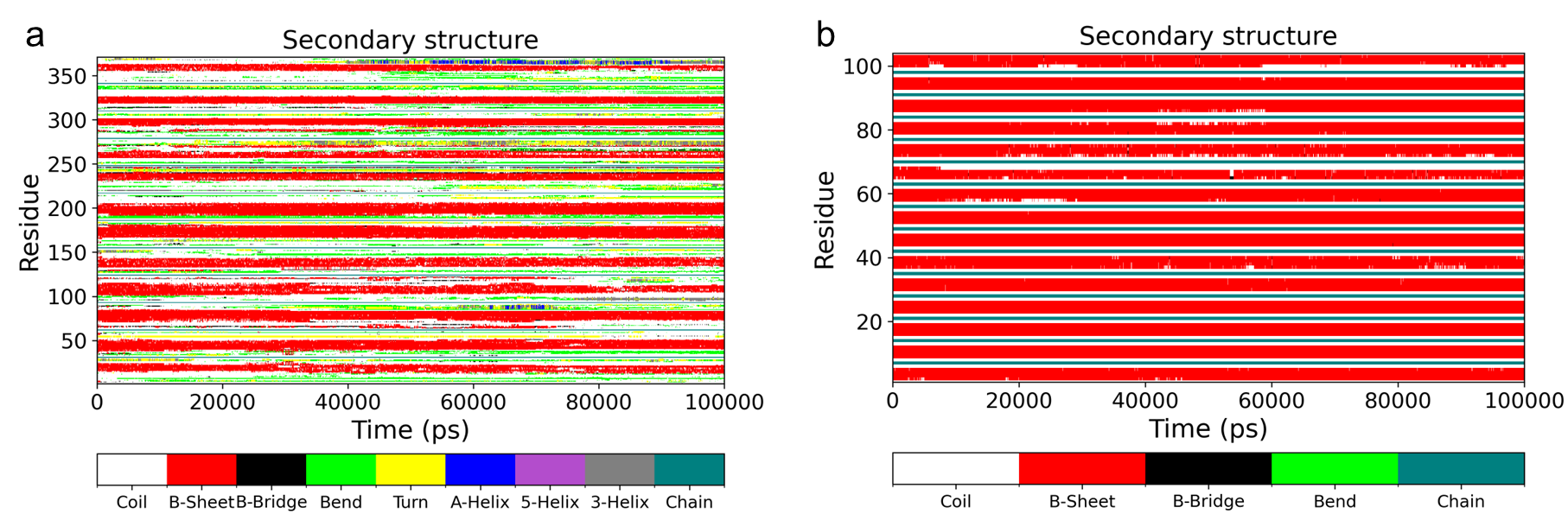


**Figure S7.** Time evolution of secondary structures (Timeline analysis) in (a) amorphous domain and (b) crystalline domain. The representative amorphous domain contains 12 polypeptide chains, while the representative crystalline domain has 15 polypeptide chains.


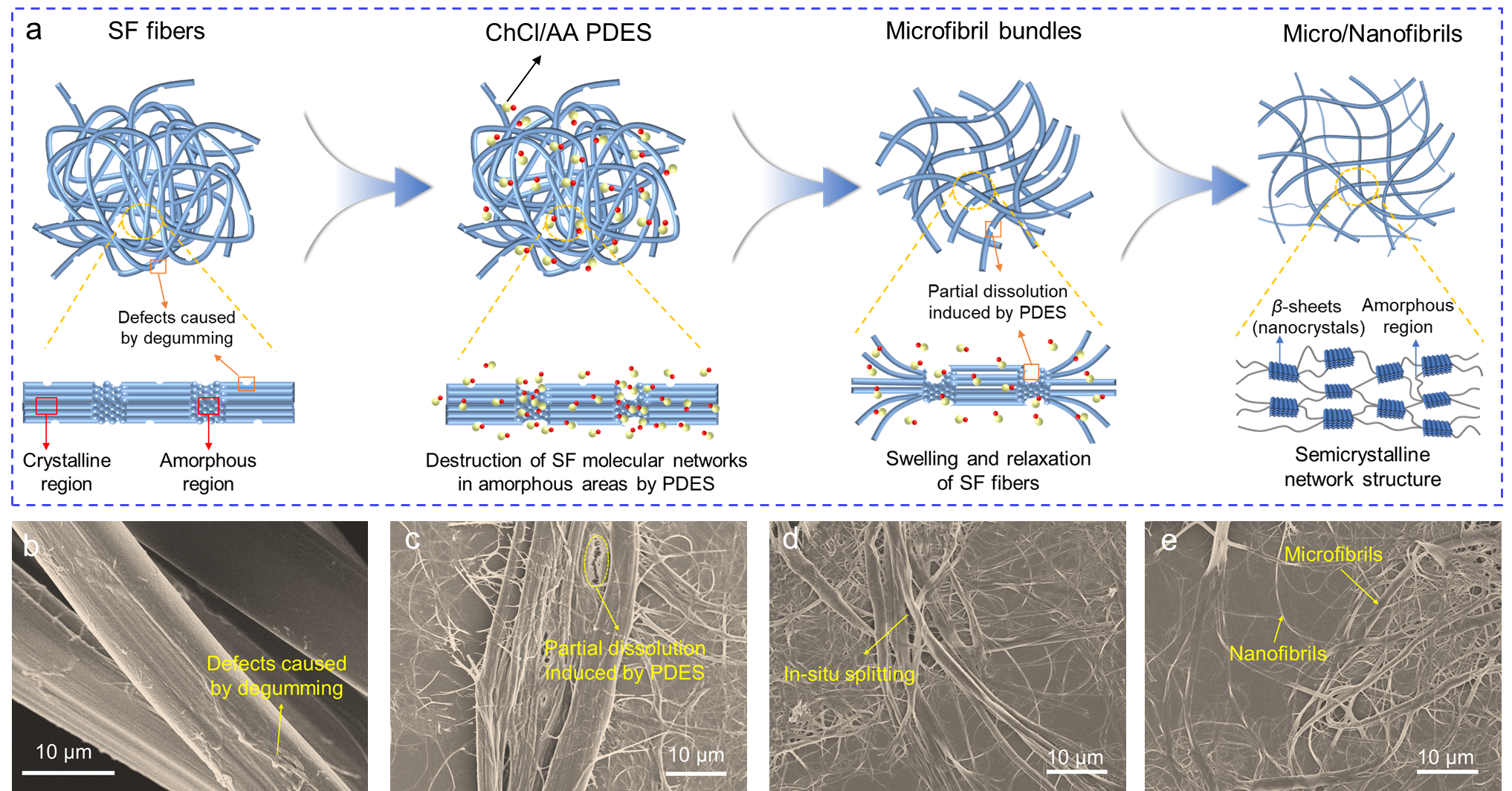


**Figure S8.** (a) Schematic illustration of the process and mechanism of SF fibers deconstructed by PDES. (b-e) Micro-morphology evolution of SF fibers deconstructed by PDES.


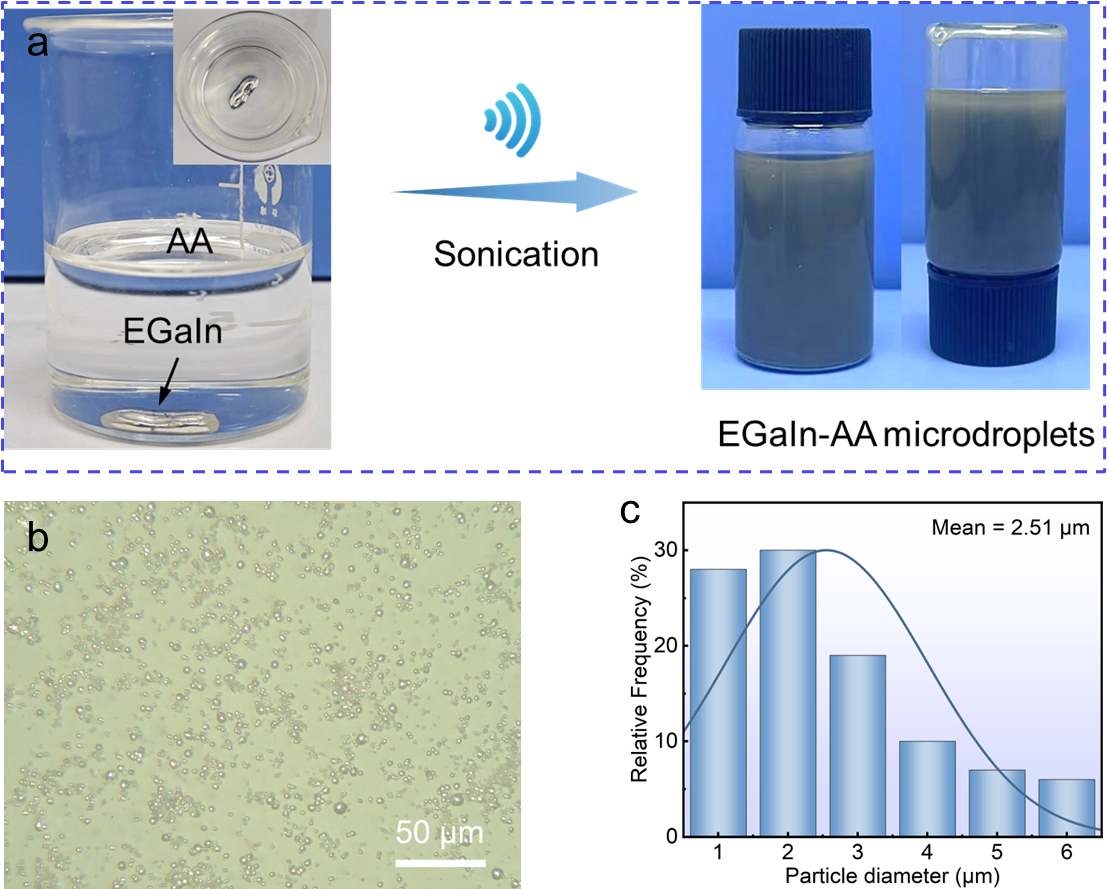


**Figure S9.** (a) Preparation of EGaIn-AA microdroplet suspension by AA interface modification combined with ultrasonication. (b) Optical microscope image and (c) particle size distribution of EGaIn-AA microdroplets.


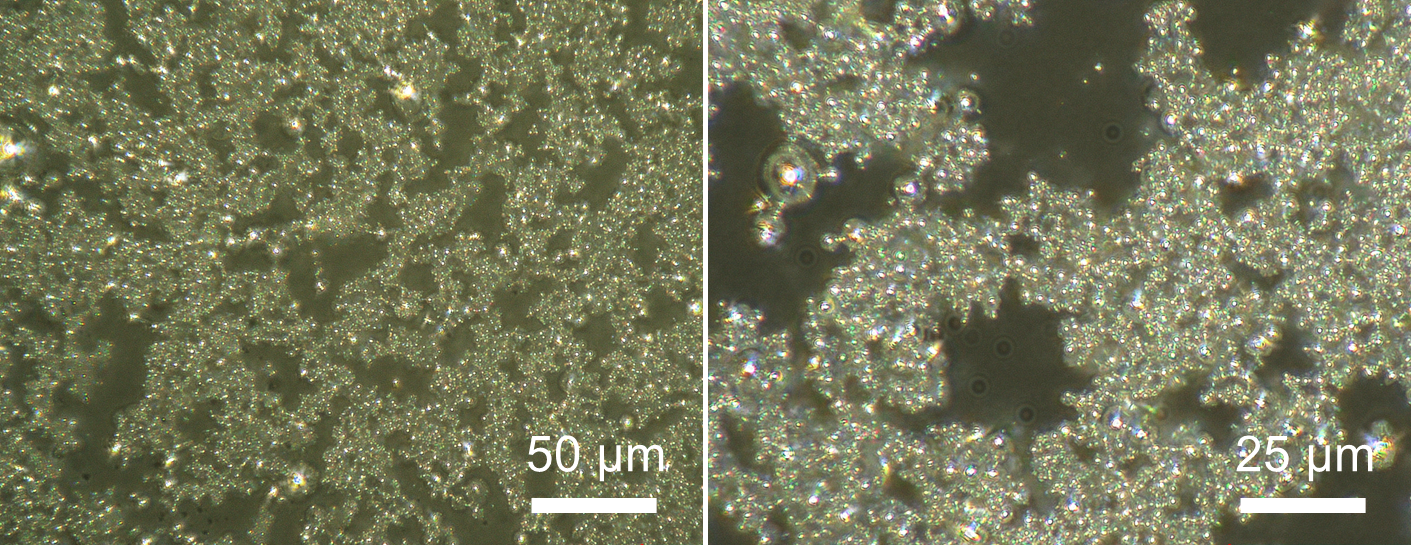


**Figure S10.** Optical microscope images at different magnifications showing the aggregation of EGaIn-AA droplets prepared by sonicating 1.0 g of bulk EGaIn in 14 g AA.


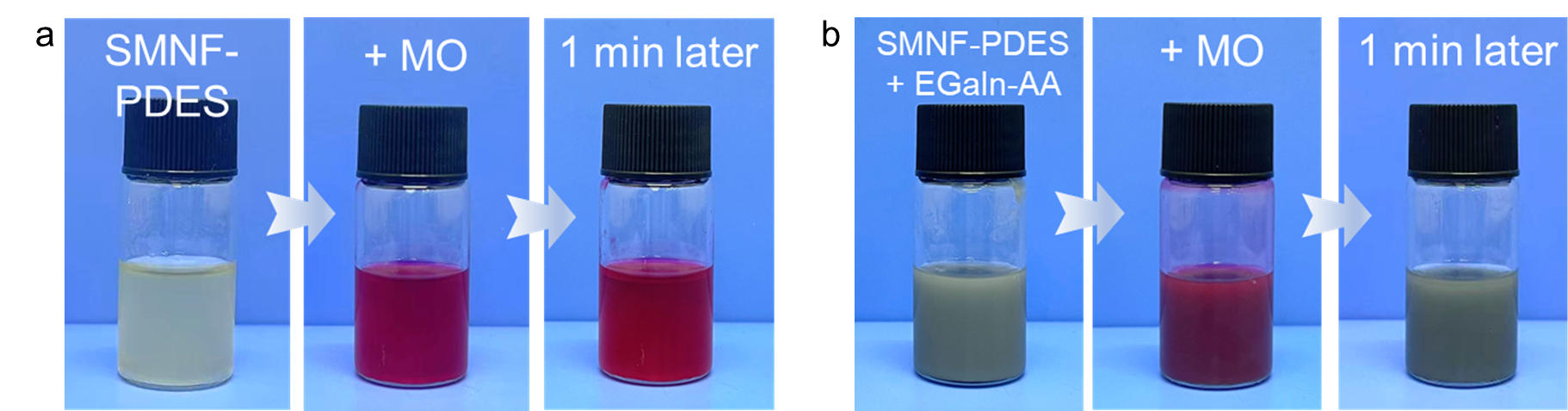


**Figure S11.** Digital photos showing color changes of (a) SMNF-PDES mixture and (b) SMNF-PDES + EGaIn-AA mixture after adding methyl orange (MO) aqueous solution.


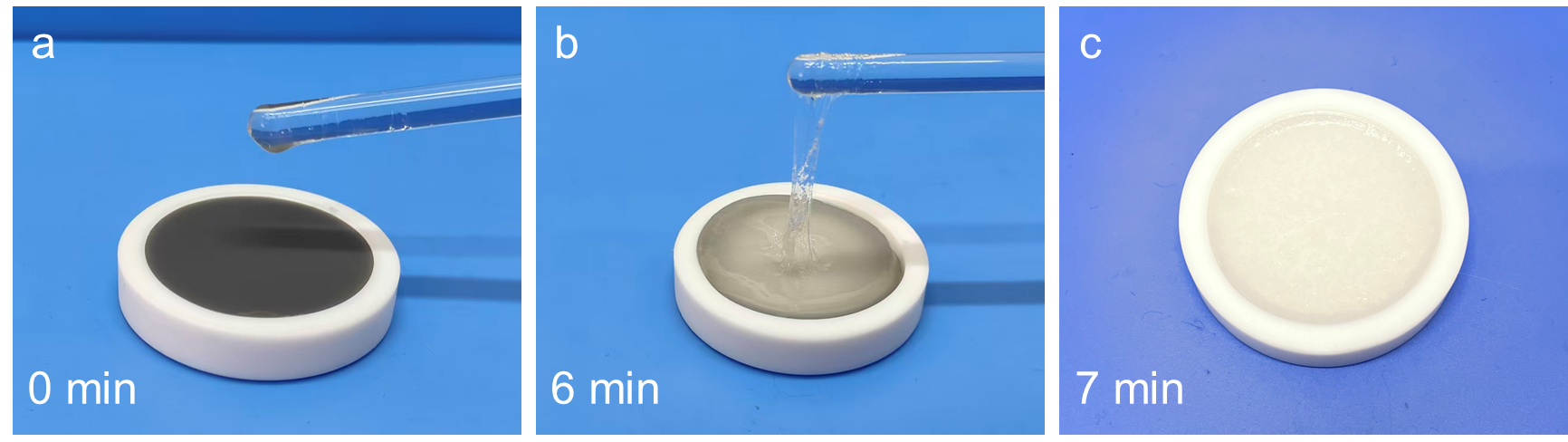


**Figure S12.** Digital images of polymerization process. (a) SMNF-PDES + EGaIn-AA mixture with a low viscosity at 0 min. (b) High-viscosity SMNF-PDES + EGaIn-AA mixture exhibiting the drawing phenomena at 6 min. (c) Solid SMNF-Egel formed at 7 min.


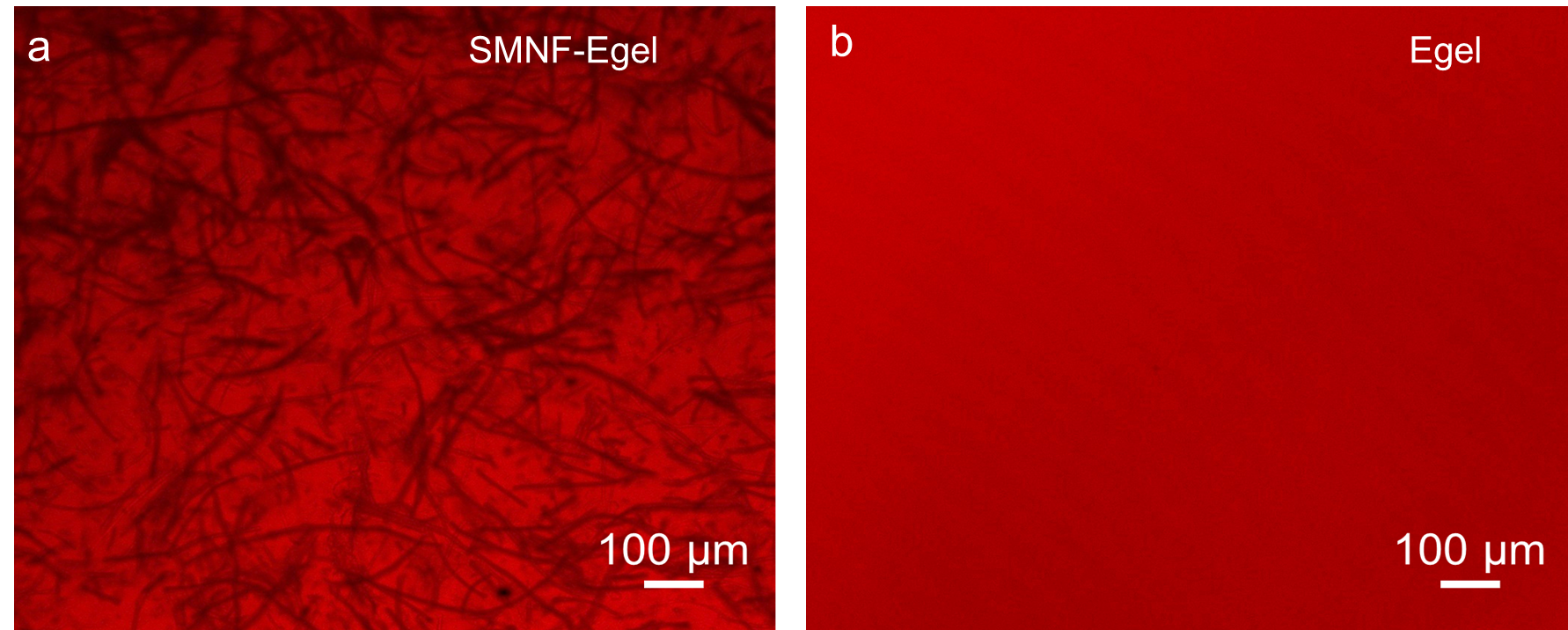


**Figure S13.** The 2D laser scanning confocal microscope images of (a) SMNF-Egel and (b) Egel.


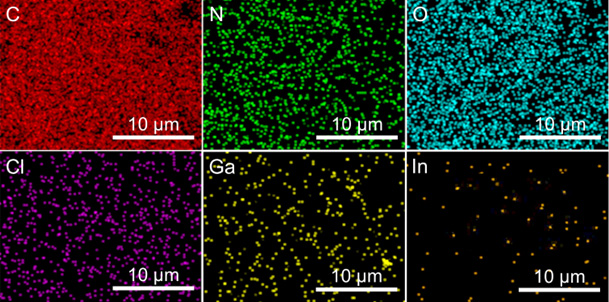


**Figure S14.** EDS elemental mapping images of C, N, O, Cl, Ga, and In of the SMNF-Egel.


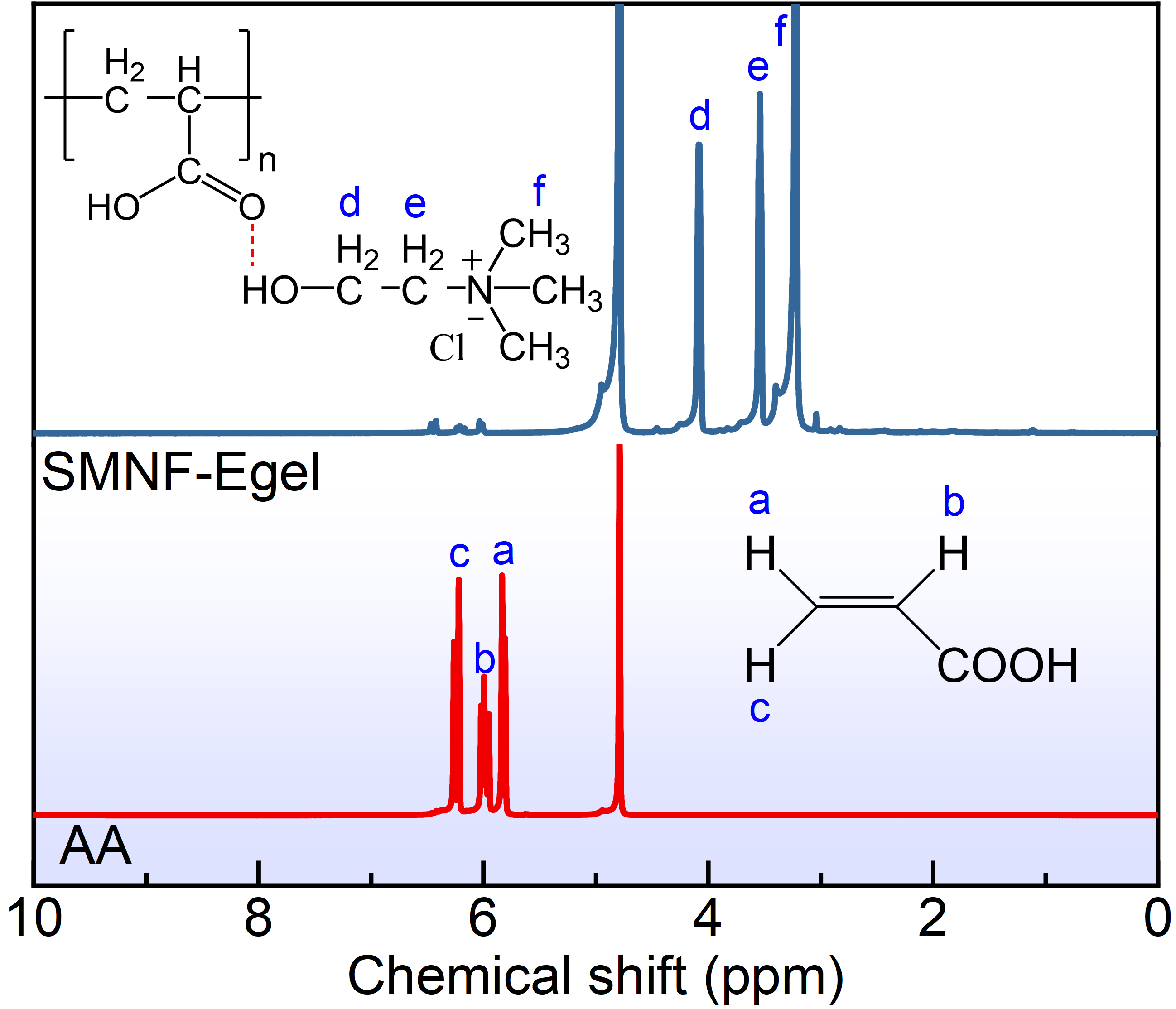


**Figure S15.** ^1^H NMR spectra of SMNF-Egel and AA.


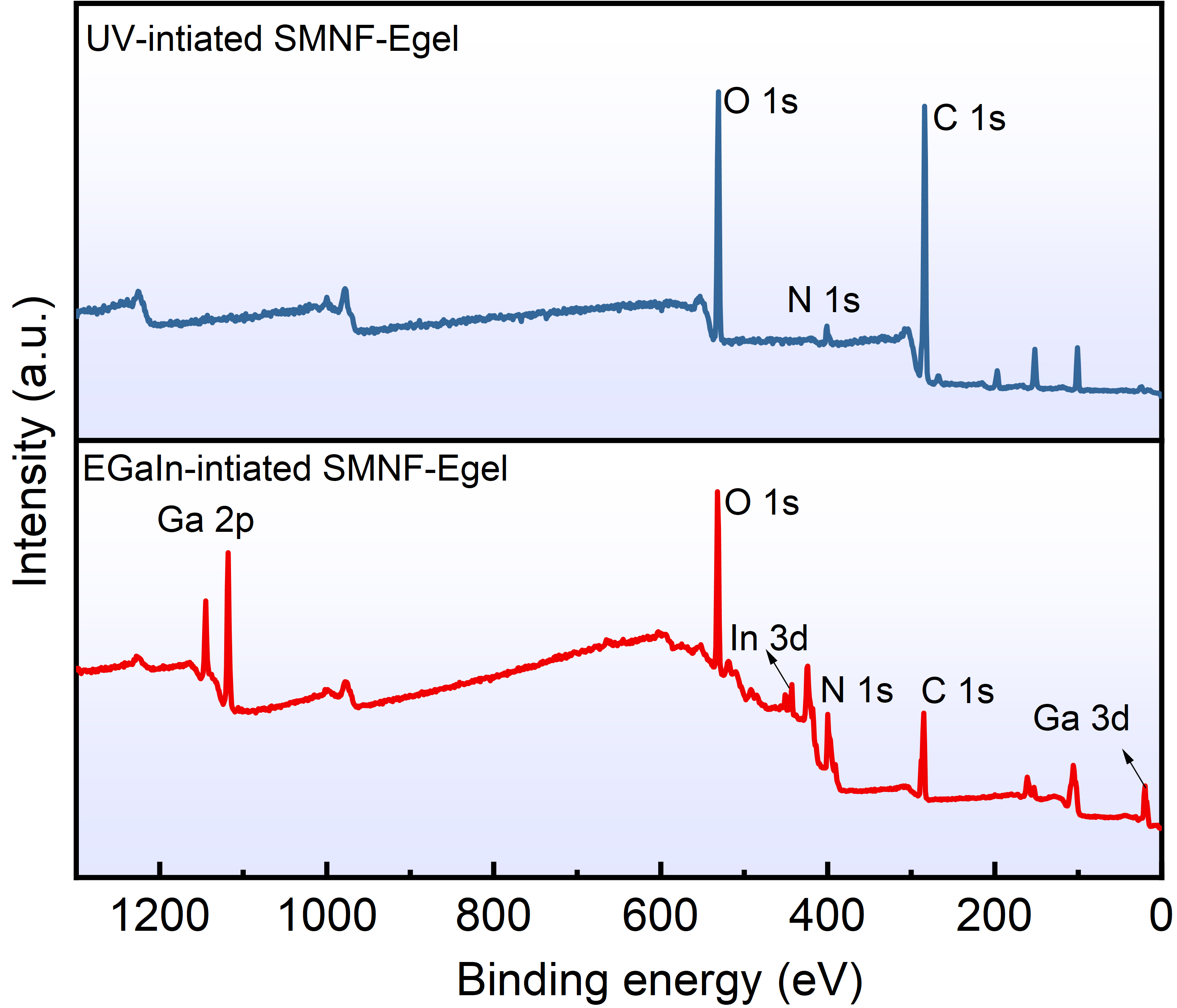


**Figure S16.** The wide-range XPS spectra of UV-initiated SENF-Egel and EGaIn- initiated SENF-Egel.


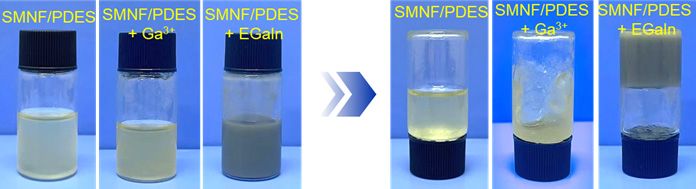


**Figure S17.** Digital photos of SMNF-PDES, SMNF-PDES + Ga^3+^, and SMNF-PDES + EGaIn-AA mixtures.


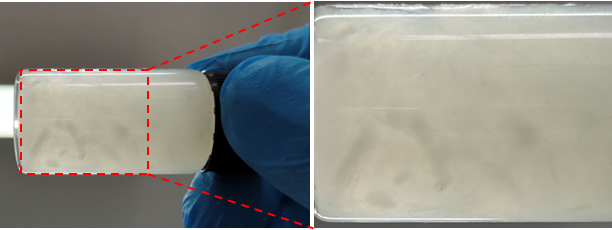


**Figure S18.** The digital photo of the SMNF-PDES-1.4 mixture (with SF fiber content of 1.4% by PDES mass) showing that SF fibers are difficult to be deconstructed by PDES to form a uniformly dispersed mixture.


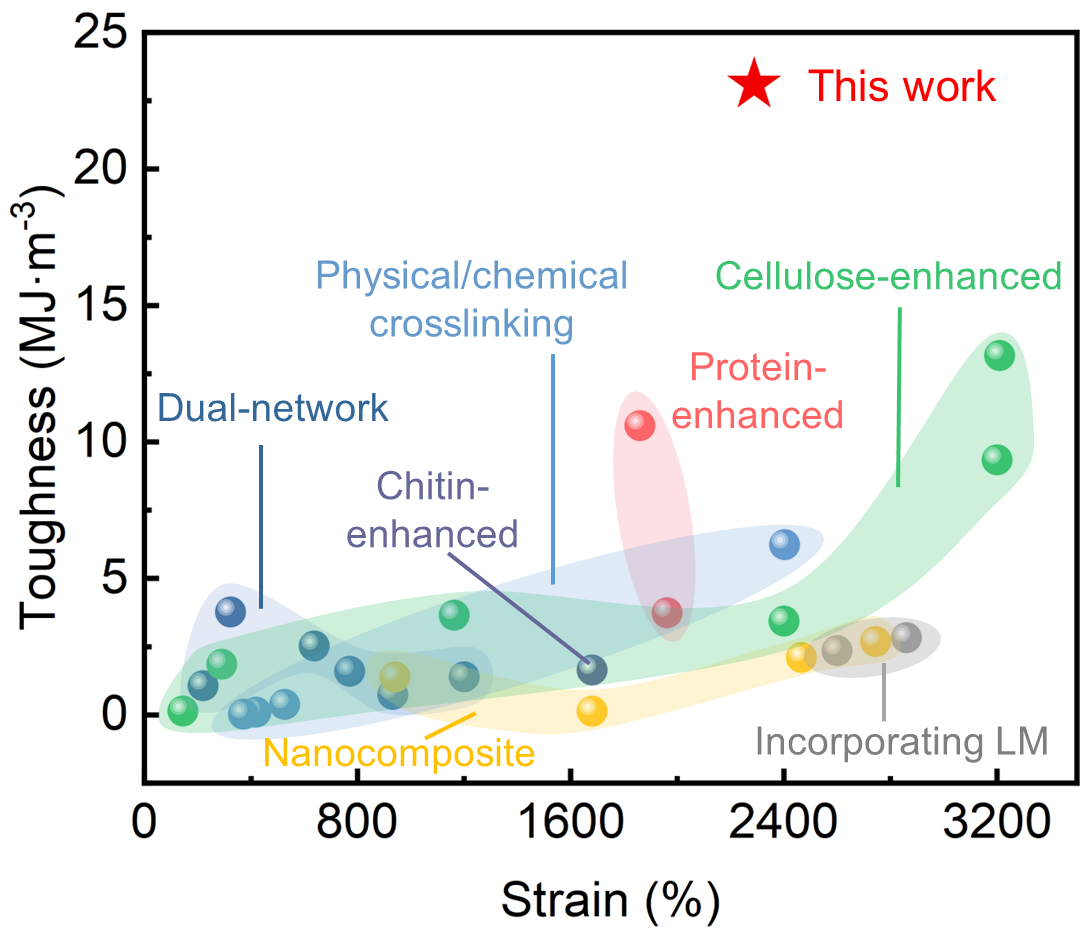


**Figure S19.** Ashby plot of toughness and strain of SMNF-Egel-1 and the reported PDES-based eutectogels.


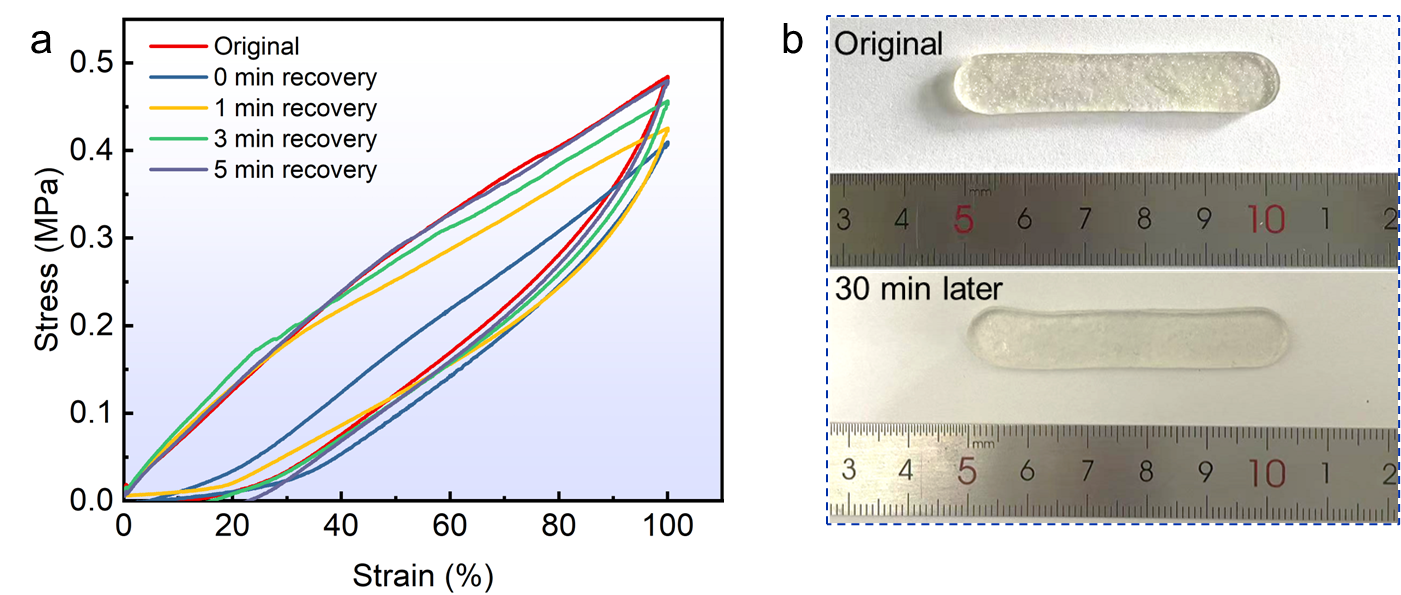


**Figure S20.** (a) Loading-unloading curves under 100% strain at different residence times. (b) Optical photos of the cyclically stretched (100% strain, 100 cycles) SMNF-Egel-1 after resting for 30 min.


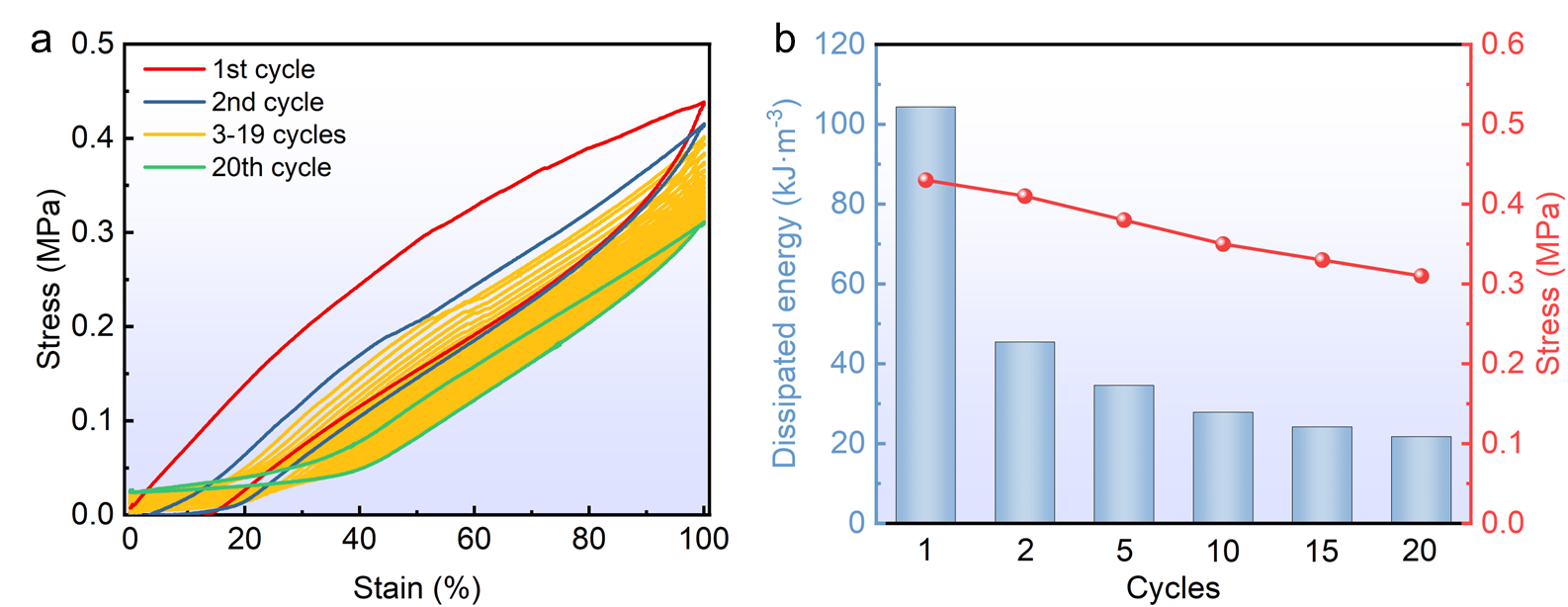


**Figure S21.** (a) Cyclic loading-unloading tensile tests of SMNF-Egel-1 at 100% strain for 20 times. (b) The corresponding maximum tensile stress and dissipated energy per cycle.


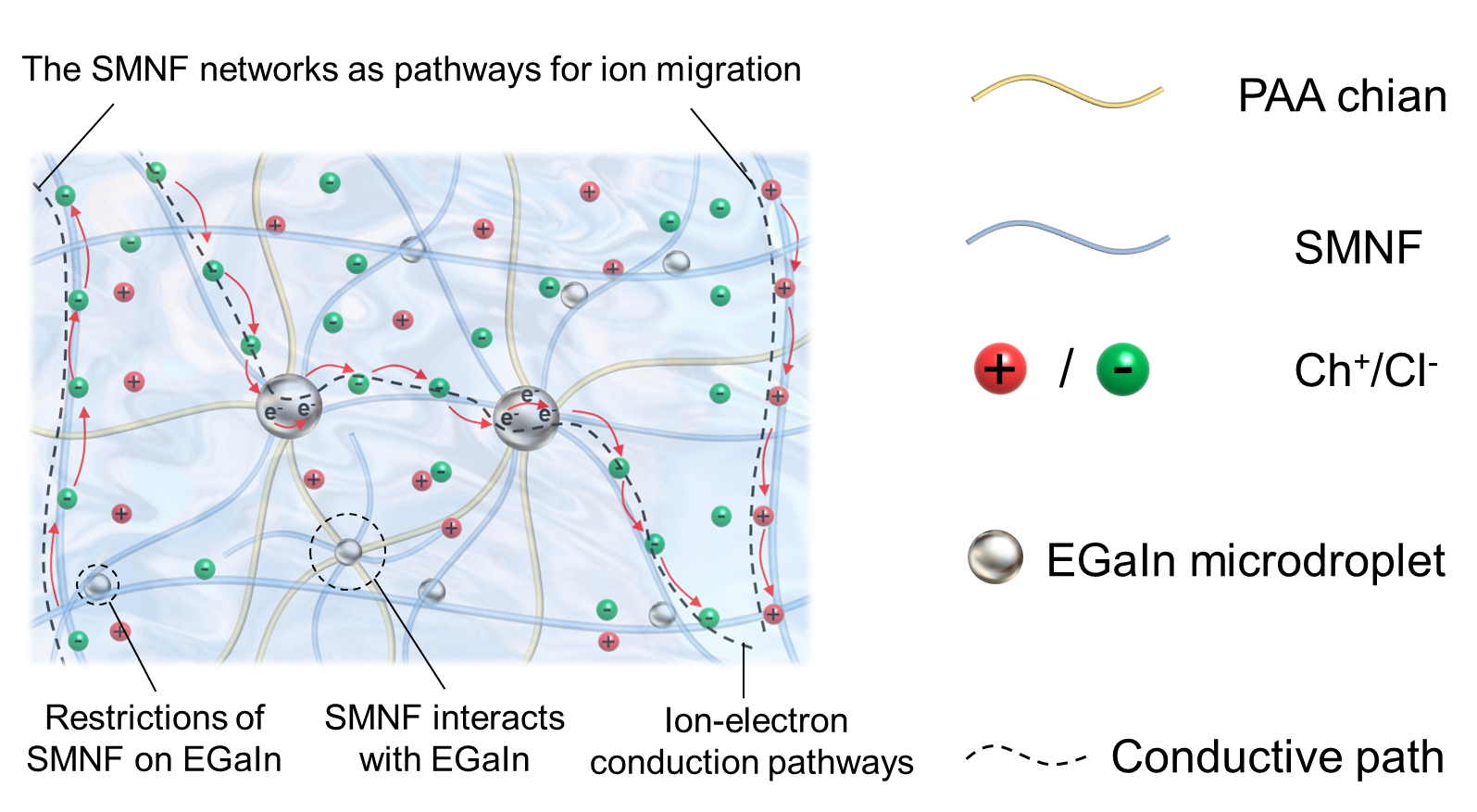


**Figure S22**. Schematic diagram of the conductive mechanism of SMNF-Egel.


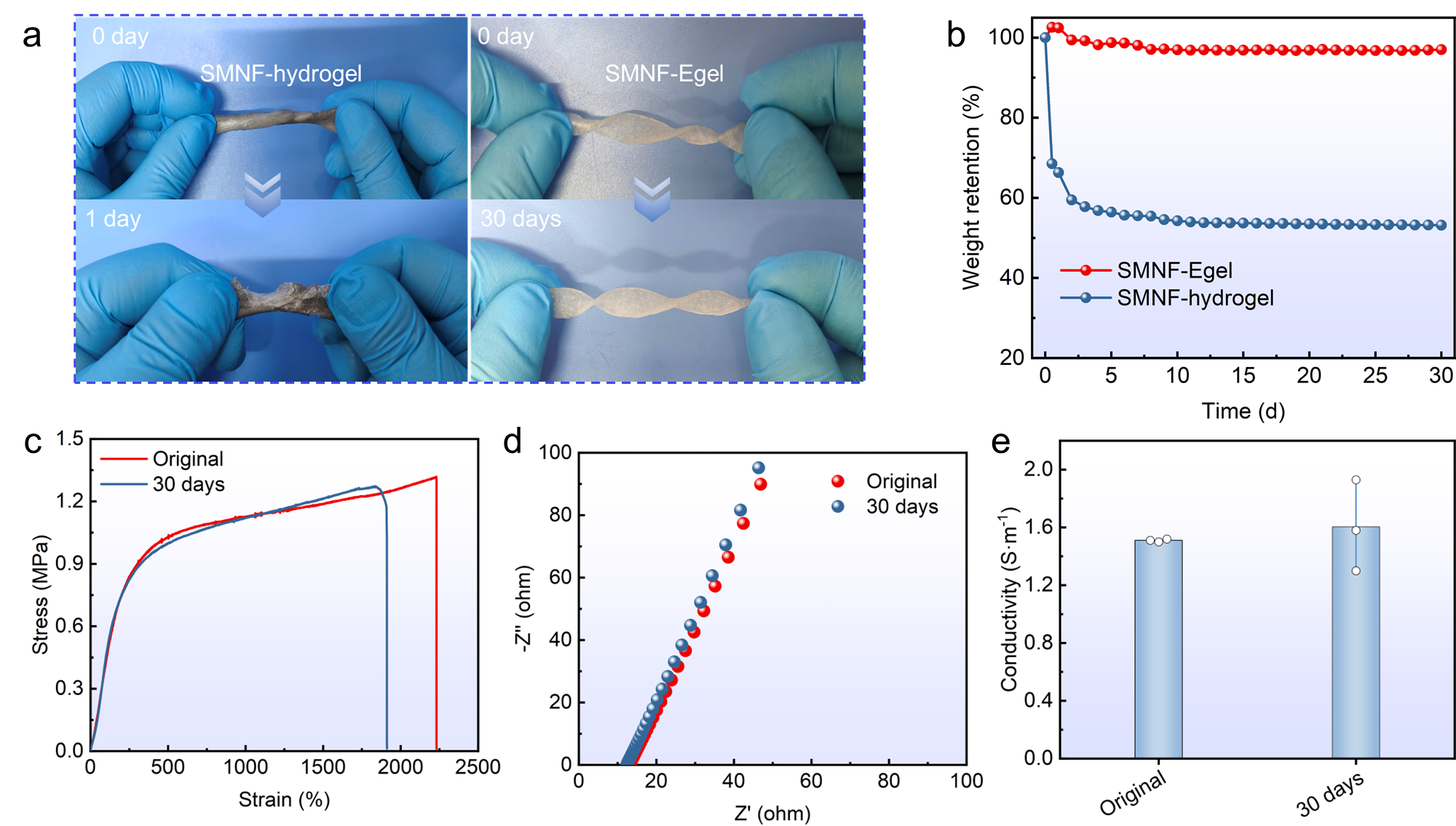


**Figure S23.** Anti-drying properties of SMNF-Egel. (a) Digital photos of SMNF-Egel left for 30 days and SMNF-hydrogel left for 1day at ambient conditions. (b) Mass changes of SMNF-Egel and SMNF-hydrogel during 30 days of storage at ambient conditions. (c) Mechanical properties of SMNF-Egel before and after being left at ambient conditions for 30 days. (d) The Nyquist curves and (e) calculated ionic conductivities of SMNF-Egel after being placed in ambient environment for 30 days. Data in (e) are presented as mean ± SD, n = 3.

Traditional conductive hydrogels containing large amounts of water suffer from poor environmental stability due to freezing or evaporation in extreme environments, which severely restricts their long-term reliability and durability^[26]^. Fortunately, the introduction of lower melting point and non-volatile PDES can effectively enhance low or high-temperature resistance, enabling eutectogels to function at harsh conditions^[27]^. As shown in Figure S23a,b, after being exposed to ambient conditions for 30 days, SMNF-Egel still exhibited excellent flexibility with a weight loss rate of only 3.04%, confirming its superior anti-drying properties. Conversely, SMNF-hydrogel was severely dehydrated after only 1 day, with a weight loss rate of 33.70%, which significantly impaired its tensile properties and caused it to rupture easily after stretching. Notably, the tensile strength of SMNF-Egel remained almost unchanged after being left at ambient conditions for 30 days, but its elongation at break decreased slightly, remaining above 1800% (Figure S23c). This may be due to slight densification and enhanced physical cross-linking of the polymer network segments during storage^[26]^. Furthermore, the conductivity of the SMNF-Egel did not decrease significantly after 30 days (Figure S23d,e). These results collectively demonstrate the outstanding long-term stability of the SMNF-Egel under ambient conditions.


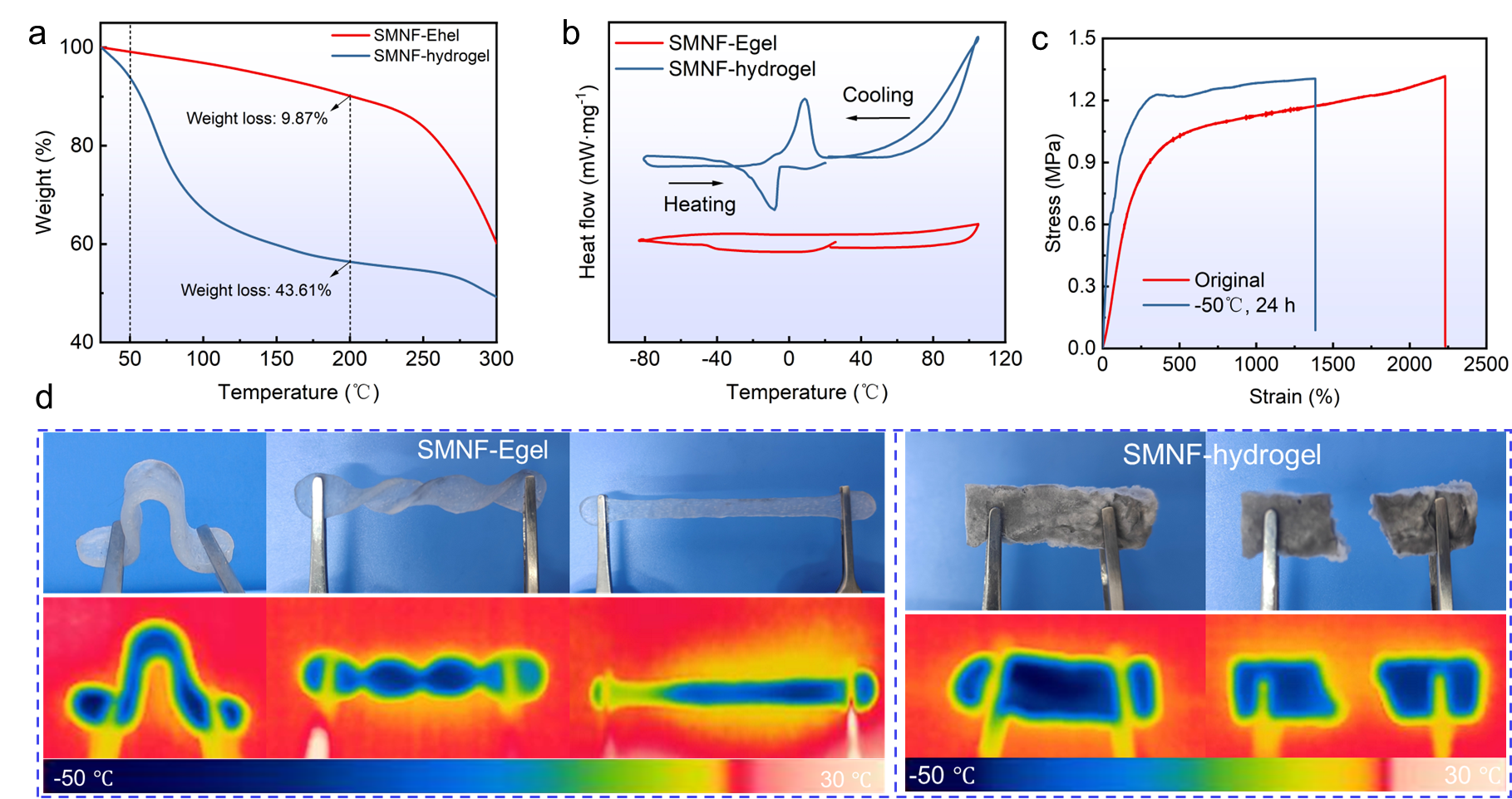


**Figure S24.** Thermal stability and anti-freezing properties of SMNF-Egel. (a) TG curves and (b) DSC curves of SMNF-Egel and SMNF hydrogel. (c) Mechanical properties of SMNF-Egel before and after being stored at -50 °C for 24 h. (d) Digital photos and infrared thermal images of SMNF-Egel and SMNF-hydrogel after being stored at -50 °C for 24 h.

We analyzed the thermal stability of SMNF-Egel and SMNF-hydrogel using TG (Figure S24a). At 200°C, the weight loss rate of SMNF-Egel was only 9.87%, while that of SMNF-hydrogel was as high as 43.61%, proving that SMNF-Egel demonstrates exceptional thermal stability and applicability over a wide temperature range. The anti-freezing properties of SMNF-Egel and SMNF-hydrogel were evaluated by using DSC. As presented in Figure S24b, SMNF-hydrogel exhibited a distinct exothermic peak at -7.9 °C, indicating that the hydrogel began to freeze at this temperature. In contrast, SMNF-Egel showed no exothermic peaks within the temperature range of -80 to 100 °C, demonstrating its excellent anti-freezing property. Figure S24d intuitively demonstrates the superior anti-freezing property of SMNF-Egel. Compared to SMNF-hydrogel, SMNF-Egel did not freeze after being placed at -50 °C for 24 h and could still withstand various mechanical deformations such as bending, twisting, and stretching. In addition, the mechanical strength of SMNF-Egel increased after being frozen at -50 °C for 24 h (Figure S24c,), while the elongation at break decreased due to the formation of trace amounts of ice crystals^[26]^, further verifying its excellent mechanical properties.

All the above data confirm that SMNF-Egel exhibits outstanding environmental stability, including long-term ambient stability (anti-drying properties), thermal stability, and anti-freezing properties. These properties originate from the extensive hydrogen bond network within the eutectogels framework^[28]^, which not only effectively retains solvents but also endures high temperatures while significantly depressing the freezing point, thereby enabling SMNF-Egel to maintain structural integrity and functionality under extreme conditions.


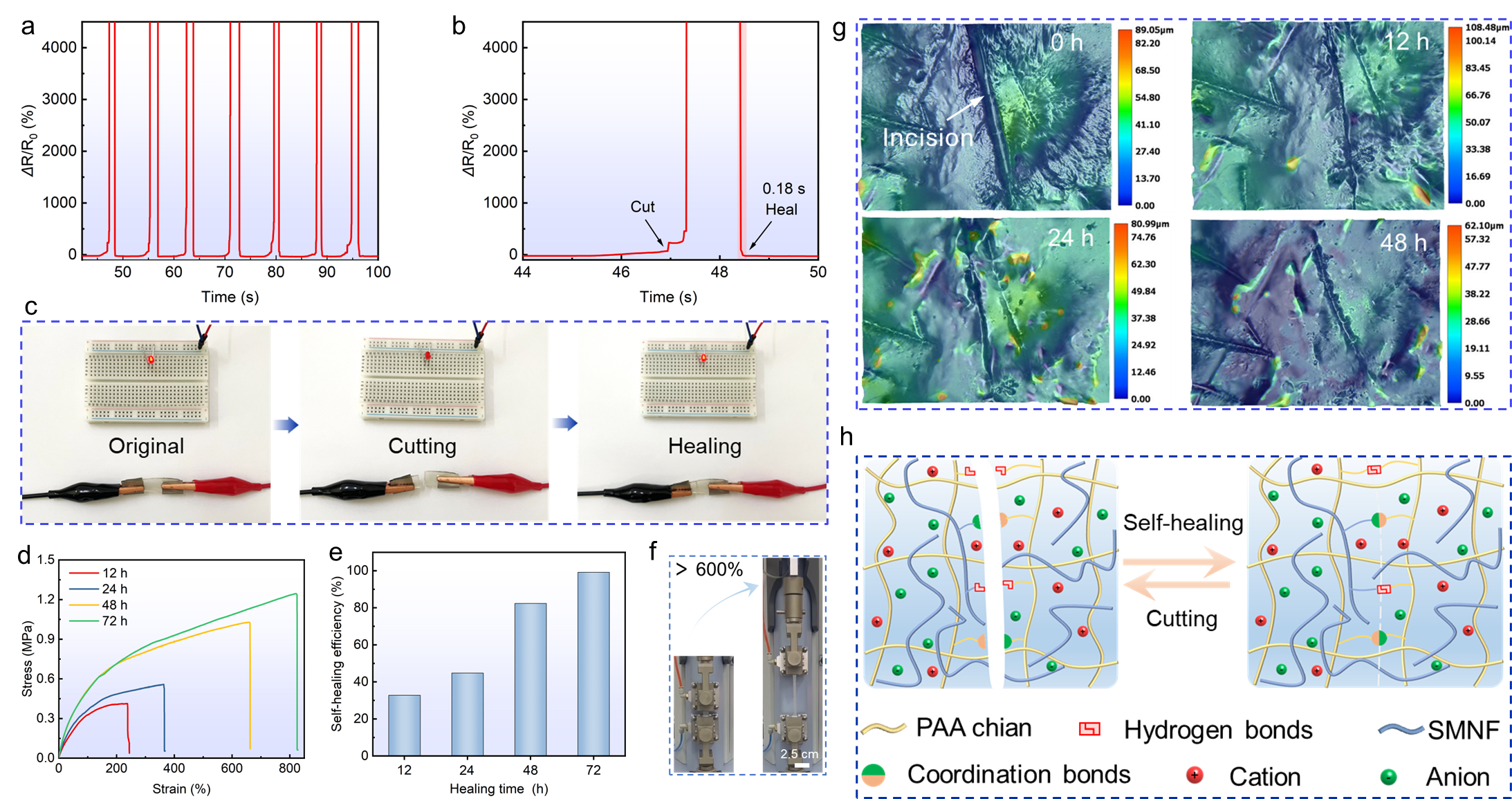


**Figure S25.** Self-healing property of SMNF-Egel-1. (a-b) Real-time resistance changes of SMNF-Egel-1 during the cutting-healing process. (c) Self-healing behavior of SMNF-Egel-1 in the integrated LED bulb circuit. (d) Stress-strain curves of SMNF-Egel-1 after different self-healing times and (e) the corresponding self-healing efficiency. (f) Digital photos of SMNF-Egel-1 stretched to over 600% after healing for 48 h. (g) 3D ultra-depth of field microscopic images of SMNF-Egel-1 at different healing times. (h) Schematic illustration of the possible self-healing mechanism.

The self-healing capability of SMNF-gel-1 was examined in terms of both electrical and mechanical properties. Figure S25a,b show the real-time resistance changes of SMNF-Egel-1 during the cutting-healing cycles. Once cut, the resistance of SMNF-Egel-1 immediately changed to infinite, while the resistance recovered to the original value in a short period of 0.18 s after the two pieces of gel were reconnected. This electrically healing process was also visually observed by integrating SMNF-Egel-1 into a circuit, as demonstrated in Figure S25c and Video S3. When the two pieces of SMNF-Egel-1 were reconnected, the LED bulb reignited and went out as soon as they were separated. The mechanical properties of SMNF-Egel-1 at different healing times were characterized by tensile testing (Figure S25d). With prolonged healing time, both tensile stress and tensile strain increased. As evidenced in Figure S25g, the incision between the two pieces of gel was gradually repaired with prolonged healing time. Although the tensile strain of the recombined gel did not recover to the level of the uncut sample, its tensile stress could recover to a maximum of 1.24 MPa, comparable to that of the original sample. The corresponding self-healing efficiency was ~99.2% in tensile stress after 72 hours at room temperature (Figure S25e), demonstrating its excellent autonomous repair capability. Figure S25f further demonstrates that the repaired SMNF-Egel-1 can withstand a large deformation without fracture. Figure S25h schematically illustrates the self-healing mechanism of SMNF-Egel-1. Typically, at the interface between two separated gels, the dynamic supramolecular networks could be reconstructed due to the abundance and reversibility of hydrogen bonds and coordination bonds between adjacent PAA chains, SMNF, and Ga^3+[1]^ On this basis, a large number of hydrogen bonds and coordination bonds would be re-formed at the interface, thereby ensuring the restoration of the gel networks^[28]^.


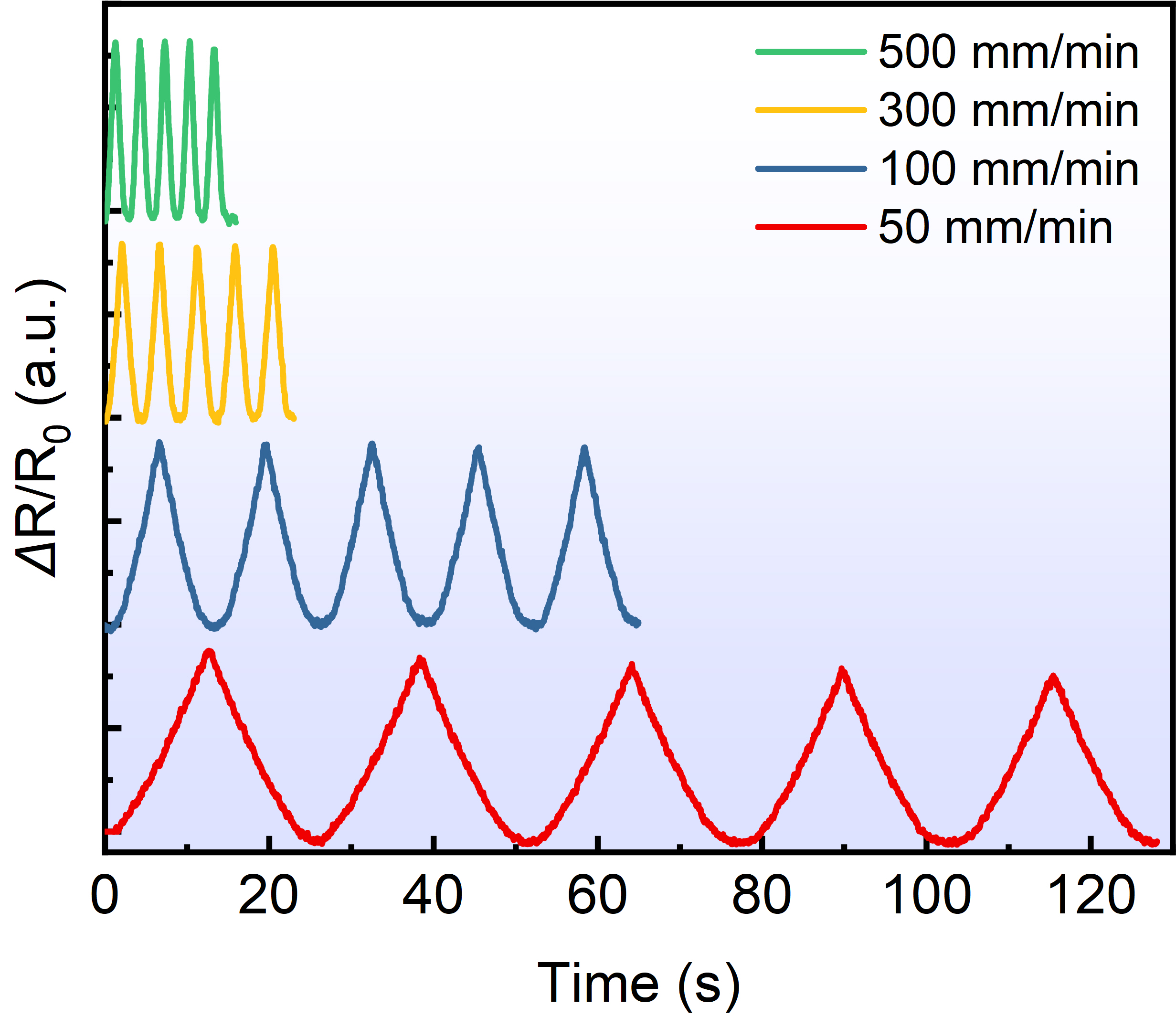


**Figure S26.** *ΔR/R_0_* at different stretching speeds.


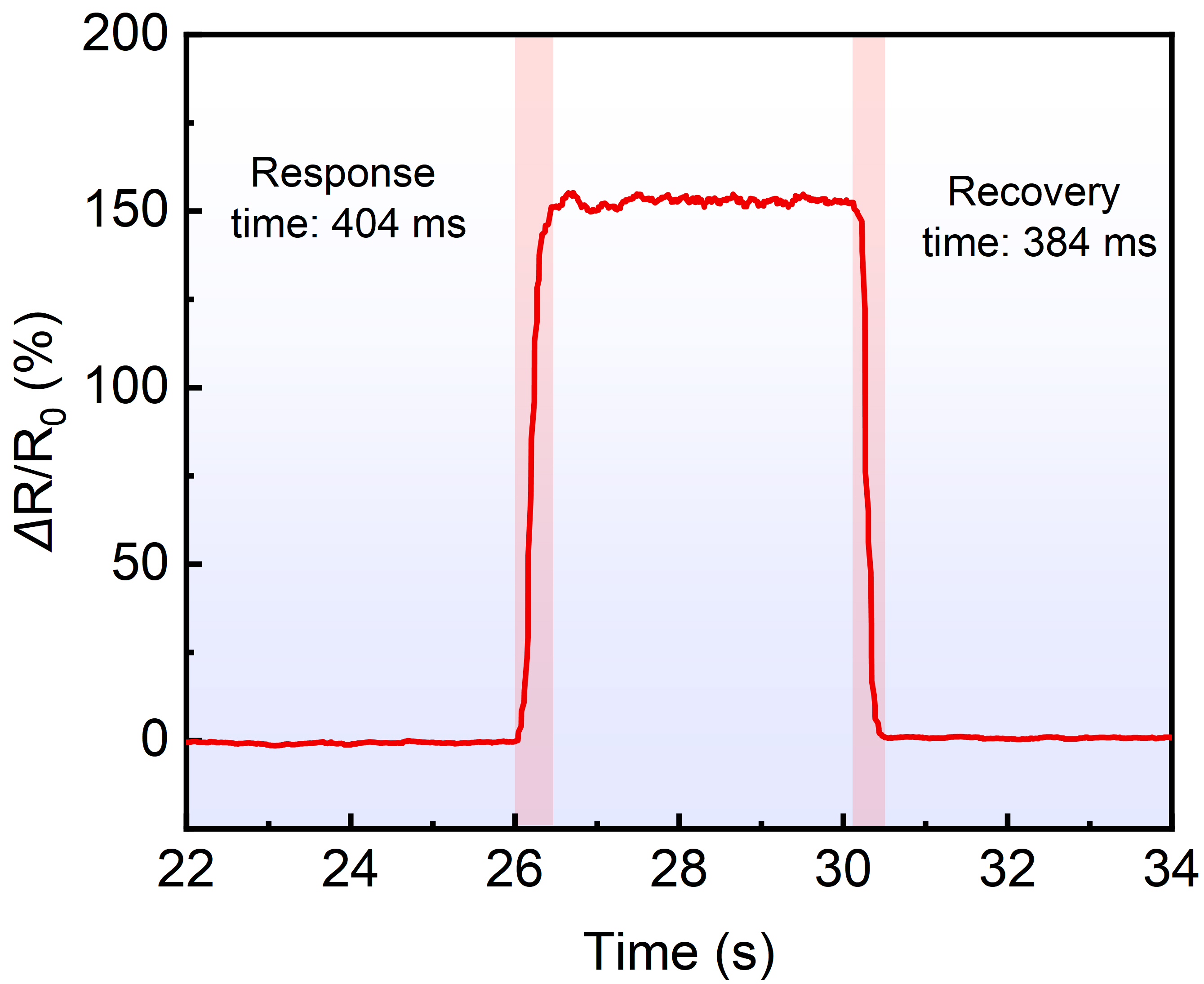


**Figure S27.** Response time and recovery time of SMNF-Egel under 100% strain.


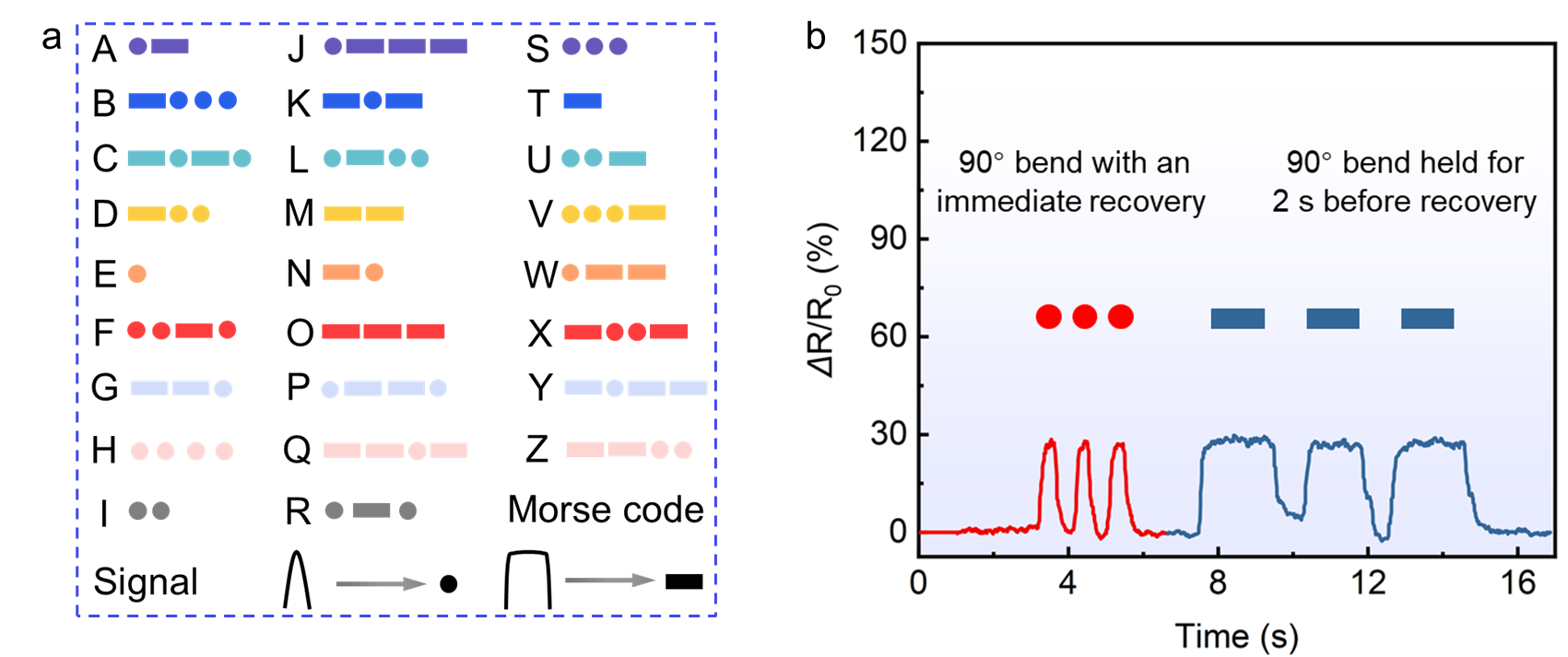


**Figure S28.** (a) Morse code sequences corresponding to the 26 letters of the English alphabet. (b) The SMNF-Egel sensor, based on finger bending movements, encoded “dots” (rapid bending) and “dashes” (bending held for 2 s) as Morse code signals.


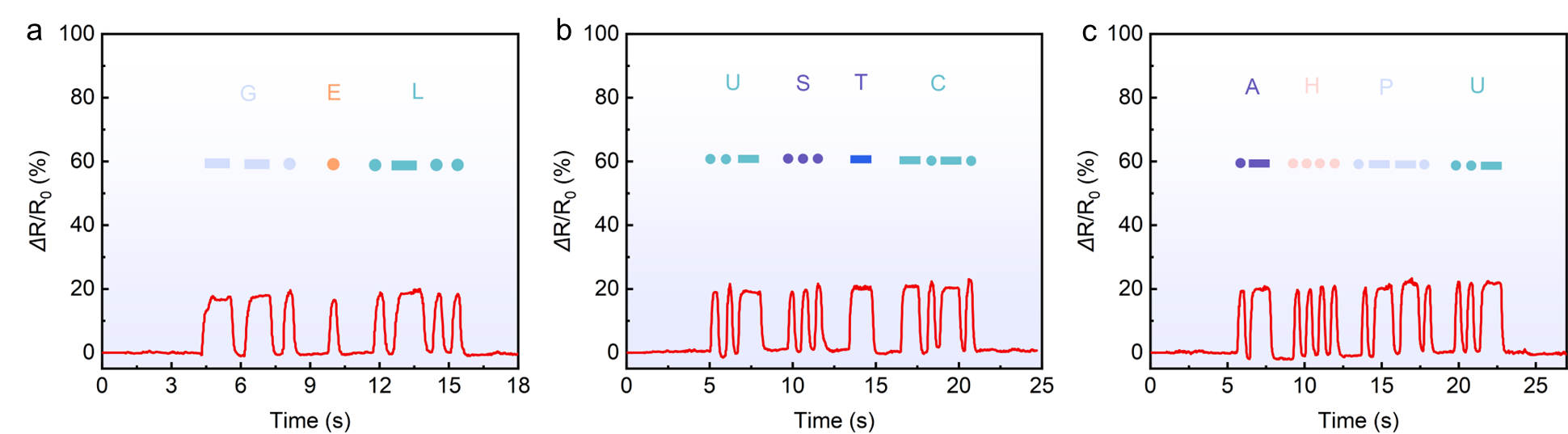


**Figure S29.** The SMNF-Egel sensor can reliably transmit Morse code words such as (a) “GEL,” (b) “USTC,” and (c) “AHPU,” producing distinct and repeatable signal waveforms that highlight its potential for signal transmission.


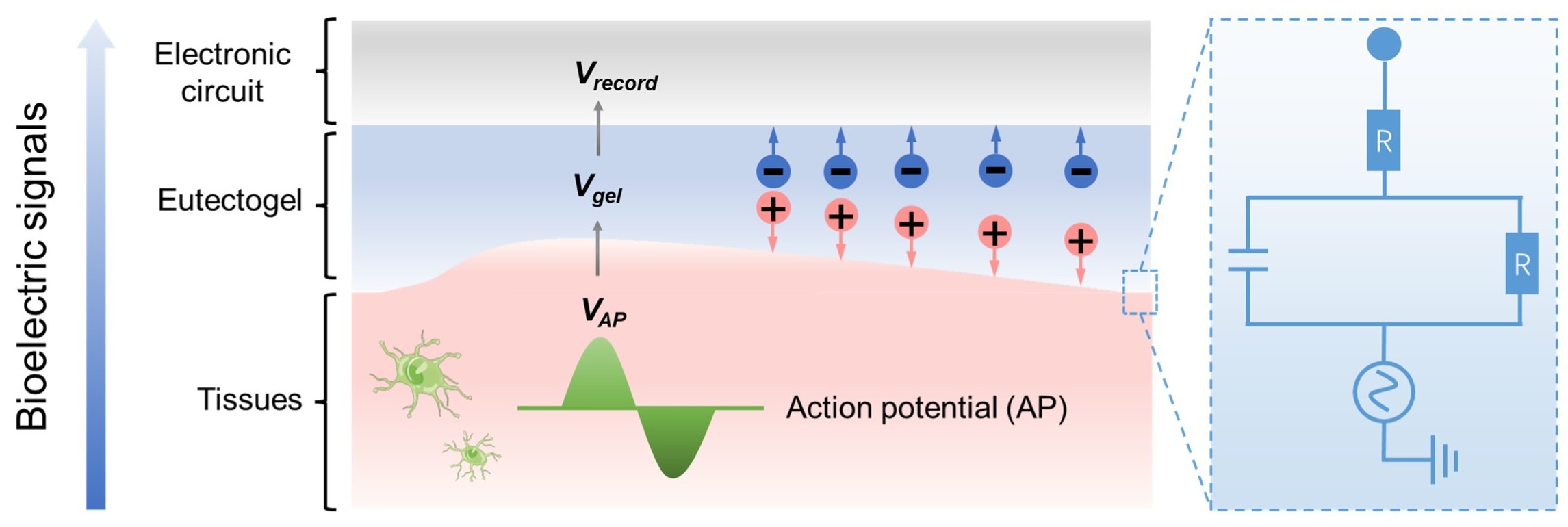


**Figure S30.** Schematic illustration depicting the conduction of bioelectric signals, along with corresponding circuit diagrams showing the interface between tissue and eutectogel.


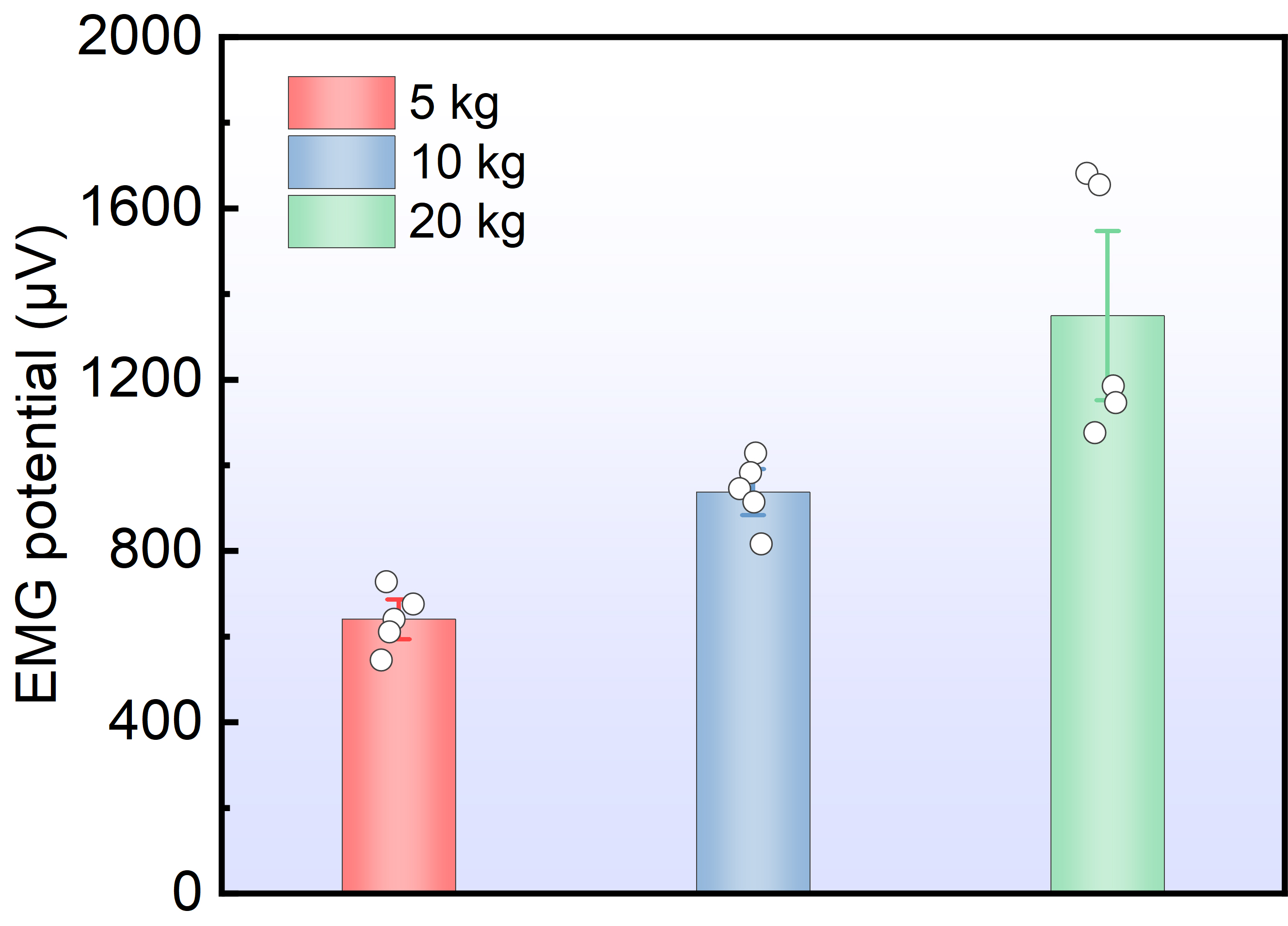


**Figure S31.** Peak-to-peak amplitude of EMG signals recorded by the SMNF-Egel bioelectrode at gripping strengths of 5 kg, 10 kg, and 20 kg, respectively (mean ± SD, n = 5).


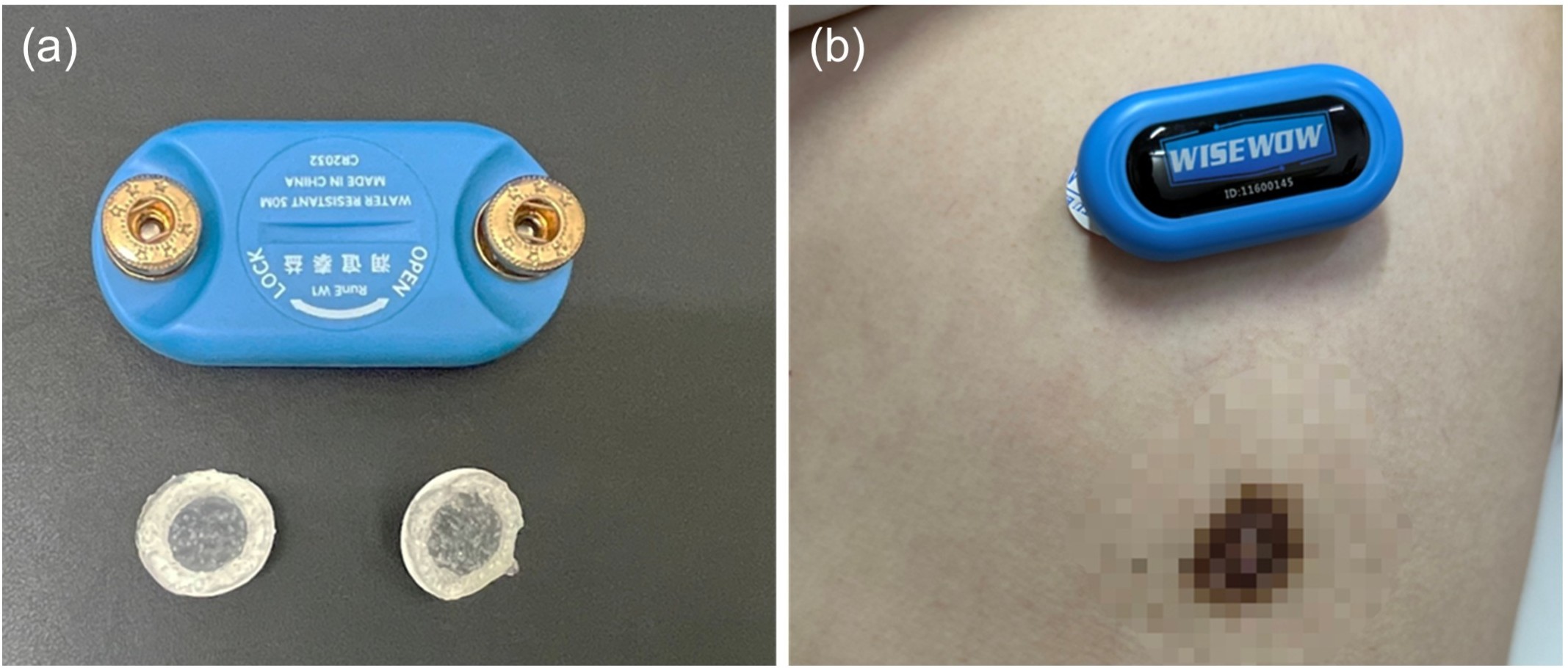


**Figure S32.** Photographs of the measurement device. (a) A photograph demonstrating the wireless module and SMNF-Egel bioelectrode design. (b) A photograph of the measurement device attached to a human chest.

**Supplementary Tables**

**Table S1.** Comparison of our SMNF-Egel with recently reported eutectogels enhanced by other strategies in terms of mechanical properties, conductivity, sensing sensitivity, and process advantages.

| Enhanced cmponents | Tensile stress (MPa) | Toughness (MJ·m^-3^) | Conductivity  (S·m^-1^) | Gauge factor | In-situ generation | Photoinitiator-free polymerization | Reference |
| --- | --- | --- | --- | --- | --- | --- | --- |
| Peptide | 1.04 | 10.6 | 0.382 | 3.17 | NO:  External addition | No:  UV radiation, photoinitiator, cross-linker | ^[29]^ |
| Cllulose nanocrystals (CNC) | 0.496 | 3.44 | 0.38 | 3.67 | NO:  External addition | No:  UV radiation, photoinitiator, cross-linker | ^[30]^ |
| Cellulose mrofibers (CMF) | 0.46 | 13.17 | 0.09 | 3.71 | NO:  External addition | No:  UV radiation, photoinitiator | ^[31]^ |
| Chitin nanofibers  (ChNF) | 0.16 | 1.65 | 0.47 | 3.45 | NO:  External addition | No:  UV radiation, photoinitiator, cross-linker | ^[32]^ |
| Chitin nanocrystals(ChNC) | 0.48 | 17.70 | 0.03 | 0.16 | NO:  External addition | No:  UV radiation, photoinitiator, cross-linker | ^[33]^ |
| MXene | 0.50 | 1.39 | 0.00056 | 2.70 | NO:  External addition | No:  UV radiation, photoinitiator, cross-linker | ^[34]^ |
| RF/PAA DN | 1.04 | 1.07 | 0.10 | 2.63 | NO:  External addition | No:  UV radiation, photoinitiator, cross-linker | ^[35]^ |
| SMNF | 1.25 | 23.09 | 1.51 | 4.44 | Yes | Yes:  Without UV radiation, photoconitiator, cross-linker | This work |

**Table S2.** Mechanical properties comparison table of our work and PDES-based eutectogels in recent studies.

| Strategy | Eutectogels | Stress (MPa) | Strain (%) | Toughness (MJ·m^-3^) | References |
| --- | --- | --- | --- | --- | --- |
| Dual-network | pDMAPS/p(AA-co-HEMAA) | 0.57 | 637 | 2.53 | ^[36]^ |
|  | RF/PAA DN gel | 1.04 | 220 | 1.07 | ^[35]^ |
|  | PDES/PDA | 0.58 | 1200 | 1.4 | ^[37]^ |
|  | TCC-Al^3+^/PDES | 1.11 | 323 | 3.77 | ^[38]^ |
|  | Gelatin/poly(AA-co-HEAA) | 0.39 | 770 | 1.6 | ^[39]^ |
|  | PAM/IC DN gel | 0.13 | 932 | 0.75 | ^[40]^ |
| Nanocomposite | HOFs-PDES | 0.14 | 2744 | 2.69 | ^[41]^ |
|  | COFs-PAA | 0.19 | 2466 | 2.11 | ^[42]^ |
|  | SBA-15/GH-PAA | 0.18 | 1680 | 0.14 | ^[43]^ |
|  | MXene-Gelatin-PHEAA | 0.5 | 940 | 1.39 | ^[34]^ |
| Cellulose-enhanced | BC-PDES | 0.80 | 290 | 1.86 | ^[44]^ |
|  | CMFs-PDES | 0.46 | 3210 | 13.17 | ^[31]^ |
|  | CNC-PDES | 0.59 | 1163 | 3.66 | ^[45]^ |
|  | CNF-PDES | 0.67 | 3200 | 9.35 | ^[46]^ |
|  | TA@CNC-PDES | 0.496 | 2400 | 3.44 | ^[30]^ |
|  | ZnCl_2_/EG/AA-HPC | 0.145 | 144 | 0.147 | ^[47]^ |
| Chitin  -enhanced | ChNF-P(AA-AM) | 0.16 | 1680 | 1.65 | ^[32]^ |
| Protein-enhanced | Peptide crosslinker-PAM-ChCl/EG | 1.04 | 1860 | 10.60 | ^[29]^ |
|  | SF-PDES | 0.96 | 1962 | 3.74 | ^[48]^ |
| Incorporating LM | PDES- LM | 0.13 | 2600 | 2.36 | ^[49]^ |
|  | DAX/LM-PDES | 0.1 | 2860 | 2.83 | ^[28]^ |
| Physical/chemical crosslinking | GMA-OA-CNF/PDES | 0.42 | 419 | 0.11 | ^[50]^ |
|  | ChCl/AA/VAM-CMCS | 1.48 | 526 | 0.39 | ^[51]^ |
|  | PN4 eutectogel | 0.17 | 372 | 0.033 | ^[52]^ |
|  | PAA-KGM-DES | 0.25 | 2403 | 6.24 | ^[53]^ |
| SMNF reconfiguration | SMNF-Egel-1 | 1.25 | 2289 | 23.09 | This work |

**Table S3.** Comparison of the conductivity between our work and other reported representative eutectogels.

| Eutectogels | Material composition | Conductivity (S·m^-1^) | References |
| --- | --- | --- | --- |
| Protein-based  eutectogels | Peptide-enhanced eutectogel | 0.382 | ^[29]^ |
|  | EG/urea/ChCl/ZnCl_2_-SF | 0.123 | ^[54]^ |
|  | Gelatin-ChCl/Gly | 0.08 | ^[55]^ |
| Cellulose-based  eutectogels | CMFs-PDES | 0.09 | ^[31]^ |
|  | ZnCl_2_/EG/PAA-HPC | 0.0366 | ^[47]^ |
|  | TA@CNC-PDES | 0.38 | ^[30]^ |
|  | ZnCl_2_/H_2_O/PAA-cellulose | 0.06 | ^[56]^ |
|  | ZnCl_2_/H_2_O/PAA-cotton | 0.33 | ^[57]^ |
|  | ChCl/PAA-Al^3+^-cellulose | 0.13 | ^[46]^ |
|  | ZnCl_2_/H_2_O/H_3_PO_4_/PAM- cellulose | 1.37 | ^[58]^ |
|  | PDES-MCC | 0.482 | ^[59]^ |
|  | BC-PDES | 0.18 | ^[44]^ |
|  | ChCl/Gly/PS-cellulose | 1.22 | ^[60]^ |
|  | TCC-Al^3+^/PDES | 0.043 | ^[38]^ |
| Chitin-based eutectogels | ChNF-ChCl-P(AA-AM) | 0.47 | ^[32]^ |
|  | ChNC-PAA-ChCl/EG | 0.025 | ^[33]^ |
| PVA-based eutectogels | PVA-ChCl/Gly | 0.0624 | ^[61]^ |
|  | PVA-ChCl/EG | 0.0561 | ^[62]^ |
|  | PVA-ChCl/EG | 0.28 | ^[63]^ |
|  | PVA-ChCl/EG | 0.52 | ^[64]^ |
|  | PVA-ChCl/PA | 0.065 | ^[65]^ |
|  | PVA-ChCl/urea-Ca^2+^ | 0.15 | ^[66]^ |
| Pu-based eutectogels | PU-ChCl/Gly/CA | 0.0038 | ^[67]^ |
|  | WPU-ChCl/EG-GO | 0.025 | ^[68]^ |
|  | WPU-ChCl/Gly-TA | 0.022 | ^[69]^ |
|  | WPU-ChCl/EG | 0.05 | ^[70]^ |
| Silk-based eutectogels | SMNF-Egel-1 | 1.51 | This work |

**Table S4.** Comparison of the gauge factor and maximum working strain with previous reported eutectogels

| Eutectogels | Material composition | Gauge factor (GF) | Maximum working strain (%) | References |
| --- | --- | --- | --- | --- |
| Protein-based  eutectogels | Peptide-enhanced eutectogel | 3.17 | 1000 | ^[29]^ |
|  | Gelatin-ChCl/Gly | 2.45 | 800 | ^[55]^ |
| Cellulose-based  eutectogels | CMFs-PDES | 3.71 | 1300 | ^[31]^ |
|  | TA@CNC-PDES | 3.67 | 300 | ^[30]^ |
|  | ZnCl_2_/H_2_O/PAA- cellulose | 0.73 | 100 | ^[57]^ |
|  | GMA OA-CNF/PDES | 2.83 | 500 | ^[50]^ |
|  | PDES-MCC | 1.78 | 300 | ^[59]^ |
|  | CNC-PDES | 3.51 | 1000 | ^[45]^ |
|  | ChCl/Gly/PS-cellulose | 3.85 | 400 | ^[60]^ |
|  | TCC-Al^3+^/PDES | 1.06 | 200 | ^[38]^ |
| Chitin-based eutectogels | ChNF-ChCl-P(AA-AM) | 3.45 | 500 | ^[32]^ |
|  | ChNC-PAA-ChCl/EG | 0.16 | 1300 | ^[33]^ |
| PVA-based eutectogels | PVA-ChCl/Gly | 1.11 | 100 | ^[61]^ |
|  | PVA-ChCl/Ma | 0.96 | 500 | ^[62]^ |
|  | PVA-ChCl/EG | 1.2 | 500 | ^[63]^ |
|  | ChCl/Gly/H_2_O-PVA | 2.3 | 150 | ^[71]^ |
|  | PVA-ChCl/PA | 1.539 | 500 | ^[65]^ |
|  | PVA-PAA-ChCl/EG | 2.6 | 300 | ^[72]^ |
| PU-based eutectogels | PU-ChCl/Gly/CA | 1.25 | 250 | ^[67]^ |
|  | WPU-ChCl/EG-GO | 1.18 | 100 | ^[68]^ |
| Liquid metal composite eutectogels | LM-PDES | 2.17 | 1000 | ^[49]^ |
| DES-Ionic liquid eutectogels | ChCl-PAA-IL | 0.17 | 500 | ^[73]^ |
|  | P(HEA-co-AA) DES-IL | 1.01 | 500 | ^[74]^ |
| MXene-DES composite eutectogels | MXene/Gel/OHA/DES | 0.71 | 165 | ^[75]^ |
| Silk-based eutectogels | SMNF-Egel | 4.44 | 1200 | This work |

**References**

[1] X. L. Yan, Z. L. Liu, Y. B. Fu, X. Y. Chen, S. Y. Gan, W. W. Yang, S. Chen, L. Liu. Liquid Metal@Silk Fibroin Peptide Particles Initiated Hydrogels with High Toughness, Adhesion, and Conductivity for Portable and Continuous Electrophysiological Monitoring. *Adv. Funct. Mater.* **2025**, *35*, 2420240.

[2] H. Zhao, S. Wang, T. F. Li, Y. Liu, Y. Tang, Z. B. Zhang, H. F. Li, Y. Li, X. X. Li, G. L. Li, X. J. Liu, Q. Tian, Z. Y. Liu. Stretchable Multi-Channel Ionotronic Electrodes for In Situ Dual-Modal Monitoring of Muscle-Vascular Activity. *Adv. Funct. Mater.* **2024**, *34*, 2308686.

[3] T. Li, H. B. Qi, C. C. Zhao, Z. M. Li, W. Zhou, G. J. Li, H. Zhuo, W. Zhai. Robust Skin-Integrated Conductive Biogel for High-Fidelity Detection under Mechanical Stress. *Nat. Commun.* **2025**, *16*, 88.

[4] M. L. Xia, J. W. Liu, B. J. Kim, Y. J. Gao, Y. L. Zhou, Y. J. Zhang, D. X. Cao, S. F. Zhao, Y. Li, J. H. Ahn. Kirigami-Structured, Low-Impedance, and Skin-Conformal Electronics for Long-Term Biopotential Monitoring and Human-Machine Interfaces. *Adv. Sci.* **2024**, *11*, 2304871.

[5] M. D. Hanwell, D. E. Curtis, D. C. Lonie, T. Vandermeersch, E. Zurek, G. R. Hutchison. Avogadro: An Advanced Semantic Chemical Editor, Visualization, and Analysis Platform. *J. Cheminf.* **2012**, *4*, 17.

[6] D. L. Barreiro, Z. Martín-Moldes, J. J. Yeo, S. Shen, M. J. Hawker, F. J. Martin-Martinez, D. L. Kaplan, M. J. Buehler. Conductive Silk-Based Composites Using Biobased Carbon Materials. *Adv. Mater.* **2019**, *31*, 1904720.

[7] D. L. Barreiro, Z. Martín-Moldes, A. B. Fernández, V. Fitzpatrick, D. L. Kaplan, M. J. Buehler. Molecular Simulations of the Interfacial Properties in Silk-Hydroxyapatite Composites. *Nanoscale* **2022**, *14*, 10929.

[8] J. A. Lemkul. Introductory Tutorials for Simulating Protein Dynamics with GROMACS. *J. Phys. Chem. B* **2024**, *128*, 9418.

[9] Y. Gao, C. M. Zhang, X. W. Wang, T. Zhu. A Test of AMBER Force Fields in Predicting the Secondary Structure of α-Helical and β-Hairpin Peptides. *Chem. Phys. Lett.* **2017**, *679*, 112.

[10] Q. S. Li, G. Chen, Y. J. Cui, S. B. Ji, Z. Y. Liu, C. J. Wan, Y. P. Liu, Y. H. Lu, C. X. Wang, N. Zhang, Y. Cheng, K. Q. Zhang, X. D. Chen. Highly Thermal-Wet Comfortable and Conformal Silk-Based Electrodes for on-Skin Sensors with Sweat Tolerance. *ACS Nano* **2021**, *15*, 9955.

[11] G. Chen, N. Matsuhisa, Z. Y. Liu, D. P. Qi, P. Q. Cai, Y. Jiang, C. J. Wan, Y. J. Cui, W. R. Leow, Z. J. Liu, S. X. Gong, K. Q. Zhang, Y. Cheng, X. D. Chen. Plasticizing Silk Protein for on‐Skin Stretchable Electrodes. *Adv. Mater.* **2018**, *30*, 1800129.

[12] D. A. Case, T. E. Cheatham, 3rd, T. Darden, H. Gohlke, R. Luo, K. M. Merz, Jr., A. Onufriev, C. Simmerling, B. Wang, R. J. Woods. The Amber Biomolecular Simulation Programs. *J. Comput. Chem.* **2005**, *26*, 1668.

[13] K. G. Sprenger, V. W. Jaeger, J. Pfaendtner. The General AMBER Force Field (GAFF) Can Accurately Predict Thermodynamic and Transport Properties of Many Ionic Liquids. *J. Phys. Chem. B* **2015**, *119*, 5882.

[14] M. Fleck, S. Darouich, J. Pleiss, N. Hansen, M. B. M. Spera. Physics-Informed Multifidelity Gaussian Process: Modeling the Effect of Water and Temperature on the Viscosity of a Deep Eutectic Solvent. *J. Chem. Inf. Model.* **2025**, *65*, 3999.

[15] T. Lu, F. W. Chen. Multiwfn: A Multifunctional Wavefunction Analyzer. *J. Comput. Chem.* **2012**, *33*, 580.

[16] T. Lu. A Comprehensive Electron Wavefunction Analysis Toolbox for Chemists, Multiwfn. *J. Chem. Phys.* **2024**, *161*, 082503.

[17] J. Luo, J. J. Yang, X. R. Zheng, X. Ke, Y. T. Chen, H. Tan, J. S. Li. A Highly Stretchable, Real-Time Self-Healable Hydrogel Adhesive Matrix for Tissue Patches and Flexible Electronics. *Adv. Healthcare Mater.* **2020**, *9*, 1901423.

[18] R. A. Li, K. L. Zhang, G. X. Chen, B. Su, J. F. Tian, M. H. He, F. C. Lu. Green Polymerizable Deep Eutectic Solvent (PDES) Type Conductive Paper for Origami 3D Circuits. *Chem. Commun.* **2018**, *54*, 2304.

[19] R. Li, G. X. Chen, M. H. He, J. F. Tian, B. Su. Patternable Transparent and Conductive Elastomers towards Flexible Tactile/Strain Sensors. *J. Mater. Chem. C* **2017**, *5*, 8475.

[20] J. H. Su, S. H. Li, Y. L. Chen, Y. Y. Cui, M. H. He. 3D Photoprintable Antistatic Materials with Polymerizable Deep Eutectic Solvents. *Ind. Eng. Chem. Res.* **2021**, *60*, 17797.

[21] X. X. Tan, W. C. Zhao, T. C. Mu. Controllable Exfoliation of Natural Silk Fibers into Nanofibrils by Protein Denaturant Deep Eutectic Solvent: Nanofibrous Strategy for Multifunctional Membranes. *Green Chem.* **2018**, *20*, 3625.

[22] H. Yang, P. Wang, Q. Yang, D. Wang, Y. Wang, L. Kuai, Z. Wang. Superelastic and Multifunctional Fibroin Aerogels from Multiscale Silk Micro-Nanofibrils Exfoliated via Deep Eutectic Solvent. *Int. J. Biol. Macromol.* **2023**, *224*, 1412.

[23] Y. L. Hu, L. Liu, J. Yu, Z. G. Wang, Y. M. Fan. Preparation of Natural Multicompatible Silk Nanofibers by Green Deep Eutectic Solvent Treatment. *ACS Sustainable Chem. Eng.* **2020**, *8*, 4499.

[24] W. Huang, S. Ling, C. Li, F. G. Omenetto, D. L. Kaplan. Silkworm Silk-Based Materials and Devices Generated Using Bio-Nanotechnology. *Chem. Soc. Rev.* **2018**, *47*, 6486.

[25] S. H. Lin, C. Ye, W. W. Zhang, A. C. Xu, S. X. Chen, J. Ren, S. J. Ling. Nanofibril Organization in Silk Fiber as Inspiration for Ductile and Damage-Tolerant Fiber Design. *Adv. Fiber Mater.* **2019**, *1*, 231.

[26] J. L. Wang, K. Zhao, Y. B. Zhao, C. Q. Ye. Highly Conductive, Ultratough, and Adhesive Eutectogels with Environmental Tolerance Enabled by Liquid Metal Composites. *Small* **2025**, *21*, 2410806.

[27] M. Q. You, J. Zhou, Y. M. Zao, J. H. Xu, Y. C. Jin, D. G. Li, Z. Y. Xu, C. C. Chen. Green Synthesis Of Multifunctional Wood-Based Eutectogels via Initiator-Free Solar Polymerization. *Chem. Eng. J.* **2025**, *504*, 158902.

[28] J. Y. Yang, Y. Yan, L. Z. Huang, M. G. Ma, M. F. Li, F. Peng, W. W. Huan, J. Bian. Conductive Eutectogels Fabricated by Dialdehyde Xylan/Liquid Metal-Initiated Rapid Polymerization for Multi-Response Sensors and Self-Powered Applications. *ACS Nano* **2025**, *19*, 2171.

[29] Y. Zhang, Y. F. Wang, Y. Guan, Y. J. Zhang. Peptide-Enhanced Tough, Resilient and Adhesive Eutectogels for Highly Reliable Strain/Pressure Sensing under Extreme Conditions. *Nat. Commun.* **2022**, *13*, 6671.

[30] X. R. Zhang, Q. J. Fu, Y. C. Wang, H. A. Zhao, S. W. Hao, C. Ma, F. Xu, J. Yang. Tough Liquid-Free Ionic Conductive Elastomers with Robust Adhesion and Self-Healing Properties for Ionotronic Devices. *Adv. Funct. Mater.* **2024**, *34*, 2307400.

[31] X. Sun, Y. L. Zhu, J. Y. Zhu, K. Le, P. Servati, F. Jiang. Tough and Ultrastretchable Liquid-Free Ion Conductor Strengthened by Deep Eutectic Solvent Hydrolyzed Cellulose Microfibers. *Adv. Funct. Mater.* **2022**, *32*, 2202533.

[32] X. M. Li, L. N. Xu, J. L. Gao, M. Q. Yan, Q. Y. Wang. Highly Stretchable, Tough, and Transparent Chitin Nanofiber-Reinforced Multifunctional Eutectogels for Self-Powered Wearable Sensors. *ACS Sens.* **2025**, *10*, 886.

[33] S. Wang, X. S. Du, X. Cheng, Z. L. Du, Z. Y. Zhang, H. B. Wang. Ultrahigh Stretchable, Highly Transparent, Self-Adhesive, and Environment-Tolerant Chitin Nanocrystals Engineered Eutectogels toward Multisignal Sensors. *ACS Appl. Mater. Interfaces* **2024**, *16*, 45537.

[34] B. Y. Guo, M. M. Yao, S. Chen, Q. Y. Yu, L. Liang, C. J. Yu, M. Liu, H. Z. Hao, H. Zhang, F. L. Yao, J. J. Li. Environment-Tolerant Conductive Eutectogels for Multifunctional Sensing. *Adv. Funct. Mater.* **2024**, *34*, 2315656.

[35] Q. Quan, C. L. Fan, N. A. Pan, M. H. Zhu, T. Zhang, Z. Wang, Y. Dong, Y. K. Wu, M. Tang, X. Y. Zhou, M. Z. Chen. Tough and Stretchable Phenolic-Reinforced Double Network Deep Eutectic Solvent Gels for Multifunctional Sensors with Environmental Adaptability. *Adv. Funct. Mater.* **2023**, *33*, 2303381.

[36] Z. Z. Ma, J. L. Zhang, Z. L. Ma, M. H. Lou, P. J. Zou, H. Q. Wang, L. C. Jia. A Transparent, Tough, Highly Stretchable and Self-Adhesive Zwitterionic Dual-Network Eutectogel for Wearable Flexible Sensors. *J. Mater. Chem. A* **2025**, *13*, 9418.

[37] Z. Y. Hua, G. X. Chen, K. Zhao, R. Li, M. H. He. A Repeatable Self-Adhesive Liquid-Free Double-Network Ionic Conductor with Tunable Multifunctionality. *ACS Appl. Mater. Interfaces* **2022**, *14*, 22418.

[38] Y. F. Lan, W. W. Liu, Z. M. Lv, Z. J. Li, A. Dufresne, L. H. Fu, B. F. Lin, C. H. Xu, B. Huang. Liquid-Free, Tough and Transparent Ionic Conductive Elastomers Based on Nanocellulose for Multi-Functional Sensors and Triboelectric Nanogenerators. *Nano Energy* **2024**, *129*, 110047.

[39] X. H. Zhang, X. H. Li, J. X. Yang, Y. D. Liu. Stretchable, Fatigue-Resistant, and Temperature-Tolerant Multifunctional Dual-Network Eutectogel Based on Metal Salt-Based Deep Eutectic Solvent for Strain Sensors and Triboelectric Nanogenerators. *Chem. Eng. J.* **2025**, *522*, 167850.

[40] M. Lu, L. B. Shen, H. X. Su, B. Li, L. Y. Wang, W. W. Yu. Highly Ionic Conductive, Elastic, and Biocompatible Double-Network Composite Gel for Epidermal Biopotential Monitoring and Wearable Sensing. *J. Colloid Interface Sci.* **2025**, *684*, 272.

[41] G. H. Liu, D. Wang, H. Li, K. Kong, K. K. Xu, B. Y. Liu, R. H. Wang. Energy Dissipation Mediated by Multiple Noncovalent Interactions in Hydrogen‐Bonded Organic Frameworks‐Based Hydrogels for Wearable Gesture‐to‐Recognition Translation. *Angew. Chem., Int. Ed.* **2025**, *64*, e202514750.

[42] D. Wang, Z. Y. Liu, H. Li, G. H. Liu, X. J. Li, M. W. Pan, R. H. Wang. Robust Hydrogel Sensors Induced by Intermolecular Mechanical Interlocking of Covalent Organic Frameworks for Non-Invasive Health Monitoring. *Adv. Mater.* **2025**, *37*, e20271.

[43] X. Wang, Z. W. Guo, G. H. Yang, X. X. Ji, G. Lyu. Highly Stretchable Eutectogel for Strain Sensors and Bioelectrodes under Extreme Environments. *Small* **2025**, 21, e09447.

[44] M. Wang, R. N. Li, X. Feng, C. Dang, F. L. Dai, X. Q. Yin, M. H. He, D. T. Liu, H. S. Qi. Cellulose Nanofiber-Reinforced Ionic Conductors for Multifunctional Sensors and Devices. *ACS Appl. Mater. Interfaces* **2020**, *12*, 27545.

[45] X. K. Li, J. Z. Liu, Q. Q. Guo, X. X. Zhang, M. Tian. Polymerizable Deep Eutectic Solvent‐Based Skin‐Like Elastomers with Dynamic Schemochrome and Self‐Healing Ability. *Small* **2022**, *18*, 2201012.

[46] Y. F. Wu, X. F. Zhang, Y. H. Bai, M. J. Yu, J. F. Yao. Cellulose-Reinforced Highly Stretchable and Adhesive Eutectogels as Efficient Sensors. *Int. J. Biol. Macromol.* **2024**, *265*, 131115.

[47] C. W. Lu, X. Y. Wang, Y. Shen, S. J. Xu, C. X. Huang, C. P. Wang, H. J. Xie, J. F. Wang, Q. Yong, F. X. Chu. Skin-Like Transparent, High Resilience, Low Hysteresis, Fatigue-Resistant Cellulose-Based Eutectogel for Self-Powered E-Skin and Human-Machine Interaction. *Adv. Funct. Mater.* **2024**, 34, 2311502.

[48] Y. M. Yan, W. J. Deng, D. Xie, J. Hu. Silk Fibroin Hydrogel for Pulse Waveform Precise and Continuous Perception. *Adv. Healthcare Mater.* **2025**, *14*, 2403637.

[49] M. Wang, Z. B. Lai, X. L. Jin, T. L. Sun, H. C. Liu, H. S. Qi. Multifunctional Liquid-Free Ionic Conductive Elastomer Fabricated by Liquid Metal Induced Polymerization. *Adv. Funct. Mater.* **2021**, *31*, 2101957.

[50] S. N. Wang, X. Y. Cheng, E. Q. Zhu, T. Q. Li, L. L. Zhang, Y. M. Fan, Z. G. Wang. One-Pot Preparation of Physical and Chemical Double-Cross-Linked Nanocellulose/Poly (Deep Eutectic Solvent) Conductive Elastomers in Acidic Deep Eutectic Solvent (DES) System for 3D-Printable Information-Storage Expansion. *Chem. Eng. J.* **2024**, *488*, 151066.

[51] C. W. Lu, C. P. Wang, J. F. Wang, Q. Yong, F. X. Chu. Integration of Hydrogen Bonding Interaction and Schiff-Base Chemistry toward Self-Healing, Anti-Freezing, and Conductive Elastomer. *Chem. Eng. J.* **2021**, *425*, 130652.

[52] C. C. Yang, B. Xu, Y. Tang, Z. Q. Liu, H. Y. Mi, F. J. Guo, X. Q. Liao, J. S. Qiu. Super Seather-Resistant and Self-Healing Eutectogels via Dynamic Interactions for Wide-Range Healthcare and Highly Adaptive Human-Machine Interfaces. *Adv. Funct. Mater.* **2025**, doi.org/10.1002/adfm.202507051.

[53] T. Liu, Z. Z. Nong, Y. Li, Z. H. Liu, Z. Y. Fan, H. S. Liu, Q. Liu, J. Y. Qiu, Q. W. Wang, Z. Z. Liu. Eutectogel Skin Electrodes with Superior Adhesion, Sweat Resistance, and Long-Term Stability for Electrophysiological Signal Monitoring. *Adv. Funct. Mater.* **2025**, *35*, e03568.

[54] Z. X. Li, X. E. Xu, Z. Jiang, J. Y. Chen, J. P. Tu, X. L. Wang, C. D. Gu. A Silk Protein-Based Eutectogel as A Freeze-Resistant and Flexible Electrolyte for Zn-Ion Hybrid Supercapacitors. *ACS Appl. Mater. Interfaces* **2022**, *14*, 44821.

[55] Y. F. Li, Z. H. Qin, P. He, M. Q. Si, L. F. Zhu, N. Li, X. J. Shi, G. Q. Hao, T. F. Jiao, X. M. He. Fully Degradable Protein Gels with Superior Mechanical Properties and Durability: Regulation of Hydrogen Bond Donors. *Adv. Mater.* **2025,** *37*, 2506577.

[56] J. X. Zhu, C. Y. Shao, S. W. Hao, K. Xue, J. F. Zhang, Z. W. Sun, L. P. Xiao, W. F. Ren, J. Yang, B. B. Cao, R. C. Sun. Green Synthesis of Multifunctional Cellulose-Based Eutectogel Using a Metal Salt Hydrate-Based Deep Eutectic Solvent for Sustainable Self-Powered Sensing. *Chem. Eng. J.* **2025**, *506*, 159636.

[57] Y. F. Wu, X. F. Zhang, M. J. Li, M. J. Yu, J. F. Yao. Self-Healing and Wide Temperature-Tolerant Cellulose-Based Eutectogels for Reversible Humidity Detection. *Langmuir* **2024**, *40*, 5288.

[58] W. Chen, J. Ma, D. H. Yu, N. Li, X. X. Ji. Transparent, Super Stretchable, Freezing-Tolerant, Self-Healing Ionic Conductive Cellulose Based Eutectogel for Multi-Functional Sensors. *Int. J. Biol. Macromol.* **2024**, *266*, 131129.

[59] Y. X. Chen, H. X. Zhang, G. H. Hong, L. Li, Q. Qu. Human Bio-Electric Generator: Self-Powered Cellulose-Based Wearable Sensor with Ultra-Stretchability and Low-Grade Body Heat Harvesting. *Carbohydr. Polym.* **2025**, *355*, 123349.

[60] Q. W. Lu, H. F. Li, Z. J. Tan. Natural Cellulose Reinforced Multifunctional Eutectogels for Wearable Sensors and Epidermal Electrodes. *Carbohydr. Polym.* **2025**, *348*, 122939.

[61] H. Zhang, N. Tang, X. Yu, M. H. Li, J. Hu. Strong and Tough Physical Eutectogels Regulated by the Spatiotemporal Expression of Non-Covalent Interactions. *Adv. Funct. Mater.* **2022**, *32*, 2206305.

[62] X. S. Huo, J. L. Wang, Z. H. Cong, C. Liu, C. J. Cai, Y. X. Wang, X. Q. Zhang, C. N. Li, S. Lan, J. Niu. Strong and Tough Eutectogels with Broad-Range Tunable Mechanical Properties via the Hydrogen Bond Network-Specific Effect. *Adv. Funct. Mater.* **2025**, *35*, 2422464.

[63] T. H. Vo, P. K. Lam, R. M. Chuang, F. K. Shieh, Y. J. Sheng, H. K. Tsao. One-Step, Additive-Free Fabrication of Highly Stretchable and Ultra-Tough Physical Polyvinyl Alcohol-Based Eutectogels for Strain Sensors. *Chem. Eng. J.* **2024**, *493*, 152877.

[64] S. Du, X. D. Chen, M. M. Li, B. L. Peng, Q. Lyu, L. B. Zhang, J. T. Zhu. Ultratough and Highly Conductive Supramolecular Poly (Vinyl Alcohol) Eutectogels via a Sequentially Enhanced Strategy. *Adv. Funct. Mater.* **2025**, *35*, 2409726.

[65] Y. Z. Shao, C. Dang, H. B. Qi, Z. Y. Liu, H. R. Pei, T. Q. Lu, W. Zhai. Polyfunctional Eutectogels with Multiple Hydrogen-Bond-Shielded Amorphous Networks for Soft Ionotronics. *Matter* **2024**, *7*, 4076.

[66] J. P. Zhang, M. Q. Zhang, H. X. Wan, J. P. Zhou, A. Lu. Coordinatively Stiffen and Toughen Polymeric Gels via the Synergy of Crystal-Domain Cross-Linking and Chelation Cross-Linking. *Nat. Commun.* **2025**, *16*, 320.

[67] R. Zhou, Y. Jin, W. H. Zeng, H. Y. Jin, L. J. Shi, L. Bai, X. Shang. Versatile Quasi-Solid Ionic Conductive Elastomer Inspired by Desertification Control Strategy for Soft Iontronics. *Adv. Funct. Mater.* **2023**, *33*, 2301921.

[68] T. D. Chen, R. Luo, Y. F. Liu, L. M. Ma, Z. P. Li, C. H. Tao, S. R. Yang, J. Q. Wang. Two-Dimensional Nanosheet-Enhanced Waterborne Polyurethane Eutectogels with Ultrastrength and Superelasticity for Sensitive Strain Sensors. *ACS Appl. Mater. Interfaces* **2022**, *14*, 40276.

[69] S. Wang, H. L. Cheng, B. Yao, H. He, L. Zhang, S. Z. Yue, Z. R. Wang, J. Y. Ouyang. Self-Adhesive, Stretchable, Biocompatible, and Conductive Nonvolatile Eutectogels as Wearable Conformal Strain and Pressure Sensors And Biopotential Electrodes for Precise Health Monitoring. *ACS Appl. Mater. Interfaces* **2021**, *13*, 20735.

[70] Y. L. Zhao, H. L. Cheng, Y. X. Li, J. C. Rao, S. Z. Yue, Q. J. Le, Q. Qian, Z. Liu, J. Y. Ouyang. Quasi-Solid Conductive Gels with High Thermoelectric Properties and High Mechanical Stretchability Consisting of a Low Cost And Green Deep Eutectic Solvent. *J. Mater. Chem. A* **2022**, *10*, 4222.

[71] Y. Wang, Y. Wang, L. F. Yan. Deep Eutectic Solvent-Induced Microphase Separation and Entanglement of PVA Chains for Tough and Reprocessable Eutectogels for Sensors. *Langmuir* **2022**, *38*, 12189.

[72] Y. Wang, J. K. Wang, Z. Z. Ma, L. F. Yan. A Highly Conductive, Self-Recoverable, and Strong Eutectogel of a Deep Eutectic Solvent with Polymer Crystalline Domain Regulation. *ACS Appl. Mater. Interfaces* **2021**, *13*, 54409.

[73] K. Xue, C. Y. Shao, J. Yu, H. M. Zhang, B. Wang, W. F. Ren, Y. B. Cheng, Z. X. Jin, F. Zhang, Z. K. Wang, R. C. Sun. Initiatorless Solar Photopolymerization of Versatile and Sustainable Eutectogels as Multi-Response and Self-Powered Sensors for Human-Computer Interface. *Adv. Funct. Mater.* **2023**, *33*, 2305879.

[74] Y. P. Zheng, T. Y. Cui, J. W. Wang, Y. Q. Chen, M. Y. Ou, H. L. He, Y. Hu, Z. Gui. Highly Stretchable, Low-Hysteresis, and Robust Polymeric Gels Enabled by Solvent Engineering for Wireless Sensing and Encrypted Communication. *Chem. Eng. J.* **2025**, *515*, 163610.

[75] H. Zhou, M. Yang, W. He, Y. Gao, X. Zhu, J. Wu, L. Zhang, P. Wan. A Thermoresponsive Bioadhesive Mxene Hydrogel for Intelligent Brain-Machine Interaction Sensing. *Matter* **2025**, *8*, 102150.
